# Metalloid Tin Clusters, Sn_*n*_ *t*Bu_m_, with α‑Sn-Like Structural Motifs

**DOI:** 10.1021/acs.inorgchem.6c01824

**Published:** 2026-06-25

**Authors:** Beate G. Steller, Clara A. Roller, Steffen Klenner, Joshua Wiethölter, Rainer Pöttgen, Michaela Flock, Roland C. Fischer

**Affiliations:** † Institute of Inorganic Chemistry, Graz University of Technology, Stremayrgasse 9/V, 8010 Graz, Austria; ‡ Institut für Anorganische und Analytische Chemie, 271671 Universität Münster, Corrensstraße 30, 48149 Münster, Germany

## Abstract

Metalloid tin clusters represent an intriguing class of compounds that bridge molecular chemistry and the solid state, yet access to diverse examples remains limited. Herein, we present a mild, modular hydrostannolysis-type strategy that enables the synthesis of tin-based clusters stabilized by fairly small *tert*-butyl substituents, thereby overcoming the ligand-imposed structural limitations of established methodologies. The approach gave access to a series of clusters of different core sizes, including Sn_11_ *t*Bu_12_, featuring a [1.1.1]­propellane-type backbone, as well as larger Sn_15_ *t*Bu_14_ and Sn_16_ *t*Bu_16_ derivatives adopting α-Sn-like core motifs with chair arrangement reminiscent of the diamond-type structure. Experimental characterization (X-ray crystallography and ^119^Sn Mössbauer spectroscopy) and computational analyses (NBO and QTAIM) corroborate near-zero charges at the apical tin centers; the frontier orbitals show pronounced multicenter character across the tin framework, and several deeper-lying orbitals exhibit “superatom”-like character. Additionally, this synthetic protocol also afforded a unique, heterobimetallic Sn/Pb spiro compound, revealing the compatibility of the method with multiple group 14 elements.

## Graphical abstract

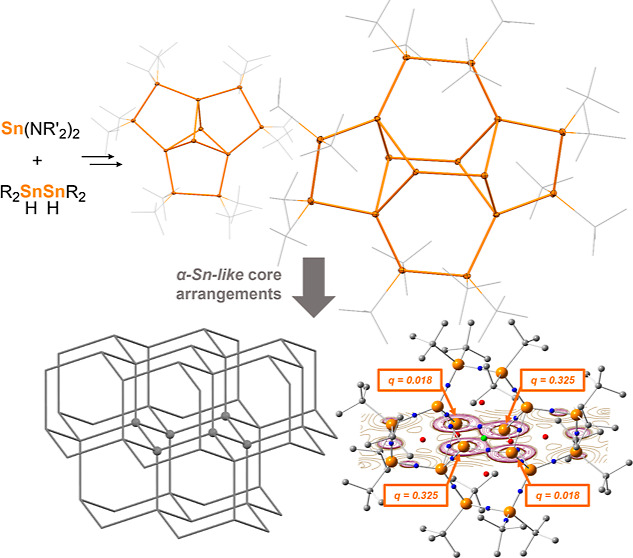

## Introduction

Metalloid clusters—molecular compounds comprising unsubstituted (“*naked*”) metal/metalloid atoms connected solely by element–element bonds alongside ligand-coordinated fragments—are conceptually significant owing to their delocalized bonding, intricate electronic structures, and bulk-like core architectures, which also render them aesthetically appealing.^1–5^ Structural motifs resembling bulk-metal structures, in particular, provide molecular platforms for probing surface chemistry under conditions amenable to detailed spectroscopic and crystallographic analysis.^6,7^ In contrast to the broader compound scope established for aluminum- and gallium-containing systems,^8^ structurally characterized uncharged tetrel clusters remain sparse, with tin-based examples especially underrepresented (selected motifs are shown in Figure 1);^9–16^ notably, no example with an α-Sn-like (cubic diamond-type) core motif has been isolated thus far. Most reported tin-based compounds rely on highly encumbered ligand environments^1,17–20^ that seem to largely dictate core architecture—often independent of the chosen synthetic route—thereby intrinsically limiting accessible geometries.

**Figure 1:**
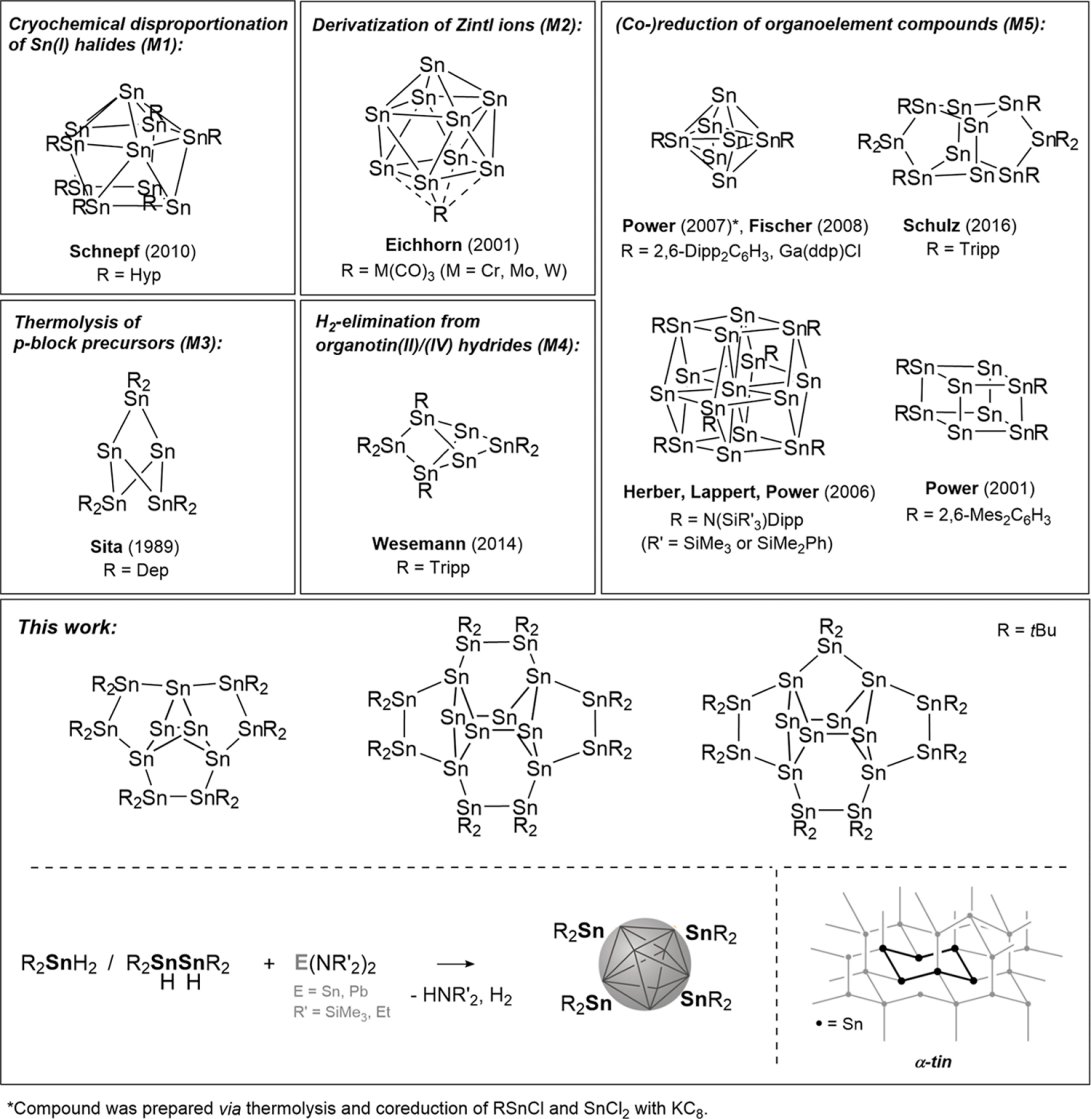
Selection of representative structural motifs observed in uncharged tin-based cluster compounds.

The recurring E_9_ (E = Ge, Sn) motif is a prominent example, obtained both via (partial) substitution of the parent Zintl ions, E_9_^4–^ (E = Ge,^21,22^ Sn),^23^ with electron-rich electrophiles and from reactions of E­(I) halides (E = Ge,^24^ Sn^25^) with silanides (LiSiR_3_ (R = Si­(SiR′_3_)_3_)), in each case affording species with identical backbones ([E_9_R_n_]^m–/•^).^21–26^ This example underscores that ligand choice, rather than the synthetic route, dominates structural outcome, likely limiting access to genuinely new core motifs. The absence of molecular tin clusters resembling the bulk structure contrasts sharply with the lighter congeners: Diamondoids stand out as prominent examples, with adamantane—the parent carbon cage and finite analogue of the cubic diamond structure—studied and functionalized extensively^27–29^ since its landmark synthesis by Schleyer.^30^ Heavier variants of the adamantane backbone are far less common; Marschner’s sila-adamantane^31,32^ and mixed silagerma-adamantanes^33–35^ remain the only well-characterized examples. This scarcity underscores the synthetic challenge of accessing tetrel clusters that mimic element structures and highlights the need for milder, modular strategies enabling flexible ligand variation. Established routes for accessing group 14 clusters include cryochemical disproportionation of metastable Sn­(I) halides [M1 (see Figure 1), pioneered by Schnöckel and Schnepf],^19,36–38^ derivatization of Zintl ions^22,23,39–41^ (M2), thermolysis of p-block precursors^9,11^ (M3), dihydrogen elimination from organotin­(II)- and organotin­(IV)-hydrides (M4, established mainly by Wesemann’s group),^10,42^ and (co)­reduction of organoelement compounds^12,43–46^ (M5). While effective for specific systems, these methods typically require harsh conditions, specialized equipment or highly robust ligands, thereby limiting accessible structural diversity.

## Results and Discussion

Building on a hydrostannolysis-type strategy—the reaction of diorganotin dihydrides and low-oxidation state bismuth triamides toward mixed Sn/Bi systems^47^—we now extend this approach to homometallic Sn/Sn and heterobimetallic Sn/Pb compounds. It accommodates relatively small ligand environments, such as *tert*-butyl (as shown herein),[Fn fn1] and grants access to α-tin-like core motifs previously inaccessible by other methods.

### Reaction of *t*Bu_2_HSnSnH*t*Bu_2_ with Sn­[N­(SiMe_3_)_2_]_2_: Isolation of Functionalized Cyclotetrastannane 1

Given our previous success in employing the HMDS (hexamethyl disilazide) derivatives of respective element amides in this protocol, we first turned to the analogous Lappert’s stannylene, Sn­[N­(SiMe_3_)_2_]_2_, as a readily accessible low-valent tin amide precursor. Initial conversions of *t*Bu_2_HSnSnH*t*Bu_2_ with Sn­[N­(SiMe_3_)_2_]_2_ (3:5 ratio) in diglyme led to a gradual color change from pale orange to red, eventually fading to brown. After 18 days, pale yellow crystals of Sn_4_ *t*Bu_6_(N­(SiMe_3_)_2_)­(H) (**1**) were isolated (49% yield, based on *t*Bu_2_HSnSnH*t*Bu_2_, see Scheme 1).

**Scheme 1:**
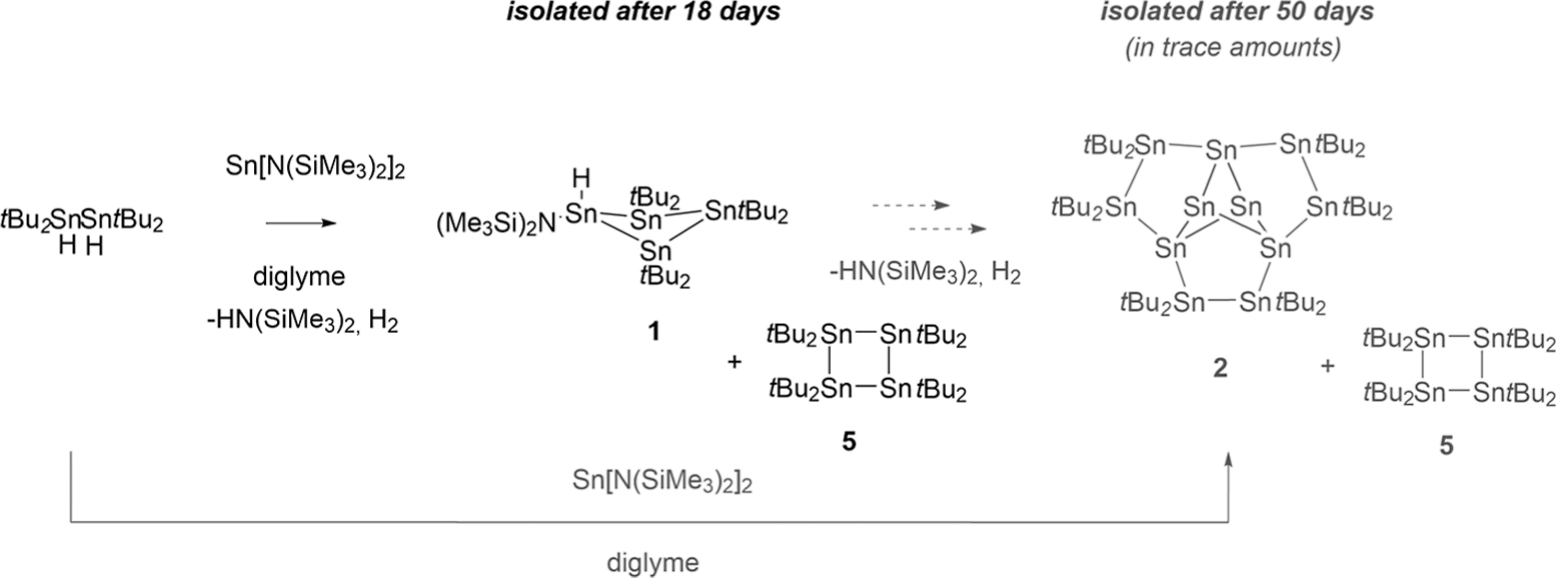
Aminolysis Reaction of *t*Bu_2_HSnSnH*t*Bu_2_ and Stannylene, Sn­[N­(SiMe_3_)_2_]_2_, Yielding Sn_4_ *t*Bu_6_[N­(SiMe_3_)_2_]­(H) (**1**) and Trace Amounts of Higher-Nuclearity Species Sn_11_ *t*Bu_12_ (**2**)

### Spectroscopy and SC-XRD Analysis of 1

The ^119^Sn­{^1^H} NMR spectrum of **1** displays separate resonances, consistent with its three distinct tin sites. Sn2 and Sn3 (cf. Figure 2; *t*Bu substituted tin atoms; δ = 110.4 and 31.2 ppm) appear ∼ 85 ppm downfield from the corresponding signals in *cyclo*-Sn_4_Dep_7_H (δ = 23.8 and 54.8 ppm), reflecting alkyl vs aryl substitution.^48^ The hydride-bearing Sn1 resonates at δ = 199.4 ppm with markedly reduced intensity, in line with diminished NOE buildup reported for the analogous site in *cyclo*-Sn_4_Dep_7_H.^9,48^ Nonetheless, its characteristic Sn–H doublet pattern confirms the assignment. Sn2 and Sn3 signals are broadened by complex ^3^ *J* couplings to *tert*-butyl groups; their satellites are clearly resolved, with symmetric ^119^Sn–^117^Sn coupling but ^119^Sn–^119^Sn deviating from the roof effect, giving an A2BM-type pattern. In the solid-state, **1** adopts a slightly folded tetrastannacyclobutane core (fold angle 154.8°), with Sn–Sn bond lengths, consistent with single bonds (2.818(1)-2.886(1) Å); bonds adjacent to the hydride-bearing Sn1 are marginally shorter. Sn–Sn–Sn angles within the ring are acute (83.43(4)-92.21(4)°), while the environment around Sn1 is strongly distorted from tetrahedral geometry, approaching a pyramidal arrangement. While **1** represents the main product, the reaction also afforded a second species, Sn_11_ *t*Bu_12_ (**2**), obtained only in trace amounts and after prolonged reaction times.

Considering the remaining amide and hydride moieties, **1** represents a likely key intermediate en route toward higher-nuclearity clusters and mirrors the type of reactive species we have already been able to trap in previous studies.^47^ There, isolable cyclic low-nuclearity species evolved into polyhedral cages under prolonged reaction times, supporting a stepwise assembly process initiated by H-/NR_2_-migration—also consistent with proposed amine-elimination pathways in our dipnictene synthesis.^47,49^ Also, in the present case, the concomitant formation of a few needles of Sn_11_ *t*Bu_12_ (**2**) as the second product suggests that further HN­(SiMe_3_)_2_ elimination from **1** furnishes the larger core of **2**.

### Reaction of *t*Bu_2_HSnSnH*t*Bu_2_ with Sn­(NEt_2_)_2_: Isolation of Higher-Nuclearity Clusters Sn_11_ *t*Bu_12_ (2), Sn_15_ *t*Bu_14_ (4), and Sn_16_ *t*Bu_16_ (3)

While Sn­[N­(SiMe_3_)_2_]_2_ proved effective in accessing **1** and trace amounts of **2**, the limited reactivity and slow conversion prompted us to investigate the less shielded, more basic and hence more reactive Sn­(NEt_2_)_2_ as the amide source. In DME, equimolar mixtures with *t*Bu_2_HSnSnH*t*Bu_2_ slowly darkened from yellow to brown over the course of 5 days, again affording small brown needles of Sn_11_ *t*Bu_12_ (**2**, see Scheme 2). Concentration of the supernatant and storage at −30 °C yielded the higher-nuclearity clusters **3** and **4**.

**Scheme 2:**
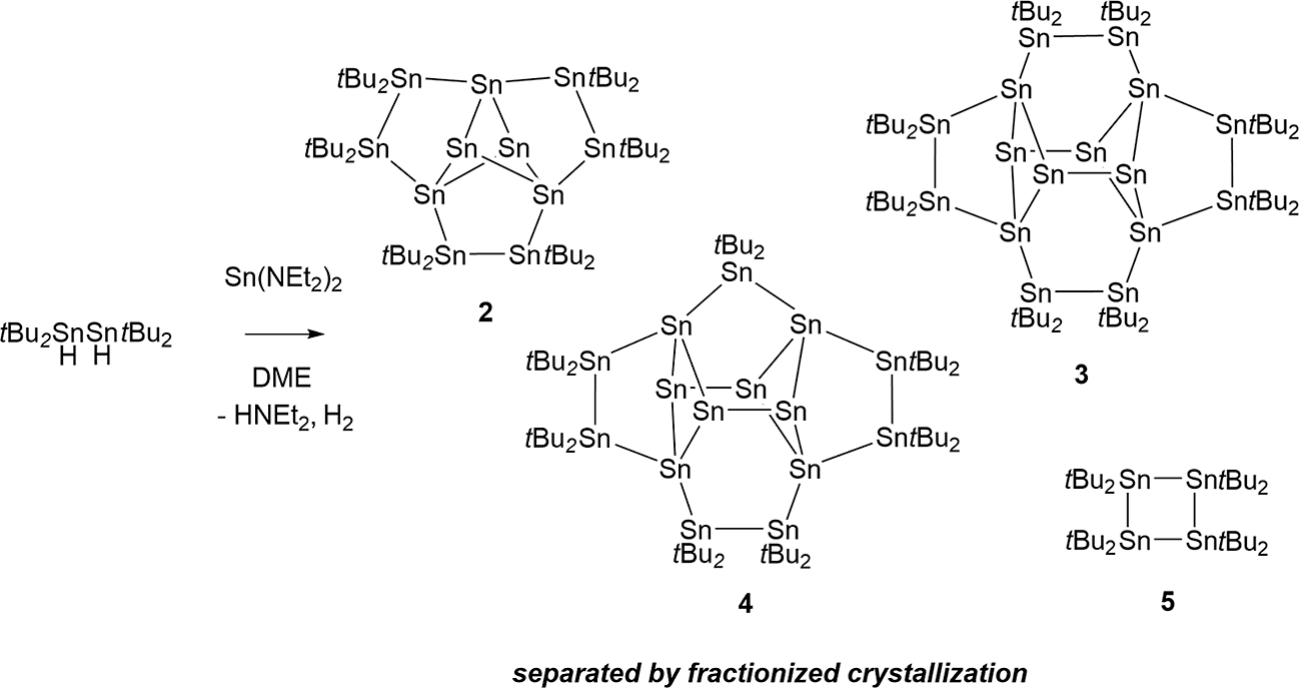
Metalloid *t*Bu-Substituted Tin Clusters Sn_11_ *t*Bu_12_ (**2**), Sn_15_ *t*Bu_14_ (**4**), and Sn_16_ *t*Bu_16_ (**3**) Isolated From Reaction of *t*Bu_2_HSnSnH*t*Bu_2_ With Sn­(NEt_2_)_2_ in DME and Separated by Fractionized Crystallization

The core structure of **2** resembles the tin scaffold of a pentastanna[1.1.1]­propellane (in this case capped by *t*Bu_2_SnSn*t*Bu_2_ fragments), a compound that has recently garnered widespread interest because of its unusual bonding characteristics.^9^ The distannane precursor *t*Bu_2_HSnSnH*t*Bu_2_ serves a dual role in this synthetic protocol: It acts as an internal reducing agent toward the Sn­(II) amide, and thus the resulting *cyclo*-Sn_4_ *t*Bu_8_ (**5**) in all the herein reported reactions is thus not a mere byproduct, but the additional reaction product formed upon reduction of the Sn­(II) precursor, enabling formation of **1** and the clusters **2**, **3**, and **4**. Hence, the isolated yields of **2** increased substantially upon optimization of the hydride to amide ratio from 1:1 to 3:5, highlighting the need for sacrificial amounts of *t*Bu_2_HSnSnH*t*Bu_2_ to drive complete reduction and cluster assembly. Consistent with our mechanistic picture, an initial insertion of the Sn­(II) amide into a Sn–H bond of *t*Bu_2_HSnSnH*t*Bu_2_, thereby eliminating HNR_2_ (vide supra), is followed by dehydrocoupling that concomitantly releases dihydrogen upon cluster formation. Thus, the naked tin atoms in **2**, **3**, and **4** originate from reduction of the Sn­(II) amide precursor. Second, *t*Bu_2_HSnSnH*t*Bu_2_ is incorporated in the clusters and provides sufficient steric shielding as capping groups for the reduced core atoms. While we have not observed *tert*-butyl group migration, the stannylene bridge in **4** and the *t*Bu_6_Sn_3_ unit in **1** originate from the fragmentation of *t*Bu_2_HSnSnH*t*Bu_2_. Control experiments starting from monomeric *t*Bu_2_SnH_2_ with excess Sn­(NEt_2_)_2_ gave only trace amounts of **2**, while no evidence for the formation of smaller clusters with analogous core architectures (i.e., Sn_8_ *t*Bu_6_, capped only by di-*tert*-butyl stannylene bridges) was found.

**Figure 2:**
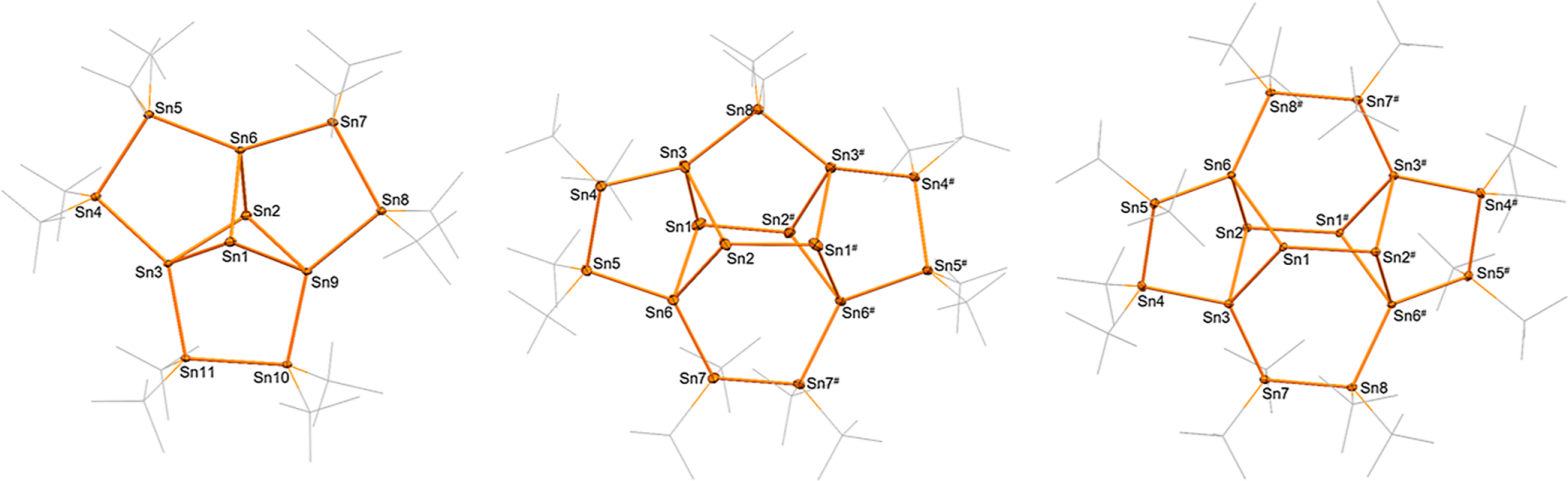
Molecular structures of Sn_11_ *t*Bu_12_ (**2**, left), Sn_15_ *t*Bu_14_ (**4**, middle), and Sn_16_ *t*Bu_16_ (**3**, right). All noncarbon atoms are shown as 30% shaded ellipsoids. Hydrogen atoms are omitted for clarity. Selected bond lengths [Å] for **2** (Sn_apex_ = Sn1, Sn2; Sn_core_ = Sn3, Sn6, Sn9; Sn_subst_ = Sn4, Sn5, Sn7, Sn8, Sn10, Sn11): Sn1···Sn2 3.260(2), Sn_apex_-Sn_core_ 2.811(2)-2.823(2) (avg. 2.816(2)), Sn_core_-Sn_subst_ 2.797(1)-2.807(2) (avg. 2.803(2)), and Sn_subst_-Sn_subst_ 2.908(1)-2.912(1) (avg. 2.910(1)). Selected bond lengths [Å] for **4** (Sn_apex_ = Sn1, Sn1^#^, Sn2, Sn2^#^; Sn_core_ = Sn3, Sn3^#^, Sn6, Sn6^#^; Sn_subst_ = Sn4, Sn4^#^, Sn5, Sn5^#^, Sn7, Sn7^#^, Sn8): Sn1–Sn2 2.863(1), Sn1···Sn2 3.279(1), Sn2···Sn2^#^ 3.0946(9), Sn_apex_-Sn_core_ 2.7634(9)-3.0946(9) (avg. 2.884(9)), Sn_core_-Sn_subst_ 2.803(4)-2.896(4) (avg. 2.833(9)), and Sn_subst_-Sn_subst_ 2.8500(8)-2.896(4) (avg. 2.873(4)). Selected bond lengths [Å] for **3** (Sn_apex_ = Sn1, Sn1^#^, Sn2, Sn2^#^; Sn_core_ = Sn3, Sn3^#^, Sn6, Sn6^#^; Sn_subst_ = Sn4, Sn4^#^, Sn5, Sn5^#^, Sn7, Sn7^#^, Sn8, Sn8^#^): Sn1–Sn2^#^ 2.9016(4), Sn1···Sn2 3.2758(4), Sn1···Sn1^#^ 3.0595(5), Sn_apex_-Sn_core_ 2.8028(4)- 2.8971(4) (avg. 2.8474(5)), Sn_core_-Sn_subst_ 2.8132(4)-2.8538(4) (avg. 2.8276(5)), and Sn_subst_-Sn_subst_ 2.8285(4)-2.8538(4) (avg. 2.8412(4)).

Concentration of the DME reaction mixture, aimed at obtaining a second crop of **2**, instead yielded green needles of Sn_16_ *t*Bu_16_ [**3**, in 16% yield (based on *t*Bu_2_HSnSnH*t*Bu_2_)]. Further volume reduction afforded cubic, brownish crystals of structurally related Sn_15_ *t*Bu_14_ (**4**). Fractional crystallization from DME solutions enabled the clean separation of all three species and highlighted their distinct solubility profiles, whereas from analogues, diglyme reactions only **2** could be obtained. The formation of multiple reaction products is a known characteristic in cluster syntheses and has also been reported by Sita, whose thermolysis of a cyclotristannane afforded not only the pentastanna[1.1.1]­propellane but also a variety of additional tin clusters.^9,48^ In this light, the concomitant isolation of **2**–**4** (see Figure 2) in our system reflects the inherent reactivity of the low-valent tin species, while fractional crystallization allowed their clean separation and individual characterization.

### Spectroscopy of **2**, **3,** and **4**

Due to its extremely low solubility, Sn_11_ *t*Bu_12_ (**2**) could not be characterized by NMR spectroscopy. For Sn_16_ *t*Bu_16_ (**3**), ^1^H and ^13^C NMR spectra revealed two magnetically inequivalent *tert*-butyl substituents, consistent with the observed structure. Clear ^3^ *J*_1H,Sn_ coupling between the *t*Bu protons and tin atoms was observed in proton NMR spectra. However, attempts to record well-resolved ^119^Sn NMR spectra under standard acquisition parameters were unsuccessful. Even extended acquisition times (over 25,000 scans) yielded only broad, low-intensity resonances at 237 and 57 ppm (likely corresponding to alkylated tin atoms). In contrast, Sn_15_ *t*Bu_14_ (**4**) displayed significantly better solubility in benzene, enabling a more detailed NMR spectroscopic study. Seven distinct resonances spanning from 1069 to 70 ppm were observed, reflecting seven magnetically inequivalent tin sites, in agreement with the crystallographically determined structure. The most downfield shifted signal (δ = 1068.7 ppm) is assigned to the stannylene (*t*Bu_2_Sn) bridge, capping two “*naked*” tin atoms, a notably deshielded environment for tetracoordinate tin. Analogous assignments have been made for related tin and silicon clusters [E_6_Ar_6_ (E = Sn,^42^ Si^50^; Ar = Tripp)], where magnetically induced currents in the cluster backbone contribute to such extreme chemical shifts. Remaining resonances were assigned on the basis of their intensity and line shape. UV/vis spectra of Sn_11_ *t*Bu_12_ (**2**), Sn_15_ *t*Bu_14_ (**4**), and Sn_16_ *t*Bu_16_ (**3**) each show a prominent absorption (**2**: 518 nm; **4**: 666 nm; **3**: 633 nm) with additional weaker blue-shifted shoulders. DFT computations (PBE1PBE^51,52^ -GD3 ^53^/x2c-TZVPall^54^//PBE1PBE-GD3/SDD) reproduce these transitions well, with the main absorptions corresponding primarily to HOMO → LUMO or near-HOMO → near-LUMO excitations (cf. Supporting Information).

### Solid-State Structures of **2**, **3,** and **4**

The electronic features are best understood in the context of their solid-state structures. Despite a near-*D*_3*h*_ Sn_11_ skeleton, **2** crystallizes in the low-symmetry space group *P*1, owing to slight triangular distortions. The exclusively tin bound atoms Sn1, Sn3, Sn2, Sn6, and Sn9 (see Figure 2) form a bicyclo[1.1.1] backbone, similar to that observed in [1.1.1]­propellanes (Sn_5_R_6_).^4,9,13,48,55–59^ In contrast to Sn_5_R_6_, bearing aryl substituents at the tetravalent tin atoms, the respective tin atoms in **2** are capped by three *t*Bu_4_Sn_2_ groups instead. In comparison to the distance of the apical tin atoms in previously reported pentastanna[1.1.1]­propellanes, Sn_5_R_6_ (Sn_5_Dep_6_ (Dep = 2,6-Et_2_-C_6_H_3_): 3.3673 Å^48^; Sn_5_Me_2_Ar^Me6^_3_ (Ar^Me6^ = 2,6-(2,4,6-Me_3_-C_6_H_2_)–C_6_H_3_): 3.4878 Å^57^; and Sn_5_Ar^OiPr^_6_ (Ar^OiPr^ = 2,6-(O*i*Pr_2_)-C_6_H_3_): 3.4225(7) Å^13^), **2** displays a short Sn1–Sn2 separation of 3.260(2) Å. Sn–Sn separations of solely tin-bound atoms (2.797(1)-2.823(2) Å) approach those found in α-Sn (2.80 Å),^60^ and the *t*Bu_2_SnSn*t*Bu_2_ caps are essentially coplanar (torsions 0.75(5)-1.71(5)°; cf. 13.9(1)° in Sn_7_Dep_8_^48^). Selected structural parameters are listed in Table 1.

**Table 1:** Selected Structural Parameters of Isolated Structures **2**, **3**, **4,** and α-tin

	Sn^0^–Sn^0^ [Å] (avg.)	Sn^0^–Sn [Å] (avg.)	*t*Bu_2_Sn–Sn*t*Bu_2_ [Å] (avg.)
Sn_11_ *t*Bu_12_ (**2**)	2.817(4)	2.802(7)	2.910(5)
Sn_15_ *t*Bu_14_ (**4**)	2.837(8)	2.811(7)	2.8809(6)
Sn_16_ *t*Bu_16_ (**3**)	2.8655(1)	2.8210(9)	2.8411(9)
α-tin^61,62^	2.80	–	–

In the larger clusters **3** and **4**, eight core Sn atoms form the central scaffold, which is similarly capped by four *t*Bu_4_Sn_2_ bridges in the case of the Sn_16_ cluster **3**, and by three *t*Bu_4_Sn_2_ bridges together with a single *t*Bu_2_Sn linker in the case of the Sn_15_ cluster **4**. In these, average Sn–Sn distances are 2.8672(1) Å in **3** and 2.841(1) Å in **4**, with the shortest separations [down to 2.7634(9) Å in **4**] essentially matching the bulk metal (2.80 Å,^61^ see Table 1). Despite the slight compression within the core, the local tetrahedral environment around the formally neutral tin atoms is retained. In **4**, one distannadiyl bridge is replaced by a stannylene unit (cf. Figure 2; Sn8), resulting in a slightly wider Sn–Sn–Sn angle (102.94(4)°) and marginally elongated Sn–Sn bonds, indicative of geometric strain accompanying the loss of a bridging unit. Alternatively, the cores of **3** and **4** can thus be rationalized as two fused [1.1.1]­propellane-type fragments in which one bridging stannylene unit is successively removed; the resulting apical tin atoms (Sn1, Sn1^#^, Sn2, Sn2^#^) define four formally neutral atoms, which anchor the fused propellane framework. Similar to the distance between the apical tin atoms in **2**, the Sn1–Sn2 separations in **3** and **4** amount to 3.2858(4) and 3.279(1) Å, respectively. In contrast to previously reported larger tin clusters—Sn_17_[GaCl­(dpp)]_4_^16^ or Sn_20_(Si*t*Bu_3_)_10_Cl_2_^38^—which are built from interconnected Sn_9_ or Sn_6_ and Sn_8_ fragments, respectively, **3** and **4** feature a Sn_8_ cluster core, which is structurally closely related to that of α-Sn.^61,62^ Bader and NBO analyses support this structural interpretation, showing no bond critical points (BCPs) between the apical tin atoms, consistent with their role as vertices of fused propellanes (see Section 2.5).

### Computational Analyses of Sn_11_ *t*Bu_12_ (**2**), Sn_15_ *t*Bu_14_ (**4**), and Sn_16_ *t*Bu_16_ (**3**)

All three clusters share a characteristic architecture comprising a compact core of “*naked*” tin atoms that are either tri- or tetracoordinate, flanked by *t*Bu_2_SnSn*t*Bu_2_ or *t*Bu_2_Sn bridging units (vide supra).

Molecular orbital analysis shows that the cluster cores are constructed from a defined set of frontier MOs with pronounced multicenter character distributed over multiple Sn atoms (cf. Figures S26–S33 in the Supporting Information). In contrast, orbitals associated with the *t*Bu-bearing ditin or stannylene bridges are largely localized on those substituent units and are energetically and spatially decoupled from the core manifold. In all three clusters, frontier MOs (and near-degenerate neighbors) are core-dominated, with calculated transitions matching the observed absorptions (see UV/vis). Remarkably, all three compounds exhibit sets of deeper-lying orbitals with shapes closely resembling atomic *s*-, *p*-, and even *d*-/*f*-type functions (a selection of MOs is shown in the SI), a feature pointing toward the superatom concept,^63–66^ as previously invoked for different main-group or transition-metal-based clusters (e.g., Au_20_(*t*Bu_3_P)_8_,^67^ Al_50_Cp*_12_,^68^ Ga_23_[N­(SiMe_3_)_2_]_11_,^69^ SiAl_14_(Cp*)_6_,^70^ etc.). Such orbital motifs underscore the extent of electronic delocalization and suggest that these aggregates may be rationalized as “*superatomic*” units,^63–66^ also feasible as molecular models for the metal bulk, rather than mere collections of metal–metal bonds. All three compounds, **2**, **3** and **4**, optimize to closed-shell singlets with large singlet–triplet gaps (e.g., **2**: ΔEST_vert_ = 103.9 kJ*mol^–1^; ΔEST_adiab_ = 109.5 kJ*mol^–1^). Furthermore, the singlet wave functions show no significant spin contamination. This ground-state assignment aligns with closed-shell descriptions reported for structurally related heavier element main-group systems E_5_R_6_ and E_6_R_6_ (E = Si, Ge, Sn).^3,50,56,71^

No localized lone pairs are found at the 3-fold coordinated apex tin atoms in complementary NBO analysis, consistent with the delocalization observed in the canonical orbitals. Instead, donation from Sn–Sn bonding orbitals into adjacent Sn–Sn antibonding orbitals is evident, with second-order stabilization energy contributions reaching up to ca. 25 kcal*mol^–1^. QTAIM analysis^72,73^ of the electron density determines no BCPs between the apical tin atoms in **2** (Sn1, Sn2), **3** (Sn1, Sn1^#^, Sn2, Sn2^#^), and **4** (Sn1, Sn1^#^, Sn2, Sn2^#^). A contour plot of the Laplacian (∇^2^ρ) of **3** and AIM charges at apical tin atoms is shown in Figure 3. The absence of direct interactions, together with the multicenter bonding manifold in the core, is in agreement with an assignment of these sites as formally neutral (Sn^0^) centers embedded in the multinuclear core for **3**. The respective results for **2** and **4** are available in the Supporting Information. Both natural population analysis (NBO/NPA) and AIM charges derived from Bader analysis yield values close to zero at the apical atoms in **2**, **3,** and **4**, whereas the substituent-bearing tin sites show somewhat larger (though still modest) deviations from neutrality (cf. SI).

**Figure 3:**
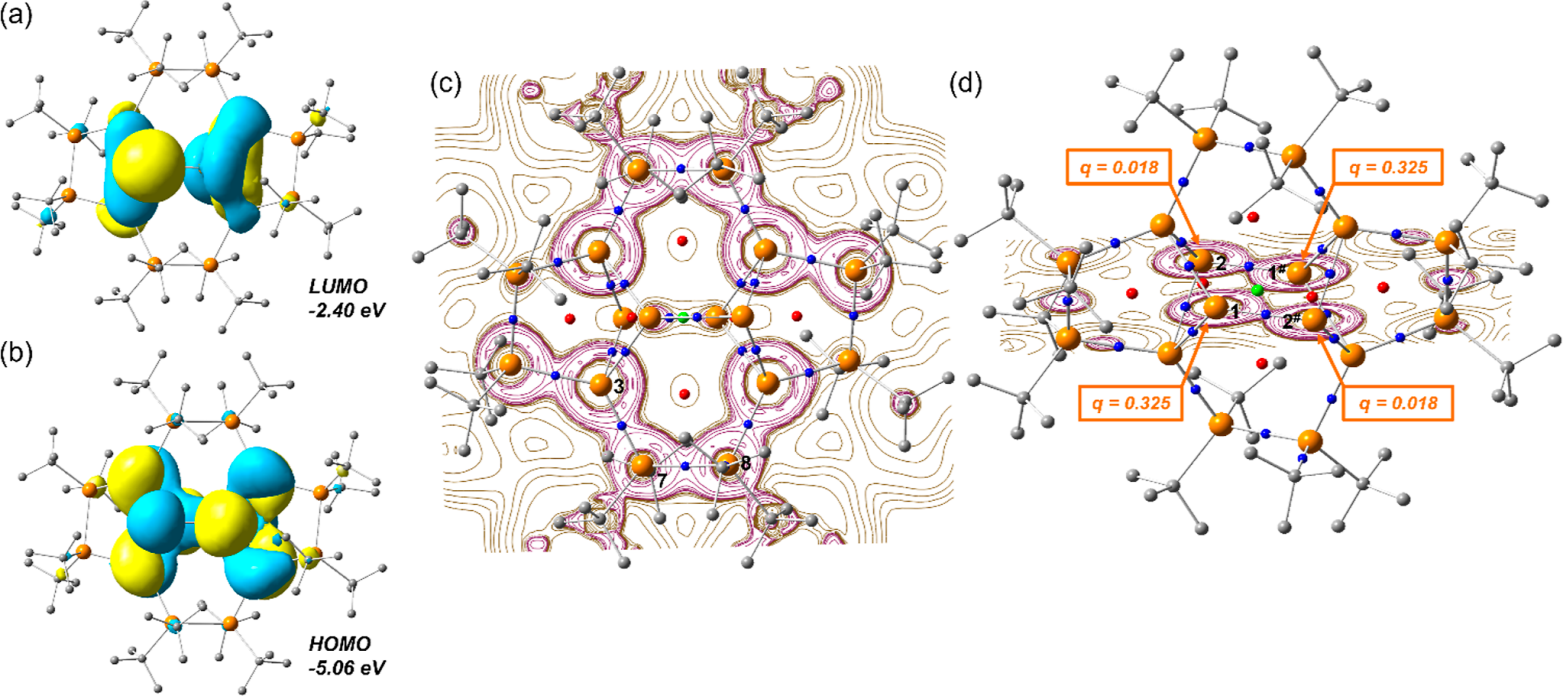
Selected DFT and QTAIM results (PBE1PBE^51,52^ -GD3 ^53^/x2c-TZVPall^54^//PBE1PBE-GD3/SDD) for Sn_16_ *t*Bu_16_ (**3**). (a) LUMO and (b) HOMO of **3**; (c) contour plot of the Laplacian (∇^2^ρ) in the top view (Sn3–Sn7–Sn8 plane); (d) contour plot of the Laplacian (∇^2^ρ) in the side view (Sn2–Sn1–Sn2^#^ plane) including AIM charges (q) for apical tin atoms. Critical points (CPs) are color-coded: bond critical points (BCPs): blue; ring critical points: red; cage critical points: green.

### ^119^Sn Mössbauer Spectroscopy of Sn_11_ *t*Bu_12_ (**2**)

The ^119^Sn Mössbauer spectrum of **2** shows signals at δ = 1.827(4) (***A***), 1.810(5) (***B***), and 1.76(1) mm*s^–1^ (***C***) in a 2:3:6 intensity ratio. Signal ***A*** (Δ*E*_*Q*_ = 1.02(1) mm*s^–1^) is attributed to the apical tin atoms Sn1 and Sn2 (Figure 3), while signal ***C*** (Δ*E*_*Q*_ = 1.90(2) mm*s^–1^) corresponds to the six *t*Bu-substituted tin atoms of **2**. Consistent with the all-tin tetrahedral coordination and formally near-zero charges suggested by QTAIM analysis, Sn3/Sn6/Sn9 exhibit a small quadrupole splitting (Δ*E*_*Q*_ = 0.404(6) mm*s^–1^) and an isomer shift of 1.810(5) mm*s^–1^, with values approaching the properties of α-tin (δ = 2.02 mm*s^–1^). Similar values were previously reported for the neutral centaur polyhedron Sn_10_[Si­(SiMe_3_)_3_]_6_.^74,75^ For experimental details and fitting parameters, see Supporting Information.

### Reaction of *t*Bu_2_HSnSnH*t*Bu_2_ with Pb­[N­(SiMe_3_)_2_]_2_: Isolation of Heterobimetallic *Spiro* Compound **6**

Building on the reactivity trends discussed above, we extended our synthetic protocol to heterobimetallic Sn/Pb systems. Treatment of *t*Bu_2_HSnSnH*t*Bu_2_ with equimolar amounts of the heavier Lappert’s plumbylene, Pb­[N­(SiMe_3_)_2_]_2_, yielded the unprecedented *spiro*-type framework, *t*Bu_6_Sn_3_PbSn_3_ *t*Bu_6_ (**6**; ca. 40 days), in which a central lead atom is embedded within an otherwise tin-based skeleton. Mechanistically, the formation process closely parallels the pathway to tetrastannacyclobutane intermediate **1**: In both cases, H-migration from the dihydride source to the Lappert-type amide initiates Sn/Pb bond formation. Whereas **1** represents a trapped tin-based intermediate en route to larger homometallic aggregates, the present system incorporates the lead center from Pb­(II) amide directly. This link between **1** and **6** highlights a common assembly principle while expanding the accessible structural diversity by integrating a second element into the polyhedral scaffold. The formation of the *spiro*-type framework proceeds analogously to the tin-based system, with the distannane precursor again acting as an internal reducing agent toward the group 14 amide. Concomitant formation of **5** as the second reaction product confirms the reduction of the Pb­(II) precursor, consistent with the prior reactivity trends (see Scheme 3).

**Scheme 3:**
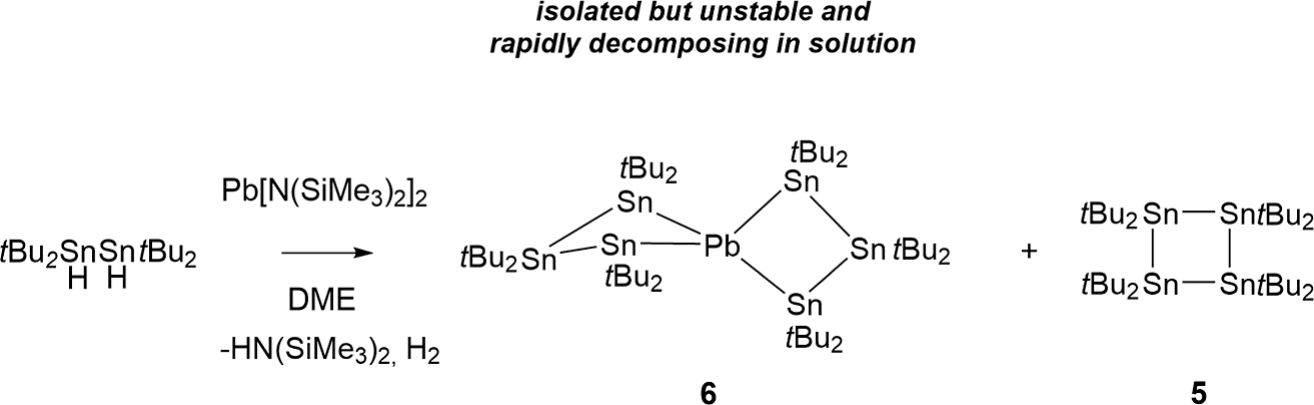
Reaction of *t*Bu_2_HSnSnH*t*Bu_2_ and Lappert’s Plumbylene, Affording the Spiro-Type Compound **6**

The UV/vis spectrum of **6** displays a dominant broad absorption band centered at 365 nm, accompanied by a weaker shoulder extending from ca. 490 nm and gradually tailing toward 650 nm—accounting for the dark red color of the compound. Similar low-energy tailing behavior has previously been described for silicon clusters and was attributed to a number of FMOs with small energy separations, resulting in overlapping electronic transitions within a narrow energy window.^76,77^ TD-DFT calculations (PBE1PBE^51,52^ -GD3 ^53^/x2c-TZVPall^54^) reproduce the principal absorption at 373 nm as an intense HOMO → LUMO excitation, while several additional, closely spaced near-HOMO → near-LUMO transitions of lower intensity occur in the same spectral region. ^119^Sn HMBC NMR spectra show two distinct resonances at δ = 133.6 ppm (PbSn*t*Bu_2_) and 85.7 ppm (SnSn*t*Bu_2_), which are slightly more downfield shifted compared to the alkylated tin atoms in **1**. Attempts to acquire reliable ^207^Pb NMR spectroscopic data were precluded by the pronounced instability of **6** in solution; redissolved crystals decompose within ca. 30 min, accompanied by precipitation of Pb^0^. The solid-state structure of **6** features a Pb center tetrahedrally coordinated by three tin atoms. In analogy to **1**, the Sn_3_Pb rings in **6**, are slightly folded (fold angle = 115.5° and 155.7° respectively), with Sn–Pb separations of 2.9706(8)-3.0013(8) Å—marginally elongated compared to Ph_3_SnPbPh_3_ (2.828(7) Å)^78^—while the adjacent Sn–Sn bonds (2.8550(7)-2.8860(8) Å) remain in the typical single-bond range (see Figure 4).

**Figure 4:**
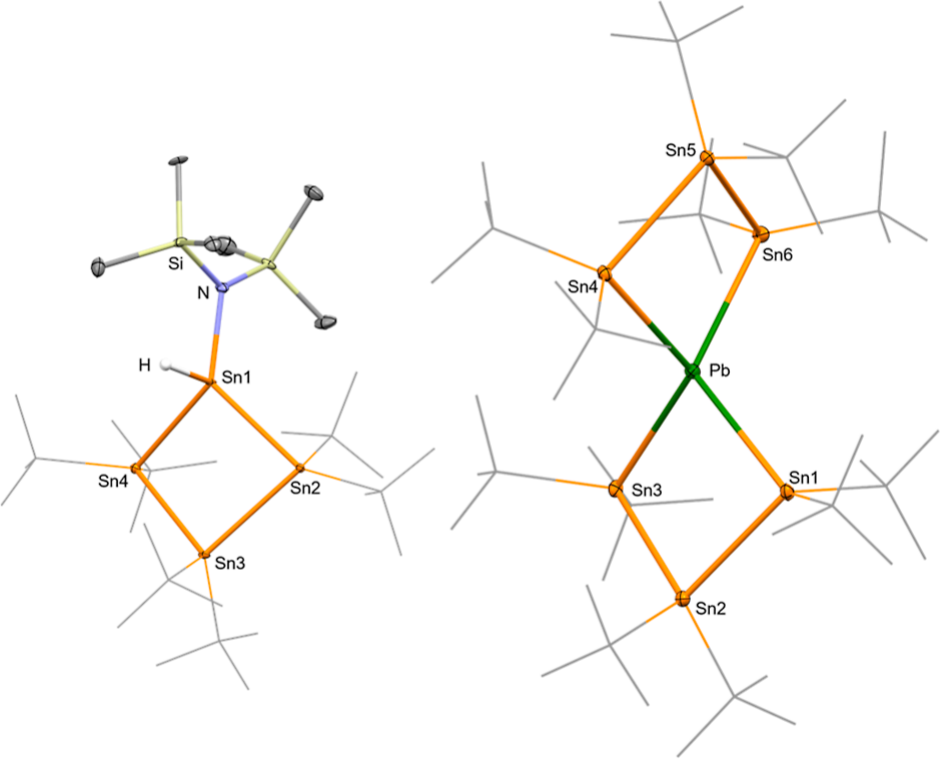
Molecular structures of **1** (left) and **6** (right). All *t*Bu carbons are shown as wireframe; all other atoms are shown as 30% shaded ellipsoids. Hydrogen atoms (except SnH) are omitted for clarity. Selected bond lengths [Å] for **1**: Sn–Sn 2.818(1)-2.886(1) (avg. 2.854(1)), Sn1–H 1.68(4), and Sn1–N 2.12(1). Selected bond lengths [Å] for **6**: Pb–Sn 2.9706(8)-3.0013(8) (avg. 2.9821(8)) and Sn–Sn 2.8527(7)-2.8829(8) (avg. 2.8637(8)).

## Conclusion

A modular hydrostannolysis route, which couples readily available Sn­(II) amides with organotin hydrides, delivers metalloid tin clusters under mild conditions. The protocol tolerates the use of relatively compact *tert*-butyl ligands, which in turn granted access to genuinely new core motifs, thereby overcoming the ligand-imposed structural limitations of established methodologies. A tetrastannacyclobutane, Sn_4_ *t*Bu_6_(N­(SiMe_3_)_2_)­(H), is isolated as an intermediate en route toward the higher-nuclearity species Sn_11_ *t*Bu_12_, Sn_16_ *t*Bu_16_ and Sn_15_ *t*Bu_14_. Structurally, all three clusters share a characteristic architecture comprising a compact core of “*naked*” tin atoms that are either tri- or tetracoordinate, flanked by *t*Bu_2_SnSn*t*Bu_2_ or *t*Bu_2_Sn bridging units. Sn_11_ *t*Bu_12_ is closely related to the structure of pentastanna[1.1.1]­propellane, and the larger clusters share α-Sn-like architectures with six-membered rings in chair arrangements closely related to gray tin and Sn–Sn distances down to 2.763 Å (α-Sn ≈ 2.80 Å). ^119^Sn Mössbauer spectroscopy of Sn_11_ *t*Bu_12_ confirms net-near zero charges at the apical and tetrahedral all-tin coordinated core atoms, a feature that is supported by QTAIM and NBO analysis for all three clusters. DFT computations furthermore indicate extensive delocalization over the whole cluster backbone, resulting in superatom-like molecular orbitals.

## Experimental Section

All manipulations involving air- or moisture-sensitive compounds were either performed under a nitrogen atmosphere using standard Schlenk tube techniques or were carried out in a nitrogen-flushed Glovebox UNILAB supplied by M.Braun. Anhydrous and deoxygenated solvents were obtained from an Innovative Technology solvent drying system. Compounds *t*Bu_2_ClSnSnCl*t*Bu_2_, *t*Bu_2_SnCl_2_, *t*Bu_2_HSnSnH*t*Bu_2_, *t*Bu_2_SnH_2_, Sn­[N­(SiMe_3_)_2_]_2_, and Pb­[N­(SiMe_3_)_2_]_2_ were prepared according to literature procedures.^79–82^ Deuterated solvents for NMR spectroscopic measurements were degassed using the “*freeze*–*pump*–*thaw*” method and stored over 3 Å molecular sieves. All other chemicals from commercial sources were used as purchased from chemical suppliers. Melting points were determined by 3-fold determination with an electrothermal Mel-Temp instrument.

### NMR Spectroscopy

^1^H (300.22 MHz), ^13^C (75.5 MHz), ^29^Si (59.64 MHz), as well as ^119^Sn (111.92 MHz), (94.8 MHz) NMR spectra of precursors and compounds **1**, **2**, **3,** and **4** were recorded on a Varian Mercury 300 MHz spectrometer from Varian at 25 °C, if not otherwise stated. ^1^H (400.13 MHz), ^29^Si (79.50 MHz), and ^119^Sn (149.21 MHz) NMR spectra, as well as all 2D experiments for compound **6,** were recorded on a Pulse fourth generation RS2D 400 MHz spectrometer, with included Cameleon4, from RS2D at 25 °C. Spectra were referenced to solvent residual signals. Chemical shifts are given in ppm relative to TMS regarding ^1^H, ^13^C, and ^29^Si. ^119^Sn resonances are given relative to Me_4_Sn. Coupling constants (^n^ *J*) are reported in Hertz (Hz).

### UV/Vis, ATR-FTIR, and Raman Spectroscopy

All UV–vis measurements were performed in quartz glass cuvettes with a thickness of 1 cm on a Cary 60 UV–vis device from Agilent Technologies. If not otherwise stated, measurements were done in absorption mode. Absorption maxima are given in nm. Extinction coefficients (ε) are given in Lcm^–1^ mol^–1^. All IR measurements were measured fast under ambient conditions on an ALPHA-P device from Bruker in transmission mode. The letters s (strong), m (medium), and w (weak) are used to indicate the intensity of the transmission bands. All Raman measurements were performed in a capillary using a PerkinElmer Raman Station 400F with a built-in 350 mW laser operating at 785 nm.

### ^119^Sn Mössbauer Spectroscopy

A ^119^Sn Mössbauer spectroscopic investigation of sample **2** utilized a Ca^119m^SnO_3_ source with an activity of 5 mCi. The sample was placed in a PMMA container, the thickness of which was optimized according to Long et al.^83^ A palladium foil of 0.05 mm thickness was used to reduce the tin K X-rays concurrently emitted by this source. The measurement was performed in a continuous flow cryostat system (Janis Research Co, LLC) at 6 K. The source was kept at room temperature. Fitting of the spectrum was performed with the WinNormos for the Igor6 software package.^84^ The counting time was around 1 day.

### Single Crystal X-Ray Diffraction

For single crystal X-ray diffractometry of compounds **1**, **2**, **3**, **4,** and **6,** suitable crystals were covered with a layer of silicone oil. A single crystal was selected, mounted on a glass rod on a copper pin, and placed in the cold N_2_ stream provided by an Oxford Cryosystems cryometer (T = 100 K), if not otherwise stated. XRD data collection was performed on a Bruker APEX II diffractometer with the use of Mo Kα radiation (λ = 0.71073 Å) from an IμS microsource and a CCD area detector. Empirical absorption corrections were applied using SADABS.^85,86^

For single crystal X-ray diffractometry of compound **5**, suitable crystals were selected and covered with a layer of silicone oil. A single crystal was picked and subsequently mounted on a glass rod on a copper pin and placed in a cold N_2_ stream (*T* = 100 K, Oxford 800 Cryometer). XRD data collection was performed on a Rigaku XtaLAB Synergy-S, Dualflex, HyPix-Arc 100 diffractometer with the use of Cu Kα radiation (λ = 1.54184 Å). The diffraction pattern was indexed, and the total number of runs and images was based on the strategy calculation from the program CrysAlisPro.^87^ The structures were solved with the use of either direct methods or the Patterson option in SHELXS. Structure refinement was carried out using SHELXL.^88,89^ CIF files were edited, validated, and formatted with the program OLEX2.^90^ The space group assignments and structural solutions were evaluated using PLATON.^91,92^ All non-hydrogen atoms were refined anisotropically. Hydrogen atoms next to the heavy atom Sn were located on the difference Fourier map in the solid-state structure of **1**. All other hydrogen atoms were placed in calculated positions corresponding to standard bond lengths and angles using riding models. Tables S2–S4 contain crystallographic data and details for measurements and refinement.

### Quantum Chemical Investigations

All calculations were carried out using the Gaussian16 program package.^93^ For geometry optimizations and the subsequent calculation of vibrational frequencies, the PBE1PBE hybrid functional,^51,52,94^ together with a Stuttgart–Dresden pseudopotential on tin/lead^95^ and D95 all-electron basis sets^96,97,98,99,100,101^ on the remaining elements, and GD3 ^53^ Grimme dispersion correction were used. For UV/vis and QTAIM calculations, the PBE1PBE hybrid functional,^51,52,94^ GD3 ^53^ Grimme dispersion correction, and x2c-TZVPall^54^ basis set were used. An UltraFine integration grid (a 99,590 grid) was used throughout. The Multiwfn^73^ program package was used for the calculation of all QTAIM results. QTAIM results were visualized by using GaussView.

### Synthesis

#### Sn_4_ *t*Bu_6_(H)­[N­(Si­(CH_3_)_3_)_2_] (**1**)

A solution of 234 mg of *t*Bu_2_HSnSnH*t*Bu_2_ (0.5 mmol, 3.0 equiv) in 4 mL of diglyme was added dropwise to 366 mg of Sn­[N­(SiMe_3_)_2_]_2_ (0.83 mmol, 5.0 equiv), which was dissolved in 4 mL of diglyme. The solution turned slowly orange. After 4 days, the solution appeared red and turned brown after overall 7 days. After 18 days, the product crystallized from the solution as yellowish, nearly colorless crystals, which turned out to be suitable for single crystal X-ray diffraction. The supernatant solution was concentrated and stored at −30 °C to give a second crop.

Yield: 160 mg (49% referred to *t*Bu_2_HSnSnH*t*Bu_2_), yellow powder. m.p.^exp^ 100–110 °C (decomposition).

^1^H NMR (300.22 MHz, C_6_D_6_) δ 6.95 (s, ^1^ *J*_H,117Sn_ = 1001 Hz, ^1^ *J*_H,119Sn_ = 1047 Hz, ^2^ *J*_H,Sn_ = 38 Hz, ^3^ *J*_H,Sn_ = 49 Hz, 2 H; Sn­(*H*)­[N­(Si­(CH_3_)_3_)_2_]), 1.61 (s, 18 H; Sn­(H)­[N­(Si­(CH_3_)_3_)_2_]­Sn*tBu*_2_Sn*t*Bu_2_), 1.59 (s, 18 H; Sn­(H)­[N­(Si­(CH_3_)_3_)_2_]­Sn*tBu*_2_Sn*t*Bu_2_), 1.57 (s, 9 H; Sn­(H)­[N­(Si­(CH_3_)_3_)_2_]­Sn*t*Bu_2_Sn*tBu*_2_), 1.52 (s, 9 H; Sn­(H)­[N­(Si­(CH_3_)_3_)_2_]­Sn*t*Bu_2_Sn*tBu*_2_), 0.43 (s, 18 H; SnH­[N­(Si­(C*H*_3_)_3_)_2_]) ppm. ^13^C NMR (75.5 MHz, C_6_D_6_) δ 38.89 (*C*(CH_3_)_3_), 38.08 (*C*(CH_3_)_3_), 35.28 (C­(*C*H_3_)_3_), 34.91 (C­(*C*H_3_)_3_), 34.68 (C­(*C*H_3_)_3_), 34.51 (*C*(CH_3_)_3_), 34.28 (C­(*C*H_3_)_3_), 33.44 (*C*(CH_3_)_3_), 5.89 (SnH­[N­(Si­(*C*H_3_)_3_)_2_]) ppm. ^119^Sn NMR (111.92 MHz, C_6_D_6_) δ 110.39 (^1^ *J*_Sn,117Sn_ = 1197 Hz, ^1^ *J*_Sn,119Sn_ = 1244 Hz, ^1^ *J*_Sn,117Sn_ = 1080 Hz, ^1^ *J*_Sn,119Sn_ = 1130 Hz, ^2^ *J*_Sn,117Sn_ = 1162 Hz; Sn­(H)­[N­(Si­(CH_3_)_3_)_2_]­Sn*t*Bu_2_Sn*t*Bu_2_), 31.23 (^1^ *J*_Sn,117Sn_ = 1080 Hz, ^1^ *J*_Sn,119Sn_ = 1130 Hz; Sn­(H)­[N­(Si­(CH_3_)_3_)_2_]­Sn*t*Bu_2_Sn*t*Bu_2_), −199.43 (d, ^1^ *J*_Sn,H_ = 1048 Hz; SnH­[N­(Si­(CH_3_)_3_)_2_]) ppm. ATR-FTIR υ̃ 2960 (m), 2835 (m), 2704 (w), 1743 (m; ν_s_ SnH), 1588 (w), 1462 (m), 1359 (m), 1258 (m), 1144 (m), 1011 (s), 797 (s), 622 (m), 525 (m), 383 (m) cm^–1^.

### Sn_11_ *t*Bu_12_ (**2**)

A solution of 468 mg of *t*Bu_2_HSnSnH*t*Bu_2_ (1.0 mmol, 3.0 equiv) in 8 mL of diglyme was added dropwise to 438 mg of Sn­(NEt_2_)_2_ (1.67 mmol, 5.0 equiv) dissolved in 5 mL of diglyme. The solution turned orange, and then the color slowly faded to dark brown within a few minutes. After 5 days, the product crystallized from the dark brown solution as brownish orange small crystals, which were dried *in vacuo*. The supernatant solution was concentrated to give a second crop. Crystals suitable for X-ray crystallography were obtained from more diluted reactions in THF/benzene.

Yield: 264 mg (40% referred to *t*Bu_2_HSnSnH*t*Bu_2_ and Sn­(NEt_2_)_2_), dark orange needles. m.p.^exp^ > 210 °C (decomposition). Anal. Calcd for C_48_H_108_Sn_11_: C, 28.95; H, 5.47. Found: C, 27.26; H, 4.71.

Yield for *t*Bu_2_HSnSnH*t*Bu_2_/Sn­(NEt_2_)_2_ = 1:1 in diglyme: 105 mg (16% referred to *t*Bu_2_HSnSnH*t*Bu_2_).

Raman (140 mW, 10 scans) 184 (63), 499 (62), 803 (40), 1148 (70), 1525 (100, broad), 1864 (75, broad) cm^–1^. UV–vis (cyclooctane) λ_max_ (ε, L cm^–1^ mol^–1^) 518 (−), 315 (−) nm.

### Reaction of *t*Bu_2_HSnSnH*t*Bu_2_ with Sn­(NEt_2_)_2_ – Sn_15_ *t*Bu_14_ (**4**) and Sn_16_ *t*Bu_16_ (**3**)

A solution of 468 mg of *t*Bu_2_HSnSnH*t*Bu_2_ (1.0 mmol, 3.0 equiv) in 8 mL of DME was added dropwise to 470 mg of Sn­(NEt_2_)_2_ (1.79 mmol, 5.3 equiv), which was also dissolved in 5 mL of DME. The solution turned orange, and then the color slowly faded to dark brown. After 5 days, Sn_11_ *t*Bu_12_ (**2**) crystallized from the dark brown solution as small, brownish-orange crystals, which were dried *in vacuo*. The supernatant solution was concentrated to give green needles of Sn_16_ *t*Bu_16_ (**3**) suitable for X-ray crystallography. Evaporation of more solvents gave big cubic, brownish-purple crystals of Sn_15_ *t*Bu_14_ (**4**) also suitable for single crystal X-ray diffraction after an overall 10 days.

Analytical data for Sn_11_ *t*Bu_12_ (**2**) are identical to data for **2** obtained from diglyme solutions.

Yield: 102 mg (15% referred to *t*Bu_2_HSnSnH*t*Bu_2_ and Sn­(NEt_2_)_2_), brownish-orange needles.

Analytical data for Sn_16_ *t*Bu_16_ (**3**):

Yield: 109 mg (16% referred to *t*Bu_2_HSnSnH*t*Bu_2_), green needles. m.p.^exp^ > 190 °C (decomposition).

Anal. Calcd for C_64_H_144_Sn_16_*C_8_H_20_O_4_: C, 28.89; H, 5.52. Found: C, 28.05; H, 5.09.

^1^H NMR (300.22 MHz, C_6_D_6_) δ 1.67 (s, 72 H; 2x*tBu*_2_SnSn*tBu*_2_), 1.63 (s, 72 H; 2x*tBu*_2_SnSn*tBu*_2_) ppm. ^1^H NMR (300.22 MHz, *d*_8_-THF) δ 1.51 (s, ^3^ *J*_H,Sn_ = 66 Hz, 72 H; 2x*tBu*_2_SnSn*tBu*_2_), 1.54 (s, ^3^ *J*_H,Sn_ = 67 Hz, 72 H; 2x*tBu*_2_SnSn*tBu*_2_) ppm. ^13^C NMR (75.5 MHz, *d*_8_-THF) δ 55.48 (*C*(CH_3_)_3_), 44.34 (*C*(CH_3_)_3_), 35.55 (C­(*C*H_3_)_3_), 35.12 (C­(*C*H_3_)_3_) ppm. UV–vis (THF) λ_max_ (ε, Lcm^–1^ mol^–1^) 633 (1404), 390 (−), 310 (−) nm.

Analytical data for Sn_15_ *t*Bu_14_ (**4**):

Yield: 77 mg (10% referred to *t*Bu_2_HSnSnH*t*Bu_2_), brownish purple blocks. m.p.^exp^ > 178 °C (decomposition).

Anal. Calcd for C_63_H_132_Sn_15_: C, 28.34; H, 4.98. Found: C, 27.78; H, 4.82.

^1^H NMR (300.22 MHz, C_6_D_6_) δ 1.68 (s, ^3^ *J*_H,Sn_ = 66 Hz, 36 H; *tBu*_2_SnSn*tBu*_2_), 1.63 (s, ^3^J_H,Sn_ = 72 Hz, 18 H; *tBu*_2_Sn),1.58 (s, ^3^ *J*_H,Sn_ = 66 Hz, 36 H; 2x*tBu*_2_SnSn*t*Bu_2_),1.57 (s, ^3^ *J*_H,Sn_ = 68 Hz, 36 H; 2x*tBu*_2_SnSn*t*Bu_2_) ppm.

^13^C NMR (75.5 MHz, C_6_D_6_) δ 37.06 (*C*(CH_3_)_3_), 36.65 (*C*(CH_3_)_3_), 36.48 (*C*(CH_3_)_3_), 35.54 (*C*(CH_3_)_3_), 34.93 (C­(*C*H_3_)_3_), 34.75 (C­(*C*H_3_)_3_), 34.49 (C­(*C*H_3_)_3_), 34.01 (C­(*C*H_3_)_3_) ppm. ^119^Sn NMR (111.92 MHz, C_6_D_6_) δ 1068.7 (*t*Bu_2_Sn­(Sn)_2_), 369.2 (2x*t*Bu_2_Sn­(Sn)­Sn*t*Bu_2_), 342.14 (2x*t*Bu_2_Sn­(Sn)­Sn*t*Bu_2_), 176.3 (2xSn­(*t*Bu_2_Sn)­(Sn*t*Bu_2_)), 109.2 (4xSn), 99.2 (*t*Bu_2_SnSn*t*Bu_2_), 70.6 (2xSn­(*t*Bu_2_Sn)­(Sn*t*Bu_2_)) ppm. UV–vis (benzene) λ_max_ (ε, Lcm^–1^ mol^–1^) 666 (705), 550 (798), 438 (−), 377 (−), 290 (−) nm.

### *t*Bu_6_Sn_3_PbSn_3_ *t*Bu_6_ (**6**)

A solution of 121 mg of Pb­[N­(SiMe_3_)_2_]_2_ (0.23 mmol, 1.0 equiv) in DME was added dropwise to a solution of 111 mg of *t*Bu_2_HSnSnH*t*Bu_2_ (0.24 mmol, 1.0 equiv) also dissolved in DME at −30 °C. Upon addition, the solution turned dark yellow, which slowly faded to dark red. After 40 days, the product crystallized at −30 °C as dark red plates suitable for SC-XRD analysis.

Yield: 24 mg (19% referred to *t*Bu_2_HSnSnH*t*Bu_2_), red plates. m.p.^exp^ > 143 °C (decomposition).

^1^H NMR (400.13 MHz, C_6_D_6_) δ 1.69 (s, 72 H; 8x C­(C*H*_3_)_3_), 1.62 (s, 36 H; 4x C­(C*H*_3_)_3_) ppm. ^119^Sn NMR (149.21 MHz, C_6_D_6_, 2D-^1^H,^119^Sn-HMBC) δ 133.6 (PbSn­(C­(CH_3_)_3_)_2_), 85.7 (SnSn­(C­(CH_3_)_3_)_2_) ppm. UV–vis (benzene) λ_max_ (ε, Lcm^–1^ mol^–1^) 365 (−), 490 (−) nm.

## Supplementary Material



## Footnotes

∥ Preliminary, unpublished results show the deamination protocol is also compatible with a range of aryl ligands, spanning moderate to high steric demand, enabling reproduction of known cluster motifs (i.e., Sn_6_Tripp_6_, Sn_10_Tripp_8_, etc.). These findings will be detailed in forthcoming reports.

## Notes

Additional references cited in the Supporting Information (PDF)
