# Supplementary material for: Metalloid Tin Clusters, Sn n tBum, with α‑Sn-Like Structural Motifs
Source: Inorg Chem. 2026 Jun 25;65(27):15723–33. doi: 10.1021/acs.inorgchem.6c01824 (PMC13370870; doi:10.1021/acs.inorgchem.6c01824)
Supplement: Supplementary file 1 [file ic6c01824_si_001.pdf]

Supporting Experimental Information

Metalloid Tin Clusters, $Sn_n tBu_m$, with α-Sn Like Structural Motifs

Beate G. Steller‡,[a], Clara A. Roller‡,[a], Steffen Klenner[b], Joshua Wiethölter[b], Rainer Pöttgen[b], Michaela Flock[a], and Roland C. Fischer[a],*

[a] Institute of Inorganic Chemistry, Graz University of Technology, Stremayrgasse 9/V, 8010 Graz, AUSTRIA
[b] Institut für Anorganische und Analytische Chemie, Universität Münster, Corrensstraße 30, 48149 Münster, GERMANY

* Email: roland.fischer@tugraz.at

Table of Contents

1 Experimental Details 2
2 NMR Spectroscopy 3
3 UV/Vis Spectroscopy 11
4 ATR-FTIR and Raman Spectroscopy 13
5 ^{119}Sn Mössbauer Spectroscopy 14
6 Single Crystal X-Ray Diffraction....16
7 Quantum Chemical Investigations 28
8 References....49

1 Experimental Details

1.1 Synthesis

1.1.1 *$Sn(NEt_2)_2$*

In a Schlenk flask, 3.56 g anhydrous $SnCl_2$ (18.8 mmol, 1.0 eq) and 3.06 g Lithium diethylamide (97%, 37.5 mmol, 2.0 eq) were suspended in 100 mL Et_2O at -20°C. The brown suspension was stirred for 2 h at -20°C and was then allowed to slowly warm up to rt. The solvent was removed under reduced pressure, the remaining brownish grey solid was extracted with 100 mL *n*-pentane and filtered. The solvent was removed under reduced pressure to give yellow to light brown oil, which solidified upon cooling to -30°C.

Yield: 3.68 g (75%), light-brown oil (solidifies upon cooling to -30°C).

^{1}H NMR (300.22 MHz, C_6D_6) δ 3.52 (q, $^{3}J_{H,H}$ = 6.9 Hz, 8 H; 2xterm-N(C*H*$_2$CH$_3$)$_2$), 3.37 – 3.01 (m, 8 H; 2xμ_2-N(C*H*$_2$CH$_3$)$_2$), 1.20 (t, $^{3}J_{H,H}$ = 6.9 Hz, 12 H; 2xterm-N(CH$_2$C*H*$_3$)$_2$), 0.99 (m, 12 H; 24xμ_2-N(CH$_2$C*H*$_3$)$_2$) ppm. ^{13}C NMR (75.5 MHz, C_6D_6) δ 44.71 (*C*H$_2$CH$_3$), 42.84 (*C*H$_2$CH$_3$), 17.41 (CH$_2$*C*H$_3$), 11.92 (CH$_2$*C*H$_3$) ppm. ^{119}Sn NMR (111.92 MHz, C_6D_6) δ 103.82 ppm.

2 NMR Spectroscopy

2.1 NMR Spectra

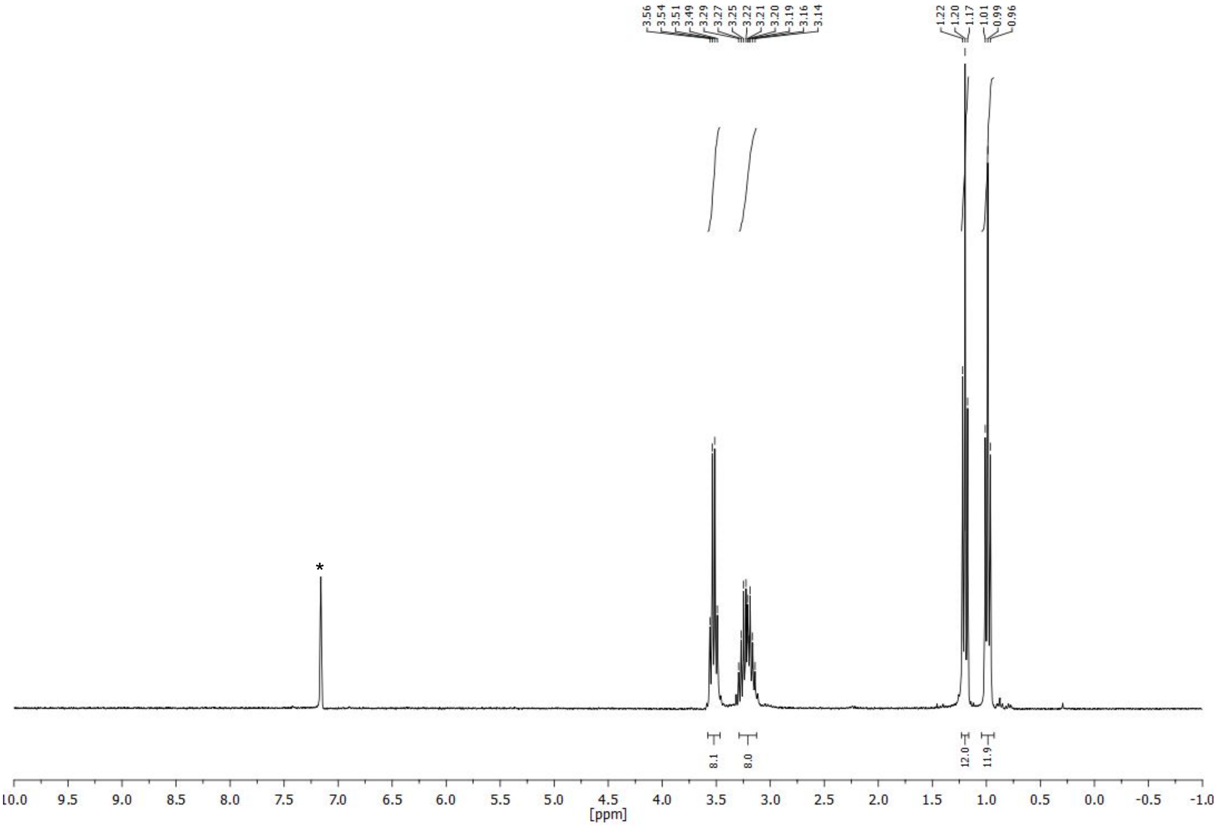

Figure S 1 ^{1}H NMR spectrum of $Sn(NEt_2)_2$ in C_6D_6 (* marks residual solvent peak). In solutions $Sn(NEt_2)_2$ exists in a dimeric structure of type $[(Et_2N)Sn(\mu\text{-}NEt_2)]_2$ leading to two inequivalent signals of NEt_2.

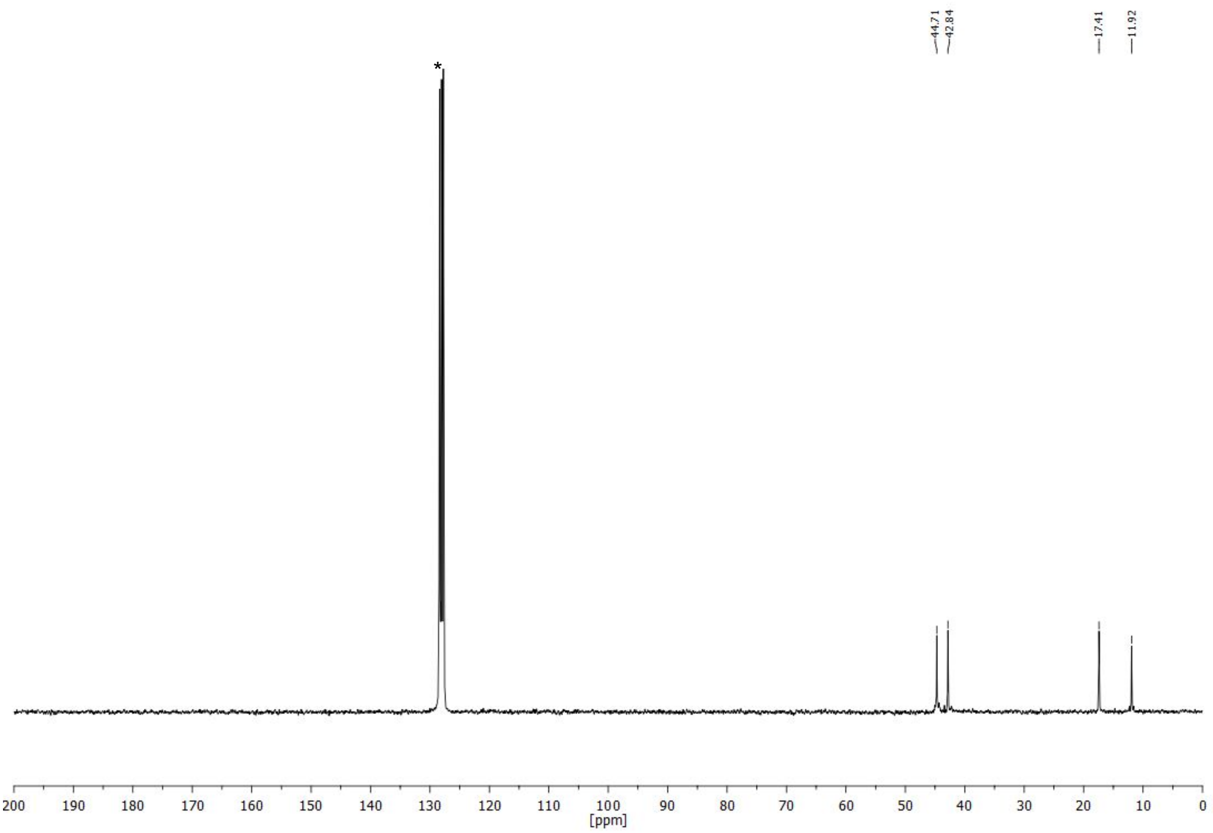

Figure S 2 ^{13}C NMR spectrum of $Sn(NEt_2)_2$ in C_6D_6 (* marks residual solvent peak). In solutions $Sn(NEt_2)_2$ exists in a dimeric structure of type $[(Et_2N)Sn(\mu\text{-}NEt_2)]_2$ leading to two inequivalent signals of NEt_2.

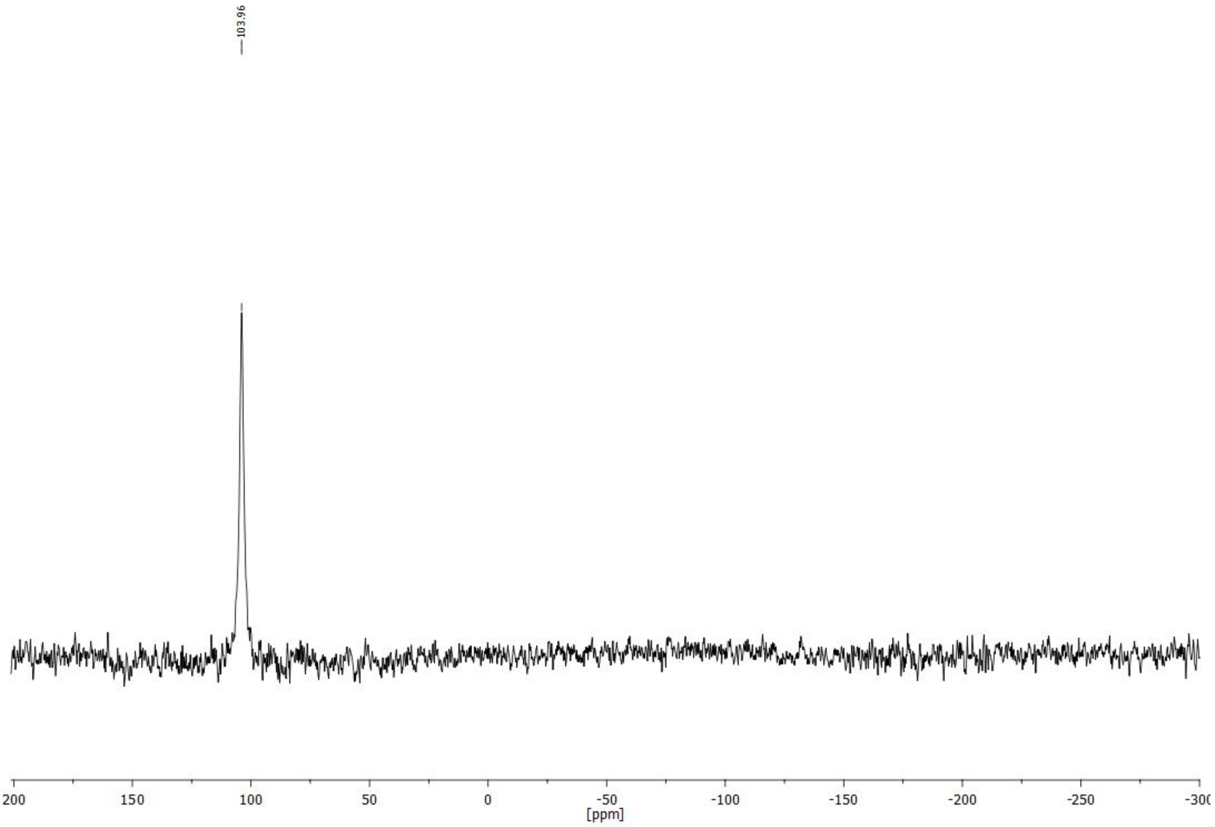

Figure S 3 ^{119}Sn NMR spectrum of $Sn(NEt_2)_2$ in C_6D_6.

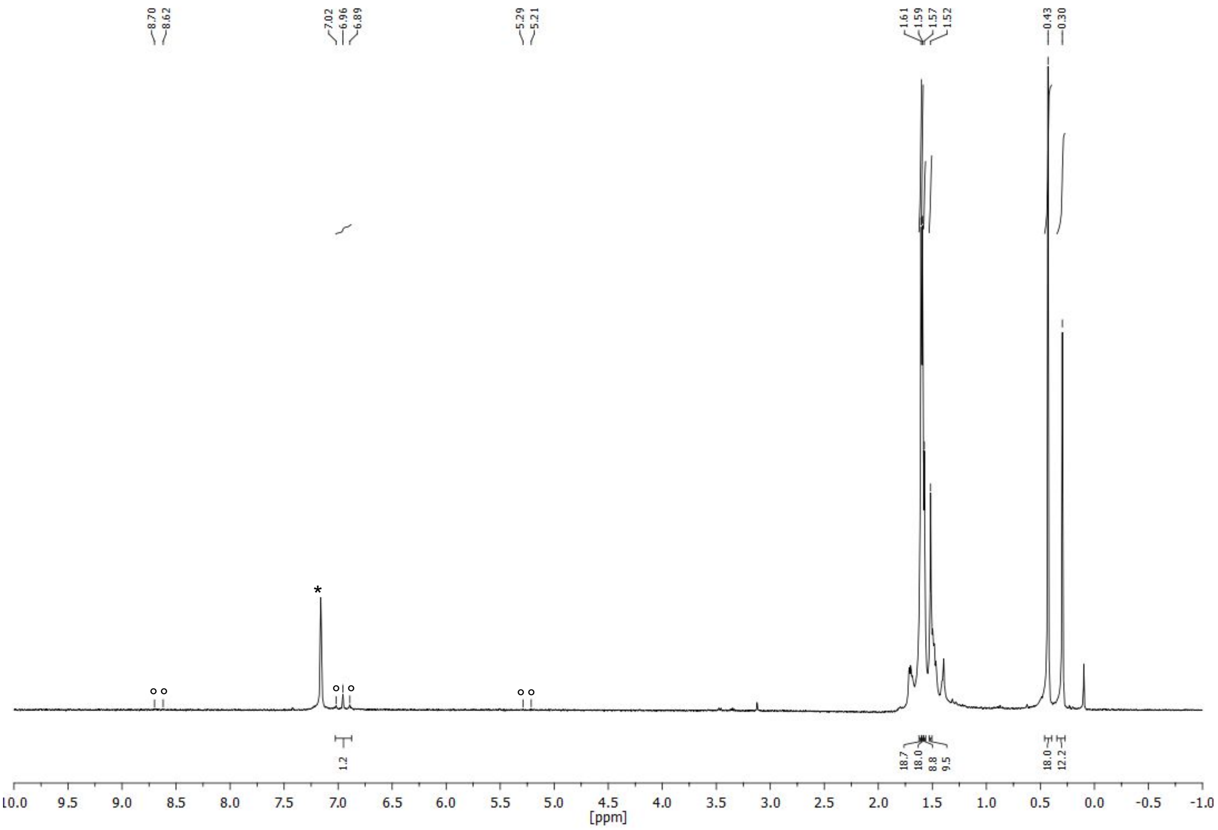

Figure S 4 ^{1}H NMR spectrum of $Sn_4tBu_6(H)[N(Si(CH_3)_3)_2]$ (**1**) in C_6D_6 (* marks residual solvent peak). The Sn*H* signal and coupling satellites are marked with °. ($^{1}J(^{1}H, ^{117/119}Sn)$ = 1001/1047 Hz, $^{2}J(^{1}H, Sn)$ = 38 Hz and $^{3}J(^{1}H, Sn)$ = 49 Hz).

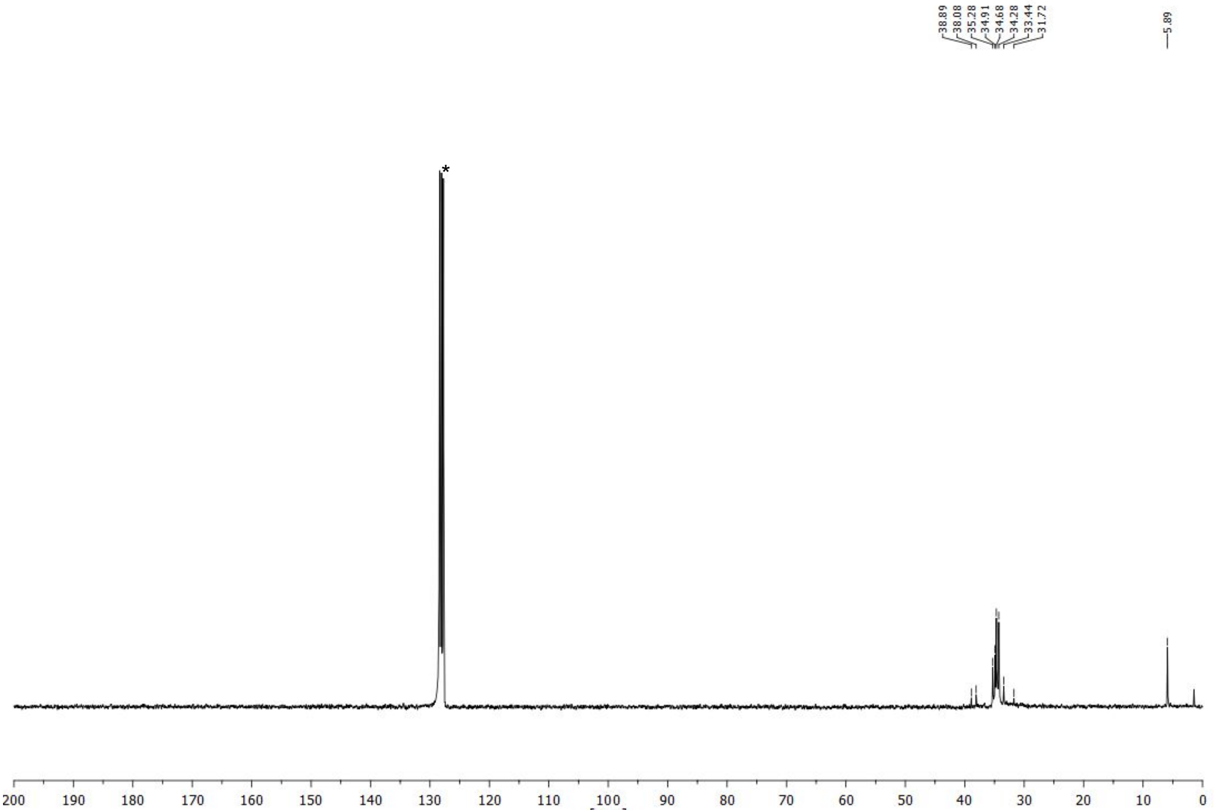

Figure S 5 ^{13}C NMR spectrum of $Sn_4tBu_6(H)[N(Si(CH_3)_3)_2]$ (**1**) in C_6D_6 (* marks residual solvent peak).

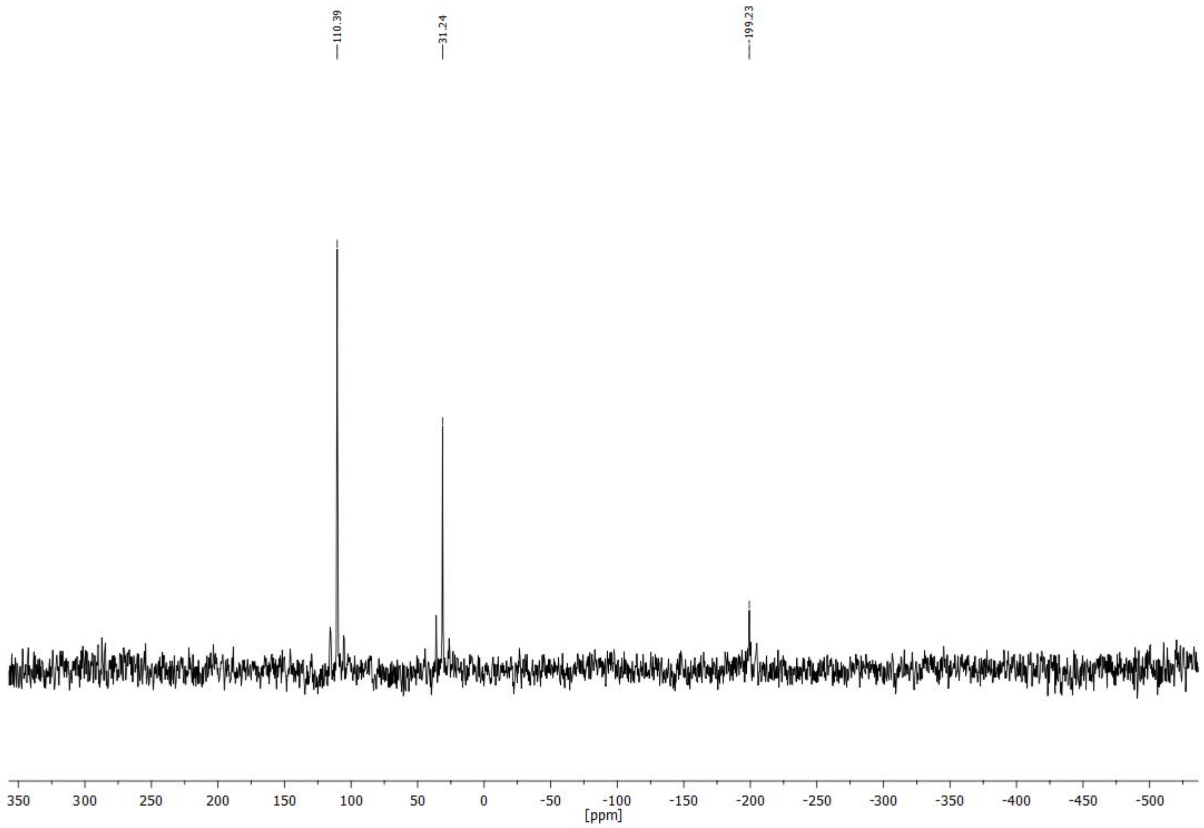

Figure S 6 $^{119}Sn\{^{1}H\}$ NMR spectrum of $Sn_4tBu_6(H)[N(Si(CH_3)_3)_2]$ (**1**) in C_6D_6.

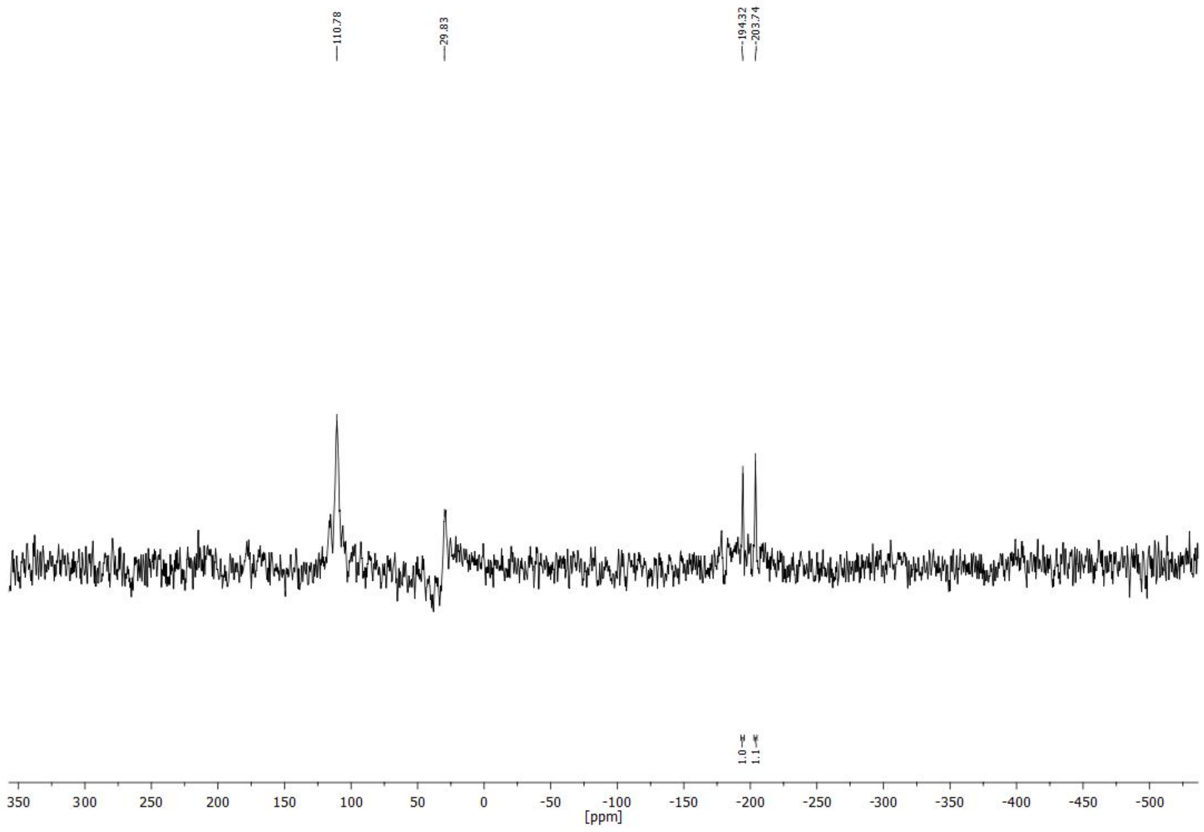

Figure S 7 ^{119}Sn NMR spectrum of $Sn_4tBu_6(H)[N(Si(CH_3)_3)_2]$ (**1**) in C_6D_6.

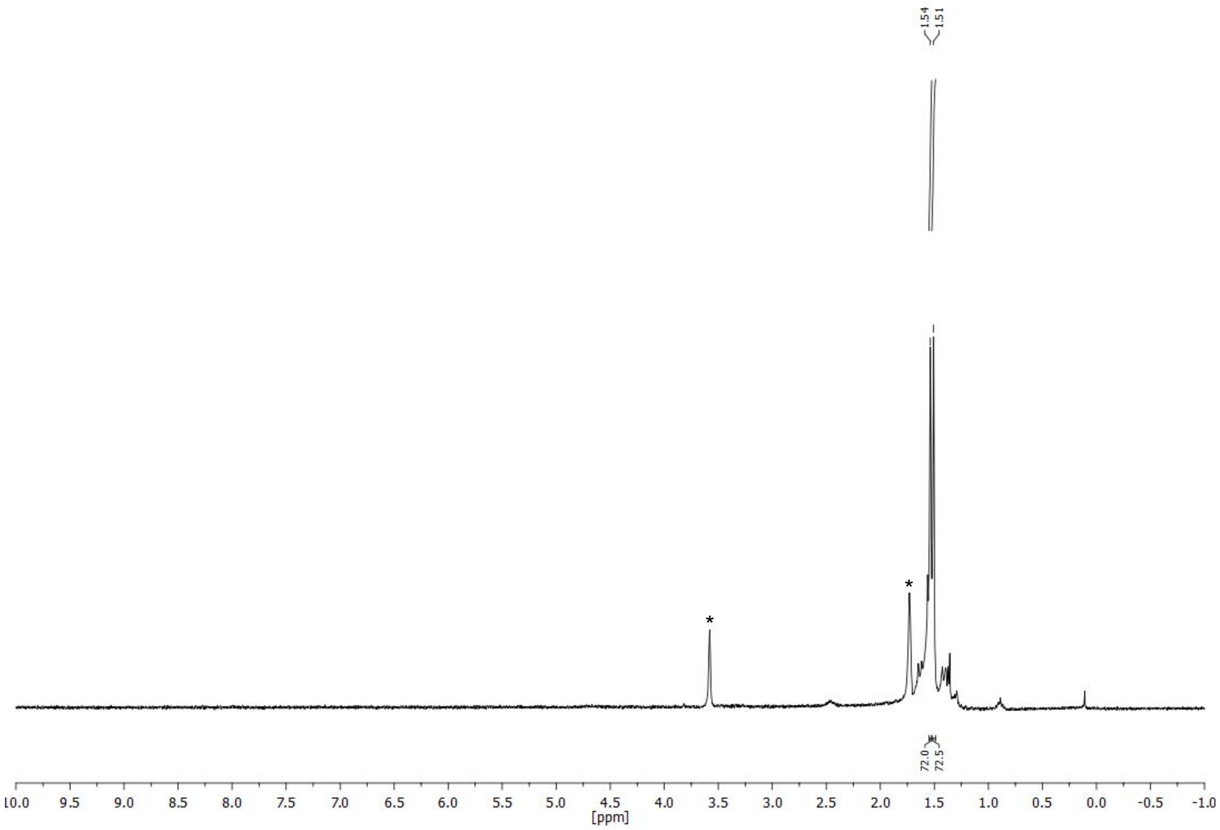

Figure S 8 ^{1}H NMR spectrum of $Sn_{16}tBu_{16}$ (**3**) in d_8-THF (* marks residual solvent peaks).

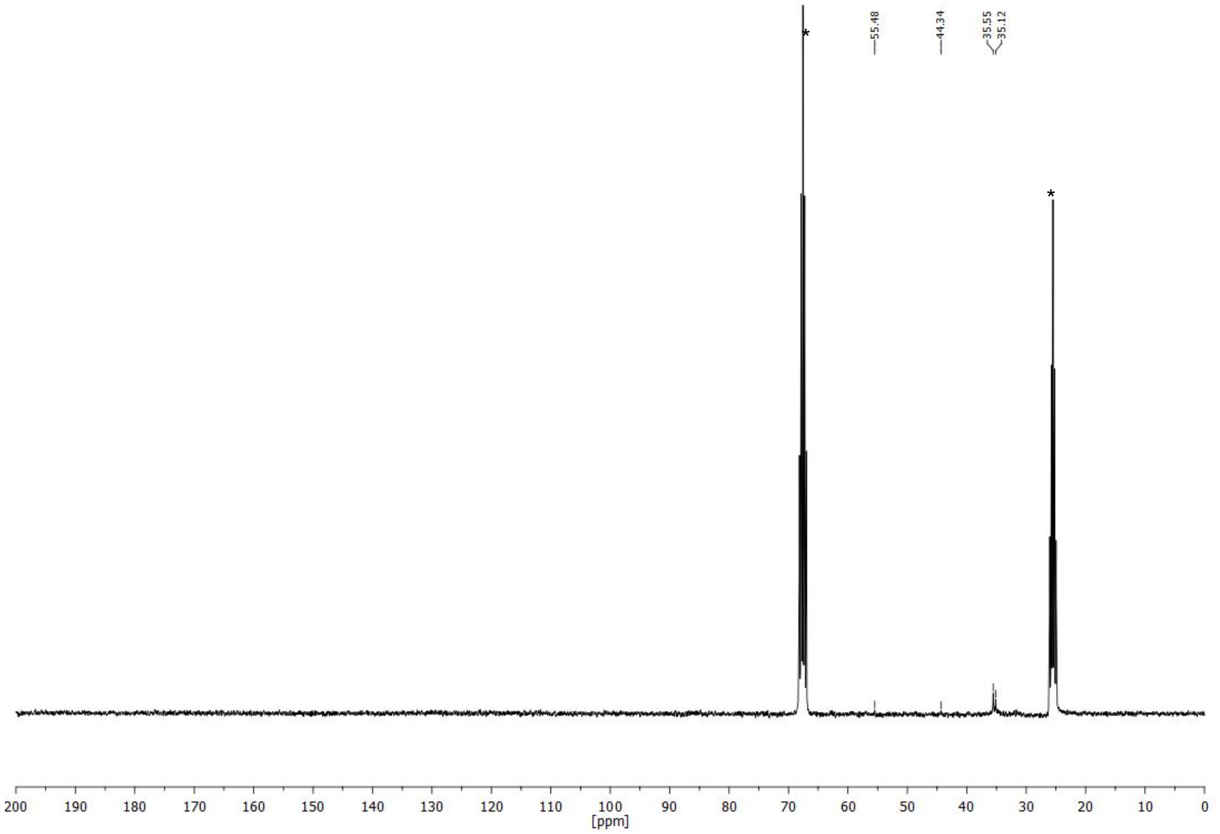

Figure S 9 ^{13}C NMR spectrum of $Sn_{16}tBu_{16}$ (**3**) in d_8-THF (* marks residual solvent peaks).

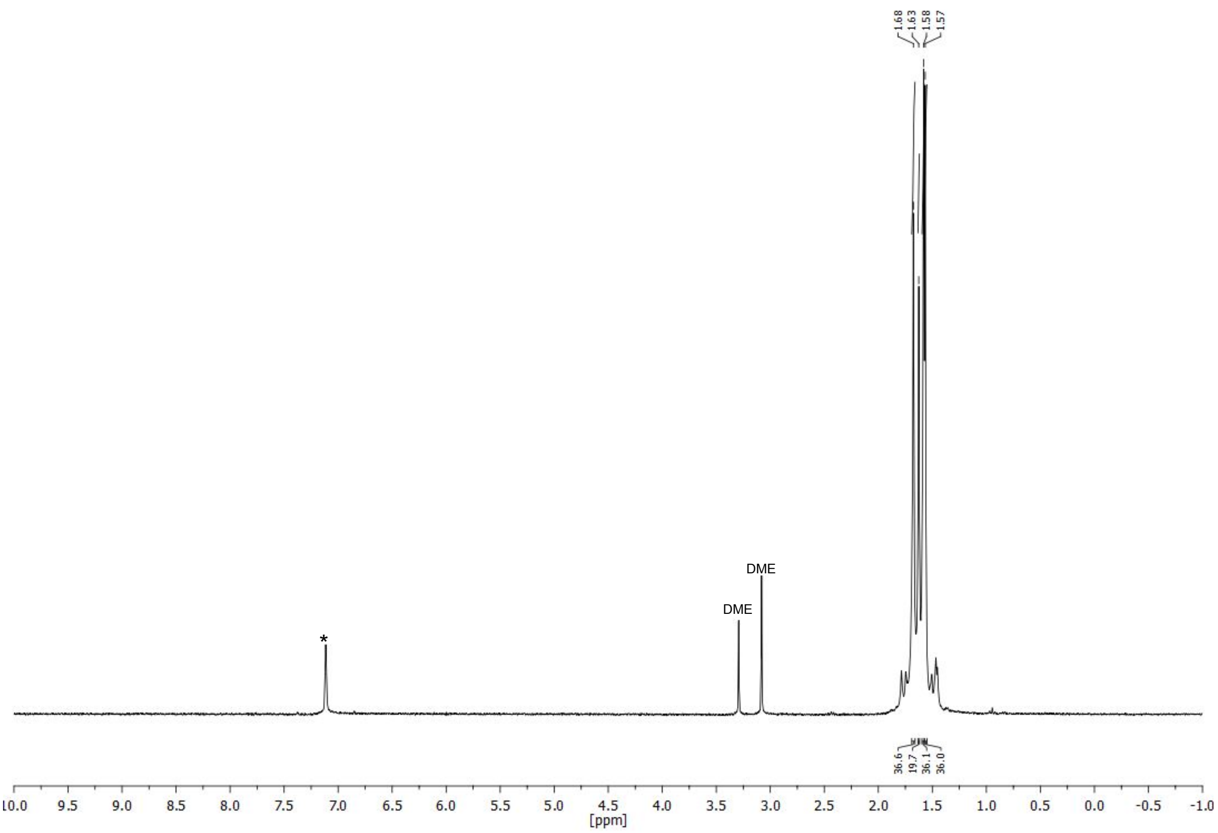

Figure S 10 ^{1}H NMR spectrum of $Sn_{15}tBu_{14}$ (**4**) in C_6D_6 (* marks residual solvent peak).

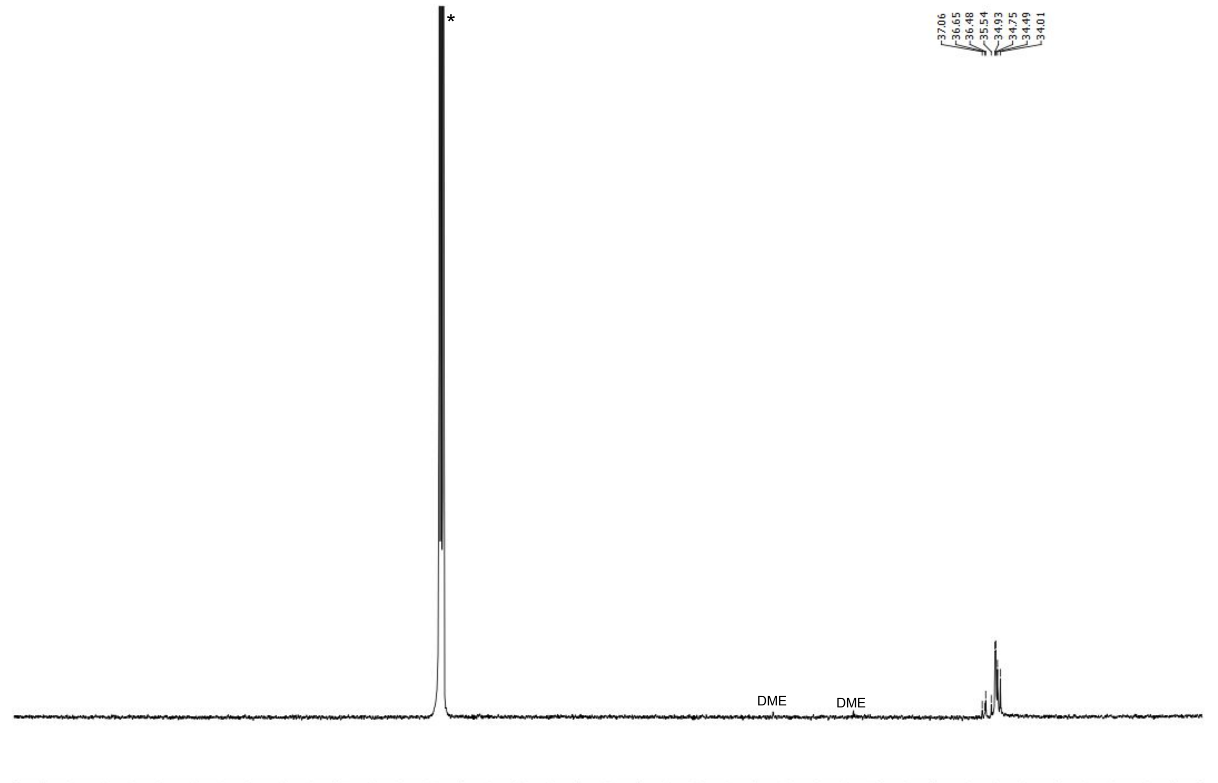

Figure S 11 ^{13}C NMR spectrum of $Sn_{15}tBu_{14}$ (**4**) in C_6D_6 (* marks residual solvent peak).

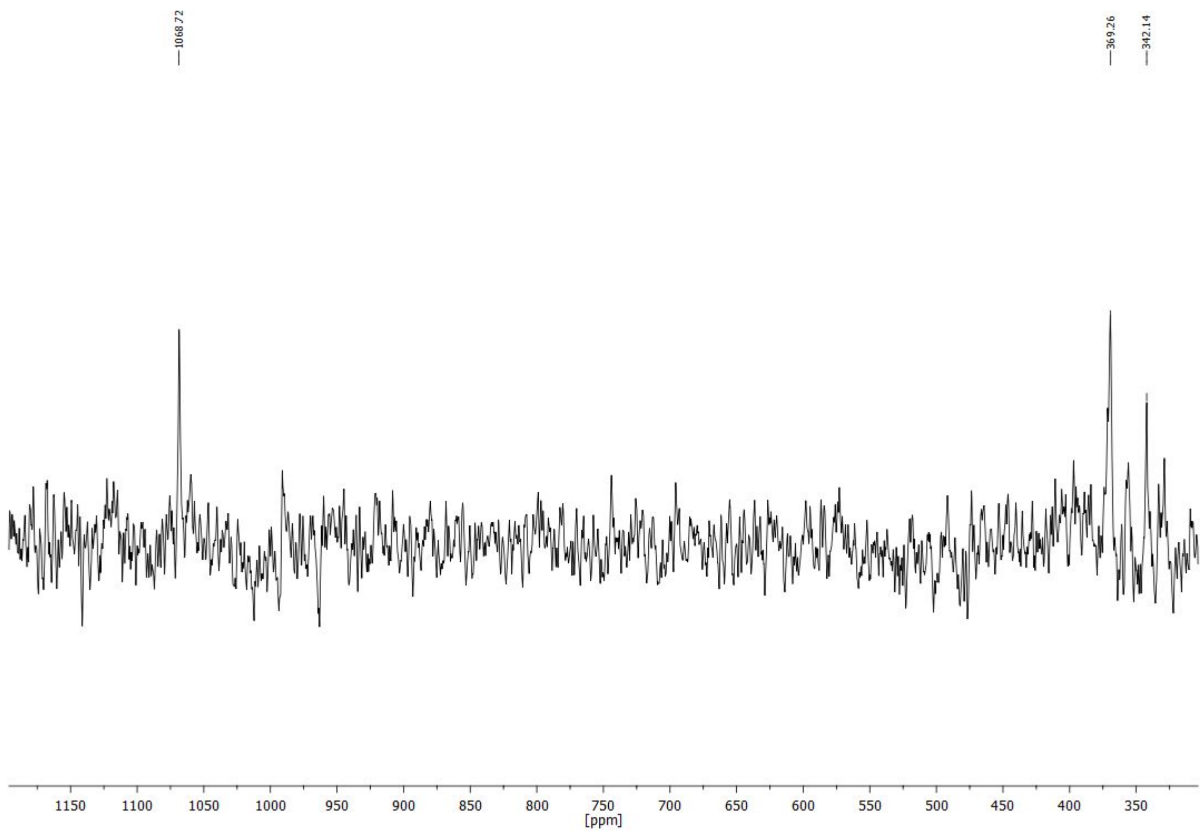

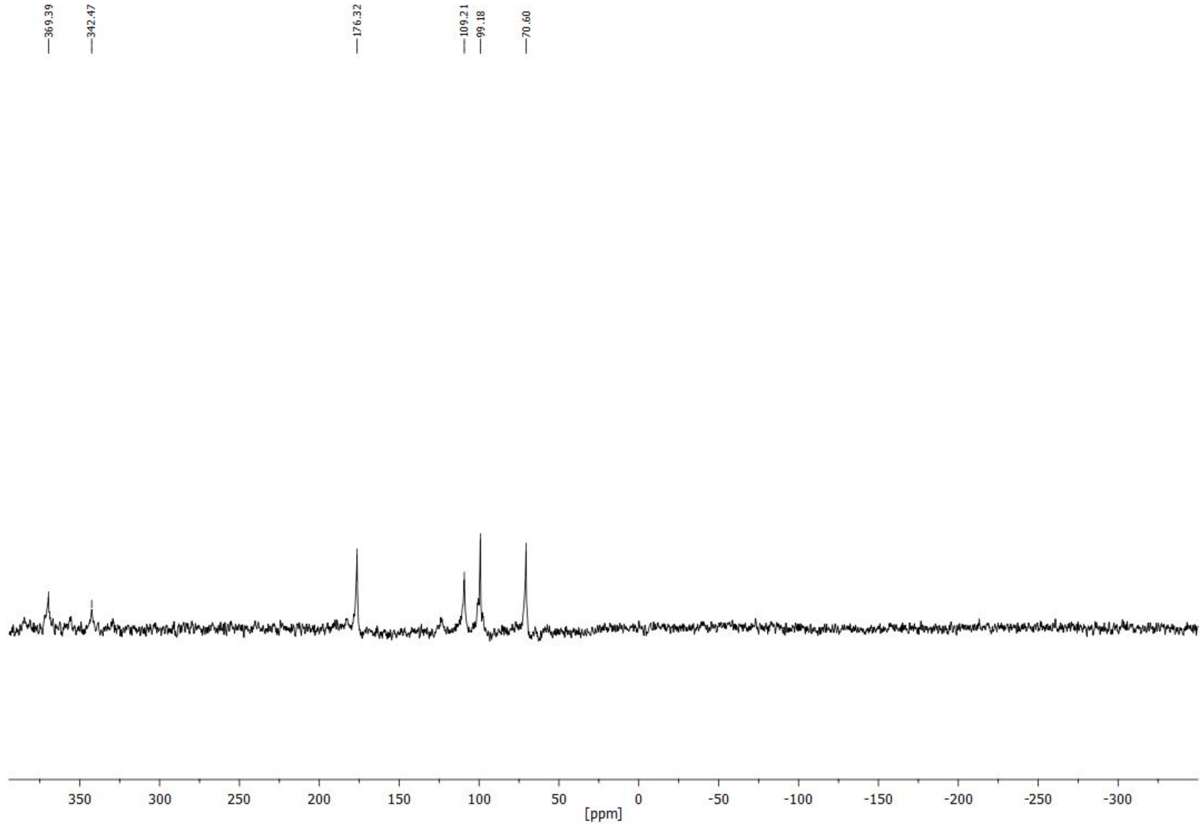

Figure S 12 ^{119}Sn NMR spectrum of Sn_{15}*t*Bu_{14} (**4**) in C_6D_6 (* marks residual solvent peak).

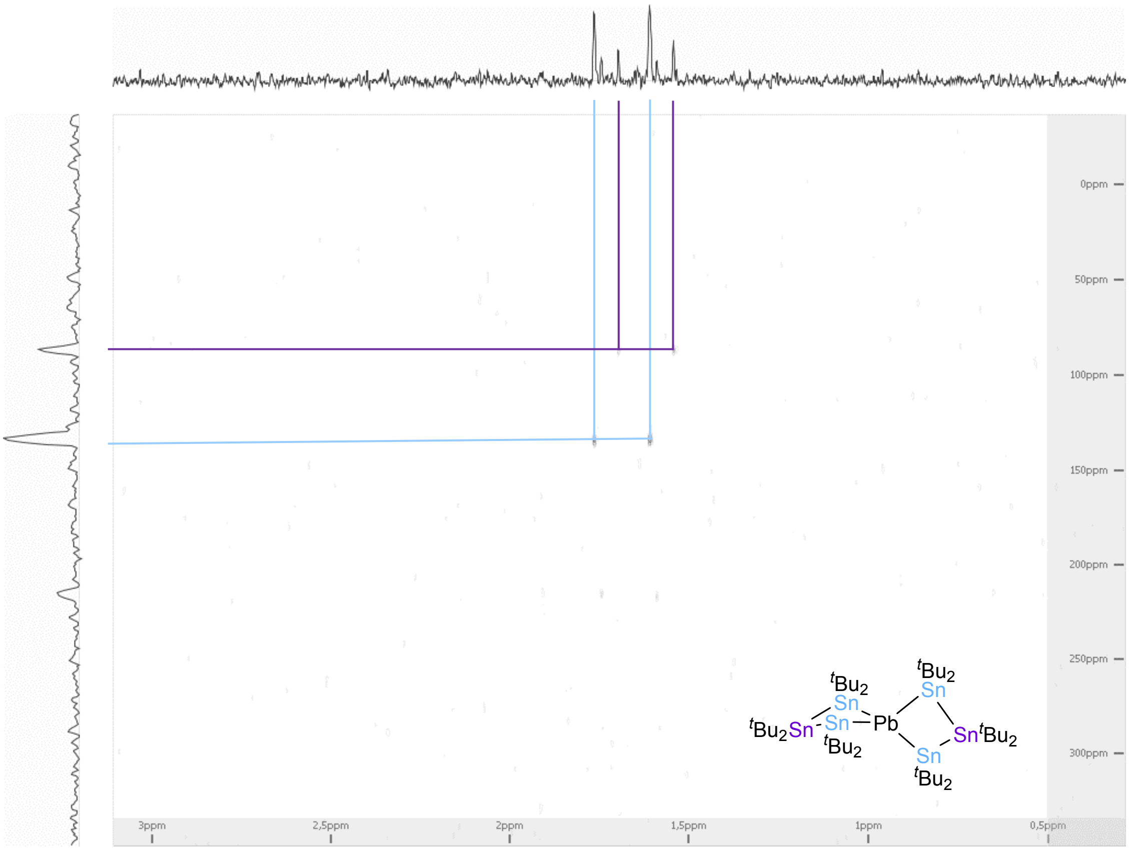

Figure S 13 2D-^{1}H,^{119}Sn-HMBC spectrum of **6** obtained from the reaction of $^{t}Bu_2HSnSnH^{t}Bu_2$ with $Pb[N(SiMe_3)_2]_2$. ^{119}Sn resonances at δ = 133.6 ppm and δ = 85.7 ppm.

3 UV/Vis Spectroscopy

3.1 UV-Vis Spectra

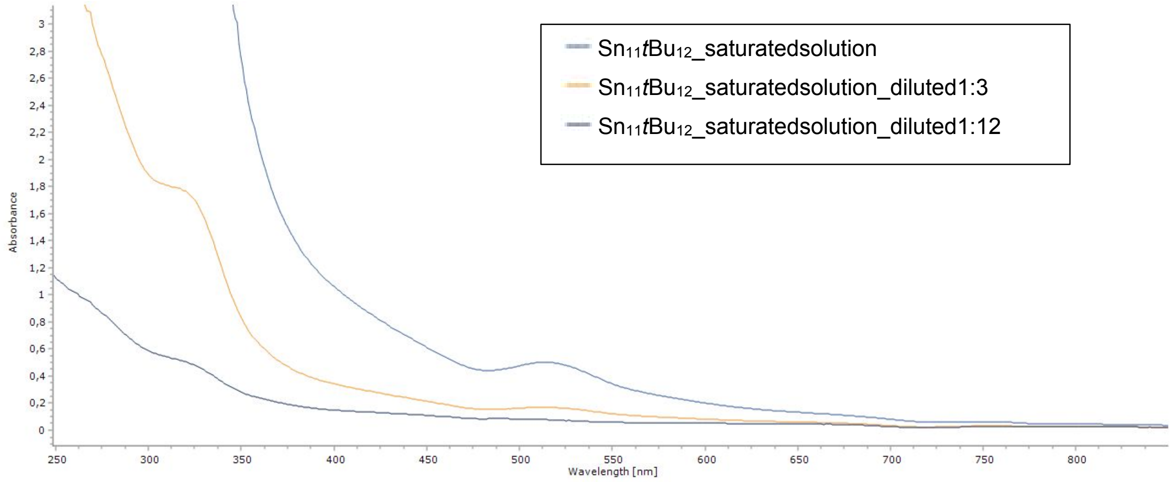

Figure S 14 UV-Vis spectra of $Sn_{11}tBu_{11}$ (**2**).

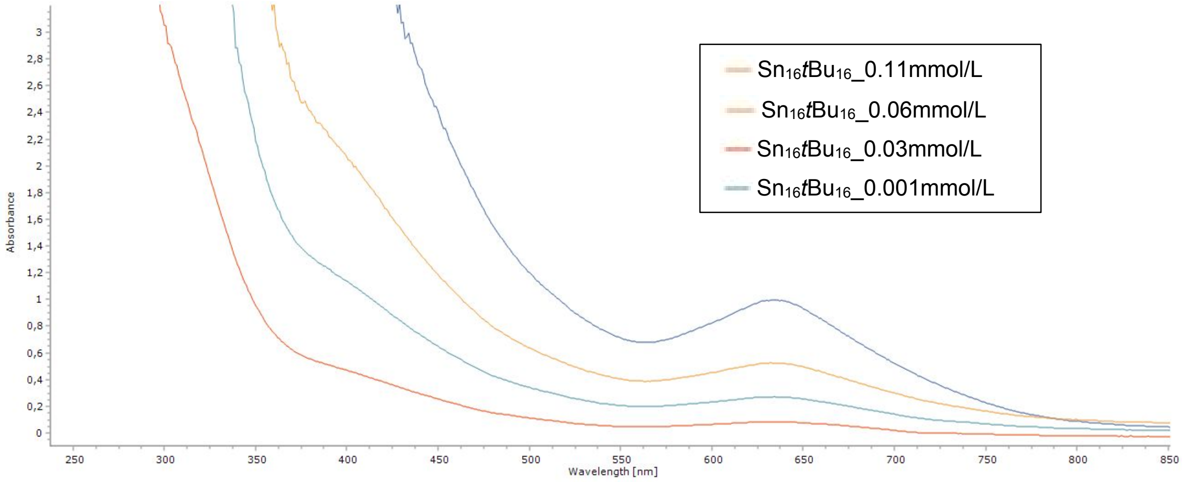

Figure S 15 UV-Vis spectra of $Sn_{16}tBu_{16}$ (**3**).

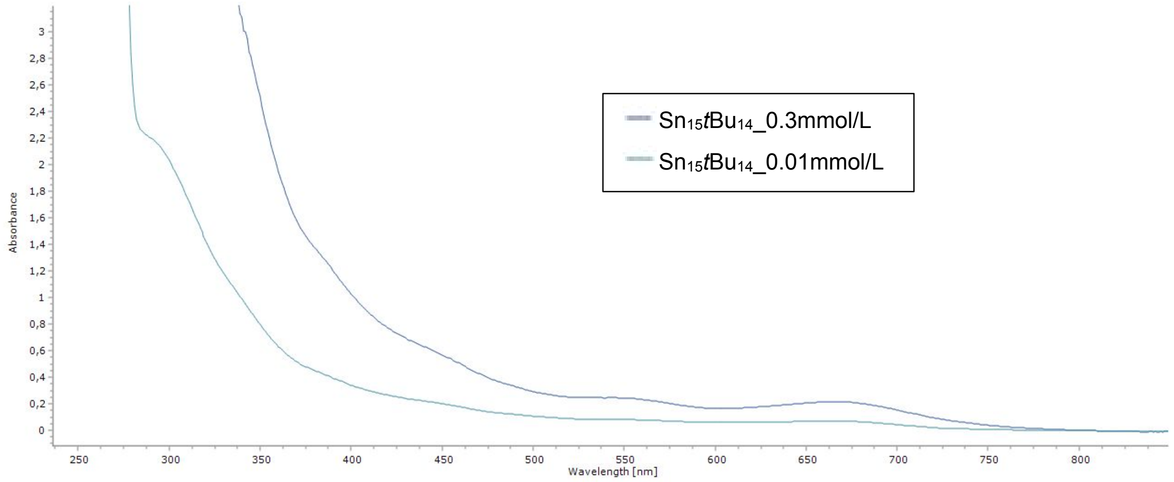

Figure S 16 UV-Vis spectra of $Sn_{15}tBu_{14}$ (**4**).

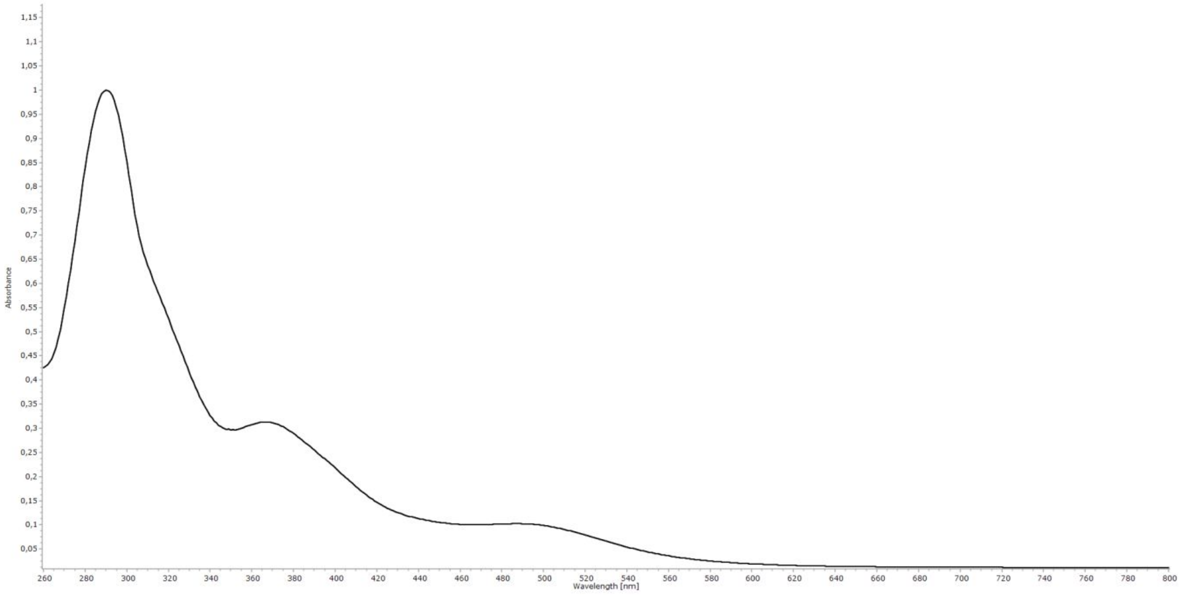

Figure S 16 UV-Vis spectrum of tBu$_6$Sn$_3$PbSn$_3$$tBu_6$ (**6**); absorption maximum at 286 nm originates from *cyclo*-Sn$_4$$tBu_8$, which is formed as second reaction product; λ_{max}(**6**) = 365 nm, 490 nm.

4 ATR-FTIR and Raman Spectroscopy

4.1 ATR-FTIR and Raman Spectra

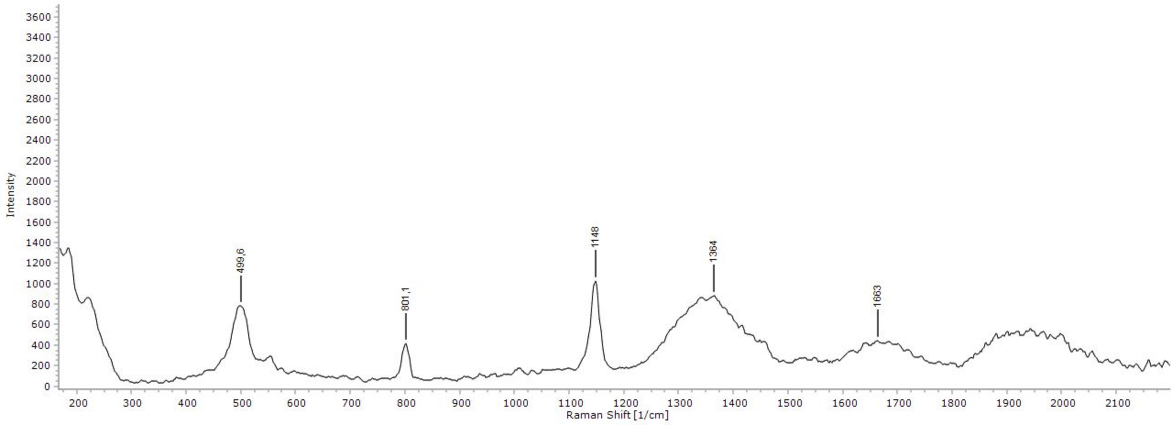

Figure S 17 Raman spectrum of $Sn_{11}tBu_{12}$ (**2**).

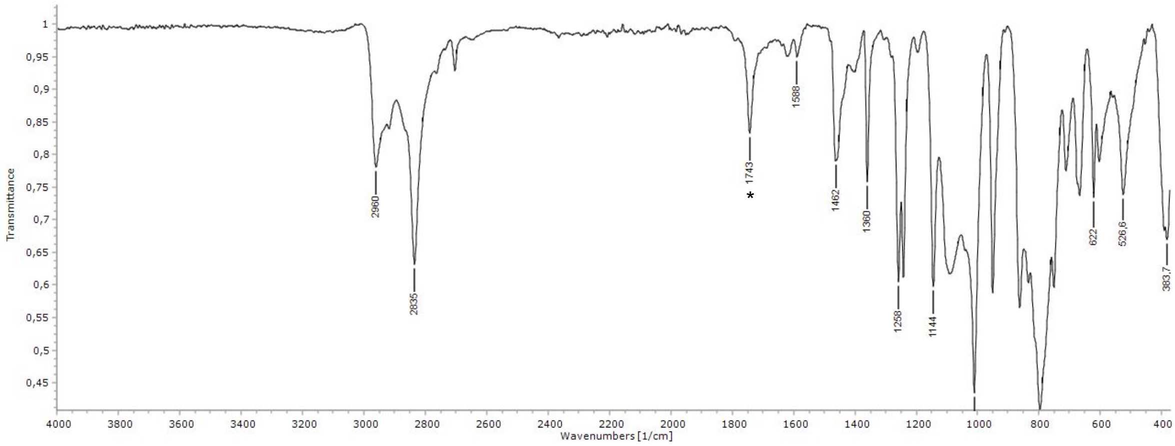

Figure S 18 ATR-FTIR spectrum of $Sn_4tBu_6(H)[N(Si(CH_3)_3)_2]$ (**1**). The * indicates the SnH vibration at 1743 cm^{-1}.

5 ^{119}Sn Mössbauer Spectroscopy

The ^{119}Sn Mössbauer spectrum of sample **2** (6 K data) is presented in Figure S 19 along with a transmission integral fit. The corresponding fitting parameters are summarized in Table S 1. The spectrum is well-resolved and could be fitted with a superposition of five sub-signals. The signals at 0.01(2) (purple line) and 3.756(8) (orange line) mm s^{-1} result from Sn^{IV} and Sn^{II} by-products, a consequence of the synthesis conditions and the high reactivity (hydrolyzability) of the sample. The remaining three sub-signals correspond to the three differently bonded tin sites in **2**. The course of the isomer shifts and the quadrupole splitting parameters are in line with the bonding pattern and the partials charge calculated theoretically. Consequently, the 4-bonded tin atoms (approximately tetrahedral geometry) have the smallest quadrupole splitting parameter. On the other hand, the largest quadrupole splitting parameter was detected for the 2-bonded tin atoms. The ratio of these three sub-signals (4b:3b:2b = 3:2:6) was kept fixed during the fitting procedure.

5.1 ^{119}Sn Mössbauer Spectrum of 2

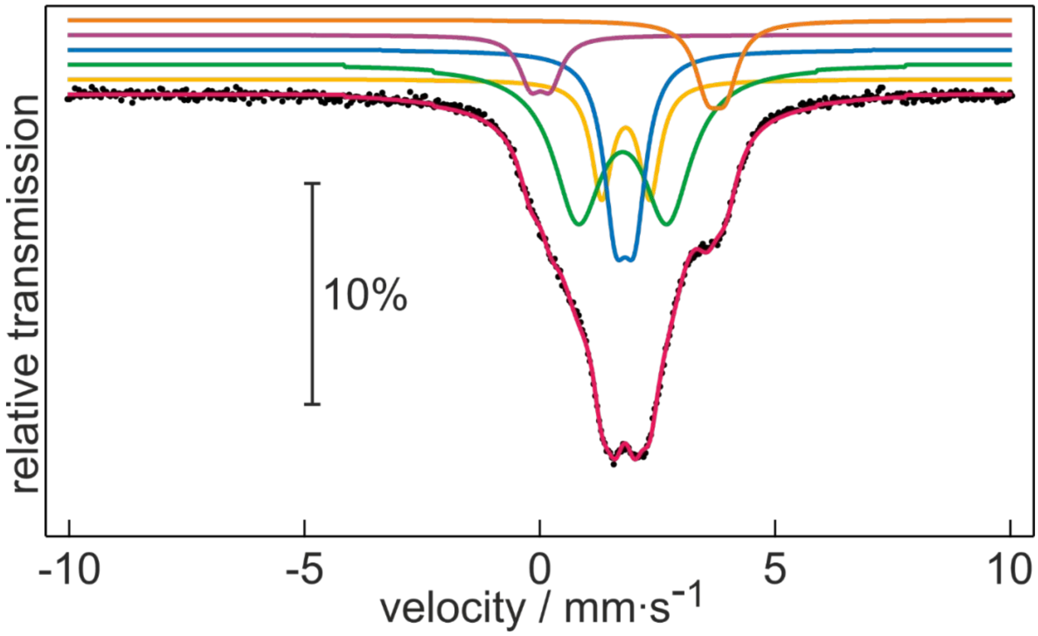

Figure S 19 Experimental (dots) and simulated (colored lines) ^{119}Sn Mössbauer spectrum of $Sn_{11}\textit{t}Bu_{12}$ (**2**) at 6 K.

Table S 1 Fitting parameters of a ^{119}Sn Mössbauer spectroscopic measurements for compound **2** at 6 K. δ = isomer shift, ΔE_Q = quadrupole splitting parameter, Γ = experimental line width. The signal contributions (tin numbers according to Figure S 21; kept constrained during the fitting procedure) and the area percentages are also listed.

Signal	Contribution	δ [mm*s^{-1}]	ΔE_Q [mm*s^{-1}]	Γ [mm*s^{-1}]	Signal [%]
Yellow	Sn1/Sn2	1.827(4)	1.02(1)	0.53(2)	15(1)
Green	Sn4/Sn5/Sn7/Sn8/Sn10/Sn11	1.76(1)	1.90(2)	1.24(3)	46
Blue	Sn3/Sn6/Sn9	1.810(5)	0.404(6)	0.60(2)	23
Purple	Sn^{IV} byproduct	0.01(2)	0.43(2)	0.58(4)	6(1)
Orange	Sn^{II} byproduct	3.756(8)	0.38(2)	0.64(3)	9(1)

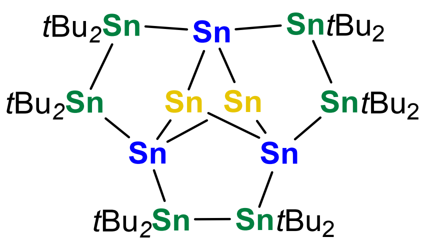

6 Single Crystal X-Ray Diffraction

6.1 Crystal Data and Structure Refinement

Table S 2 Crystal data and structure refinement of compounds **1** and **2**.

Compound	$Sn_4tBu_6N(SiMe_3)_2H$ (**1**)	$Sn_{11}\mathit{t}Bu_{12}$ (**2**)
CCDC deposition number	2533596	2533597
Empirical formula	$C_{30}H_{73}NSi_2Sn_4$	$C_{48}H_{108}Sn_{11}$
Formula weight	978.83	1990.93
Temperature/K	99.97	100
Crystal system	monoclinic	triclinic
Space group	$P2_1/c$	$P1$
a/Å	13.9316(15)	9.3514(4)
b/Å	9.1971(9)	14.4819(6)
c/Å	33.420(4)	15.2010(6)
α/°	90	109.809(2)
β/°	94.245(6)	106.775(2)
γ/°	90	103.965(2)
Volume/Å^3	4270.3(8)	1717.93(13)
Z	4	1
ρ_{calc}g/cm^3	1.52	1.92
μ/mm^{-1}	2.4	3.9
F(000)	1952	946
Crystal size/mm^3	0.29 × 0.09 × 0.07	0.18 × 0.09 × 0.07
Radiation	MoKα (λ = 0.71073)	MoKα (λ = 0.71073)
2θ range for data collection/°	2.444 to 53.998	3.226 to 51.996
Index ranges	$-17 \le h \le 17$, $-11 \le k \le 11$, $-42 \le l \le 42$	$-11 \le h \le 11$, $-17 \le k \le 17$, $-18 \le l \le 18$
Reflections collected	66849	93854
Independent reflections	9298 [R_{int} = 0.0817, R_{sigma} = 0.0592]	13163 [R_{int} = 0.0806, R_{sigma} = 0.0547]
Data/restraints/parameters	9298/175/362	13163/657/900
Goodness-of-fit on F^2	1.070	1.058
Final R indexes [I>=2σ (I)]	R_1 = 0.0826, wR_2 = 0.2385	R_1 = 0.0442, wR_2 = 0.1035
Final R indexes [all data]	R_1 = 0.0849, wR_2 = 0.2402	R_1 = 0.0501, wR_2 = 0.1065
Largest diff. peak/hole / e Å^{-3}	2.43/-2.52	4.34/-1.41
Flack parameter		0.14(3)

Table S 3 Crystal data and structure refinement of compounds **3** and **4**.

Compound	Sn_{15}*t*Bu_{14} (**4**)	Sn_{16}*t*Bu_{16} (**3**)
CCDC deposition number	2533599	2533598
Empirical formula	$C_{56}H_{124}Sn_{15}$ + solvent (C_7H_8)	$C_{72}H_{164}O_4Sn_{16}$
Formula weight	2670.03	2993.06
Temperature/K	100.02	100
Crystal system	monoclinic	monoclinic
Space group	*C*2/*c*	$P2_1$
a/Å	18.3916(11)	17.9297(5)
b/Å	24.6008(11)	29.8905(9)
c/Å	20.4455(10)	20.9772(6)
β/°	107.949(4)	112.8530(10)
Volume/Å^3	8800.3(8)	10359.8(5)
Z	4	4
ρ_{calc}g/cm^3	2.02	1.92
μ/mm^{-1}	4.2	3.8
F(000)	5040	5712
Crystal size/mm^3	0.14 × 0.11 × 0.07	0.2 × 0.13 × 0.09
Radiation	MoKα (λ = 0.71073)	MoKα (λ = 0.71073)
2θ range for data collection/°	3.088 to 53.996	2.106 to 59.216
Index ranges	$-23 \le h \le 23$, $-31 \le k \le 31$, $-26 \le l \le 26$	$-24 \le h \le 24$, $-41 \le k \le 41$, $-29 \le l \le 29$
Reflections collected	212467	364583
Independent reflections	9617 [R_{int} = 0.0484, R_{sigma} = 0.0179]	57999 [R_{int} = 0.0537, R_{sigma} = 0.0384]
Data/restraints/parameters	9617/138/429	57999/282/1857
Goodness-of-fit on F^2	1.157	1.072
Final R indexes [I>=2σ (I)]	R_1 = 0.0580, wR_2 = 0.1113	R_1 = 0.0256, wR_2 = 0.0562
Final R indexes [all data]	R_1 = 0.0782, wR_2 = 0.1331	R_1 = 0.0286, wR_2 = 0.0575
Largest diff. peak/hole / e Å^{-3}	2.09/-1.60	1.97/-1.10
Flack parameter		-0.014(8)

Table S 4 Crystal data and structure refinement of compounds **5** and **6**.

Compound	*t*$Bu_6Sn_3PbSn_3$*t*Bu_6 (**6**)	*cyclo*-Sn_4*t*Bu_8 (**5**)
CCDC deposition number	2533601	2533600
Empirical formula	$C_{48}H_{108}PbSn_6$	$C_{32}H_{72}Sn_4$
Formula weight	1604.67	931.65
Temperature/K	100.01	99.98(18)
Crystal system	monoclinic	monoclinic
Space group	$P2_1/n$	$C2/m$
a/Å	20.5850(16)	18.2871(3)
b/Å	23.2338(19)	12.5320(2)
c/Å	26.8347(18)	9.3565(2)
β/°	104.905(2)	114.833(2)
Volume/Å^3	12402.3(16)	1946.00(7)
Z	8	2
ρ_{calc}g/cm^3	1.72	1.59
μ/mm^{-1}	5.1	20.3
F(000)	6224	928
Crystal size/mm^3	0.09 × 0.08 × 0.02	0.07 × 0.06 × 0.02
Radiation	MoKα (λ = 0.71073)	Cu Kα (λ = 1.54184)
2θ range for data collection/°	2.236 to 54	8.842 to 157.28
Index ranges	$-26 \le h \le 26$, $-29 \le k \le 29$, $-34 \le l \le 30$	$-22 \le h \le 23$, $-15 \le k \le 15$, $-11 \le l \le 9$
Reflections collected	236216	17344
Independent reflections	27015 [R_{int} = 0.1195, R_{sigma} = 0.0814]	2176 [R_{int} = 0.0699, R_{sigma} = 0.0344]
Data/restraints/parameters	27015/48/1084	2176/81/154
Goodness-of-fit on F^2	1.065	1.084
Final R indexes [I>=2σ (I)]	R_1 = 0.0406, wR_2 = 0.0716	R_1 = 0.0338, wR_2 = 0.0847
Final R indexes [all data]	R_1 = 0.1034, wR_2 = 0.0944	R_1 = 0.0359, wR_2 = 0.0862
Largest diff. peak/hole / e Å^{-3}	2.02/-2.07	1.87/-1.08

6.2 Solid-State Structures

6.2.1 *$Sn_4tBu_6[N(SiMe_3)_2](H)$ (1)*

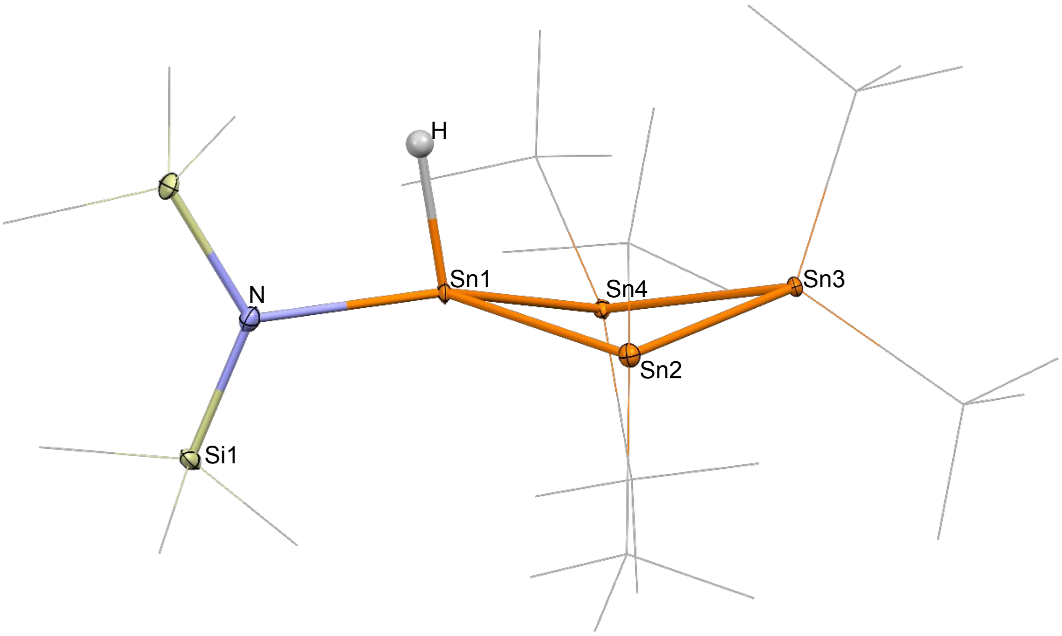

Figure S 20 Molecular structure of Sn_4*t*$Bu_6[N(SiMe_3)_2](H)$ (**1**). All non-carbons shown as 30% shaded ellipsoids. Hydrogen atoms except Sn-H are omitted for clarity. Selected bond lengths [Å] and angles [°] for **1**: Sn1-Sn2 2.831(1), Sn1-Sn4 2.818(1), Sn2-Sn3 2.880(1), Sn3-Sn4 2.886(1), Sn1-N 2.12(1), Sn1-H 1.68(4), Sn1-Sn2-Sn3 83.43(4), Sn1-Sn4-Sn3 83.56(4), Sn2-Sn3-Sn4 92.21(4), Sn2-Sn1-Sn4 94.70(4), Sn2-Sn1-N1 130.4(4), Sn4-Sn1-N1 127.9(4), N1-Sn1-H 88(4).

$Sn_4tBu_6(N(SiMe_3)_2)(H)$ (**1**) crystallizes in the monoclinic space group $P2_1/c$ as a slightly folded (152.52°) four membered ring (Figure S 20). Also other unsymmetrically substituted *cyclo*-Sn_4 compounds exhibit similar folding angles (Sn_4tBu_7Me: 157.09 to 157.71°; Sn_4tBu_7Bu: 156.64°), while parent octa-*t*Bu-tetrastannacyclobutane Sn_4tBu_8 is planar.[1,2] The Sn-Sn bond lengths range from 2.818(1) to 2.886(1) Å agreeing with values typically found for Sn-Sn single bonds. Yet, Sn-Sn bonds of still functional Sn1 are slightly shorter (Sn1-Sn2 2.831(1) Å, Sn1-Sn4 2.818(1) Å) than distances of *t*Bu substituted Sn3 (Sn2-Sn3 2.880(1) Å, Sn3-Sn4 2.886(1) Å). While the experimentally determined Sn-H bond distance (1.68(4)°) falls within the range of typical Sn-H bond distances (it should be noted that Sn-H bond length is systematically underestimated by crystallographic refinement), the observed Sn-N bond (2.12(2) Å) is extremely elongated comparing to Sn-N bonds of other tetracoordinate tin compounds (e.g. $MeSn[N(SiMe_3)_2]_3$[3] 2.067 Å, (η_2-$C_{12}H_{14}$)$Sn[N(SiMe_3)_2]_2$[4] 2.068(2) Å), but similarly long as in $[(C_4H_4N)(Me_3Si)_2C]Sn[N(SiMe_3)_2]$ (2.144(4) Å),[5] which exhibits an oxidation state of +II. The C-Sn-C angles of Sn2, Sn3 and Sn4 match the values expected for tetrahedral arrangement (107.4(6) to 109.0(5)°), but Sn-Sn-Sn angles within the 4-membered ring are strongly acute (83.43(4) to 92.21(4)°) owing the distorted rectangular arrangement of the Sn4 ring. Angles around Sn1 are extremely distorted from the expected tetragonal arrangement (Sn2-Sn1-Sn4 94.70(4), Sn2-Sn1-N 130.4(4)°, N-Sn1-H 88(4)°) nearly reaching a pyramidal arrangement. The nitrogen atom attached to Sn1 exhibits a planar coordination environment.

6.2.2 *Sn₁₁tBu₁₂ (2)*

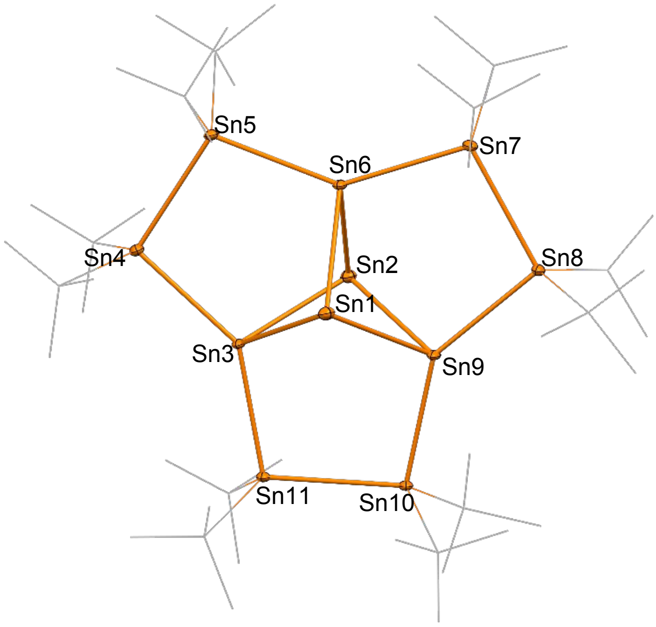

Figure S 21 Molecular structure of Sn_{11}*t*Bu_{12} (**2**). All non-carbons shown as 30% shaded ellipsoids. Hydrogen atoms are omitted for clarity. Selected bond lengths [Å] and angles [°] for **2**: Sn1···Sn2 3.260(2), Sn1-Sn3 2.816(1), Sn1-Sn6 2.821(2), Sn1-Sn9 2.811(2), Sn3-Sn4 2.804(2), Sn3-Sn11 2.807(2), Sn4-Sn5 2.911(1), Sn5-Sn6 2.802(2), Sn6-Sn7 2.797(1), Sn7-Sn8 2.912(2), Sn8-Sn9 2.805(1), Sn9-Sn10 2.800(1), Sn10-Sn11 2.908(1), Sn1-Sn3-Sn2 70.66(4), Sn3-Sn1-Sn6 89.76(4), , Sn4-Sn3-Sn11 142.45(5), Sn3-Sn4-Sn5 102.20(4), C1-Sn4-C5 109.7(7).

Despite its highly symmetrical structure, $Sn_{11}tBu_{12}$ (**2**) crystallizes in the low symmetry space group *P*1 owed to the slightly distorted triangular molecular shape. Yet, not considering organic substituents **2** exhibits nearly an ideal D_{3h} symmetry. The Sn_{11} backbone can be described as six edge-sharing five membered rings and its core structure (Sn1, Sn3, Sn2, Sn6 and Sn9) resembles the tin backbone of pentastanna[1.1.1]propellanes Sn_5R_6. In theory, **2** matches a propellane-type Sn_5 backbone with R_2SnSnR_2 units added to each butterfly-type four membered ring. Interestingly, SITA and coworkers have isolated a compound Sn_7Dep_8 matching a propellane structure with only one R_2SnSnR_2 unit added.[6] (Figure S 21) Comparing **2** to these structures, its $Sn^b\cdots Sn^b$ distance (3.260(2) Å) follows the trend observed in Sn_5R_6 (R = Dep; 3.367(3) Å) to Sn_7Dep_8 (R = Dep; 3.348(5) Å) as addition of a R_2SnSnR_2 unit seems to lead to a decrease in the distance between bridgehead atoms and therefore making interaction between these atoms even more likely. The shortened distance is accompanied by an expected decrease of Sn^b-Sn-Sn^b angles (**2**: 70.66(4) to 70.73(4)°; Sn_5Dep_6: 72.24(6) to 72.09(6)°; Sn_7Dep_8: 71.6(1) to 72.4(1)°). The Sn-Sn bond lengths between formally Sn^0 to Sn^0 as well as bond distances between R_2Sn-Sn in **2** are overall shortened (2.797(1) to 2.823(2) Å) compared to respective bond distances in Sn_5Dep_6 (2.87(3) to 2.848(2) Å) and Sn_7Dep_8 (2.818(3) to 2.862(5) Å), corresponding well to bond lengths found in α-tin (2.80 Å).[7] In contrast, tBu_2Sn-SntBu_2 bond distances are elongated by somewhat 0.1 Å (2.912(2) to 2.908(1) Å), agreeing with a Dep_2Sn-SnDep_2 bond distance of 2.901(4) Å in Sn_7Dep_8. While already discussed Sn^b-Sn-Sn^b angles of Sn3, Sn6 and Sn9 in **2** are strongly acute, tBu_2Sn-Sn-SntBu_2 angles of these tin atoms deviate extremely from the ideal tetrahedral arrangement (109.5°) with values between 141.61(5) and 142.45(5)°. Interestingly, Sn3, Sn6 and Sn9 and the tin atoms in tBu_2SnSntBu_2 bridges are almost perfectly coplanar, with torsion angles ranging from 0.75(5) to 1.71(5)°, while in Sn_7Dep_8 a dihedral angle of 13.9(1)° is observed. The brownish orange needles of **2** exhibit extremely poor solubility in common organic solvents, which will be discussed later in this section.

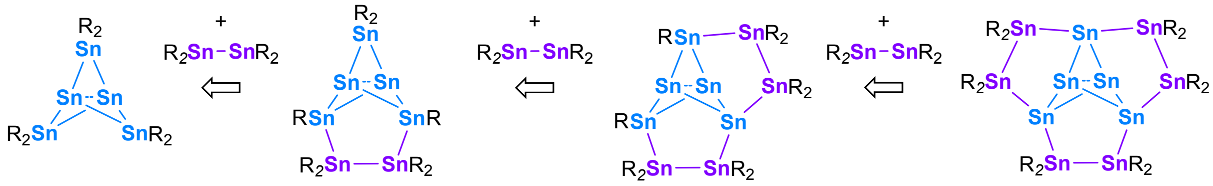

Sn_5R_6 — known for various R, e.g. R = Dep

$Sn_5Sn_2R_8 = Sn_7R_8$ — known for R = Dep

$Sn_5Sn_2R_4Sn_4R_8 = Sn_{11}R_{12}$ — **73**, R = *t*Bu

Scheme S 2 Structural relationship between so-far isolated propellane structures and **2**.

6.2.3 *$Sn_{16}tBu_{16}$ (3)*

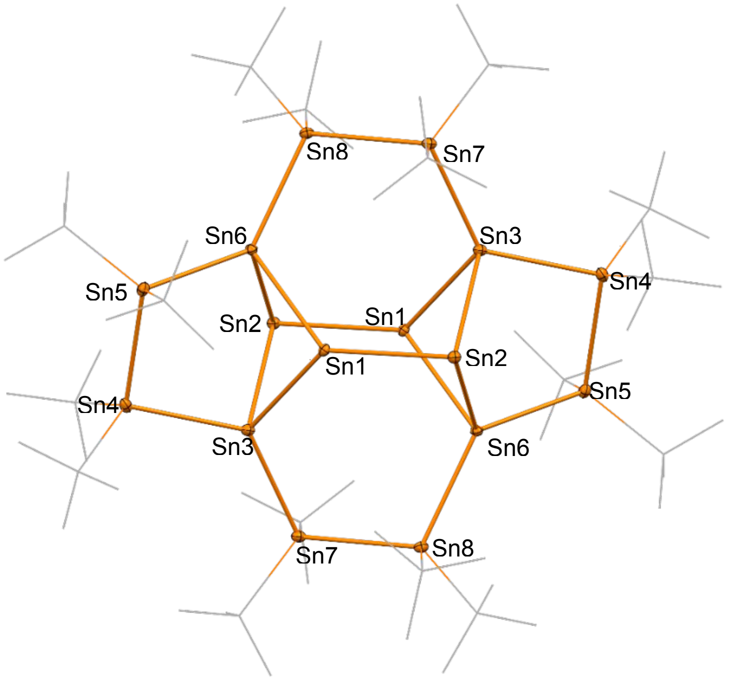

Figure S 22 Molecular structure of Sn_{16}*t*Bu_{16} (**3**). All non-carbons shown as 30% shaded ellipsoids. Hydrogen atoms and solvent molecules are omitted for clarity. Selected bond lengths [Å] and angles [°] for **3**: Sn1···Sn2 3.2758(4), Sn1···Sn1# 3.0595(5), Sn1-Sn2# 2.9016(4), Sn1-Sn3 2.8028(4), Sn1-Sn6 2.8044(4), Sn2-Sn3 2.8971(4), Sn2-Sn6 2.8853(5), Sn3-Sn4 2.8288(5), Sn3-Sn7 2.8132(4), Sn4-Sn5 2.8538(4), Sn5-Sn6 2.8189(5), Sn6-Sn8# 2.8233(4), Sn7-Sn8 2.8285(4), Sn1-Sn3-Sn2 70.14(1), Sn1-Sn6-Sn2 70.29(1), Sn3-Sn1-Sn2# 130.22(1), Sn3-Sn1-Sn6 91.64(1), Sn3-Sn2-Sn1# 92.43(1), Sn3-Sn4-Sn5 99.23(1), Sn3-Sn7-Sn8 108.53(1), Sn4-Sn3-Sn7 129.61(1), Sn1-Sn2#···Sn1# 59.01(1), Sn2···Sn1-Sn2# 120.99(1), C-Sn4-C 108.1(2), C-Sn7-C 111.1(2).

The larger isolated cluster $Sn_{16}tBu_{16}$ (**3**) crystallizes from DME solutions as small green needles in space group $P\bar{1}$ with one solvent molecule in its asymmetric unit. Due to the highly pronounced torsion angles of tBu$_2$SnSntBu$_2$ bridges (24.22(1)°, 42.92(2)°), **3** possesses only a C_2 geometry. Its Sn_{16} core contains eight Sn^0 atoms, in which the four central tin atoms form a rhombus structure (Sn1, Sn1$^\#$, Sn2 and Sn2$^\#$) similarly to recently isolated tin clusters $Sn_8Ar^{Me6}{}_4$ and $Sn_{10}Tripp_8$[8,9] (Figure S 22). Bonding of this core structure was also discussed within the scope of these publications. Yet, the nature of bonding in these structures could not be ascertained by classical bond models by all means. In **3**, the Sn_4 core structure is complexed by two Sn-tBu$_2$Sn-SntBu$_2$-Sn fragments (Sn3-tBu$_2$Sn7-tBu$_2$Sn8-Sn6$^\#$ and Sn6-tBu$_2$Sn8-tBu$_2$Sn7$^\#$-Sn3, respectively) resulting in two butterfly-type four membered rings (Sn1Sn3Sn2Sn6 and Sn1$^\#$Sn3$^\#$Sn2$^\#$Sn6$^\#$), which are additionally capped with tBu$_2$SnSntBu$_2$ entities. Alternatively, **3** might be described as four five as well as four six membered rings sharing edges. Overall, mean Sn-Sn bond distances between Sn^0 atoms as well as between tBu$_2$Sn and Sn^0 atoms in **3** are elongated (2.8655(1) Å, 2.8210(9) Å) when compared to **2** (2.817(4) Å, 2.802(7) Å). This observation is accompanied by the shortened Sn-Sn bonds of tBu$_2$SnSntBu$_2$ units (average value of 2.8411(9) Å for **3**, 2.910(5) Å for **2**). Interestingly, the Sn1-Sn3 and Sn1-Sn6 bond distances (2.8028(4) Å, 2.8044(4) Å) in **3** are shorter than any bond in **2**, resembling bond lengths of α-tin (2.80 Å).[7] Similar to bridgehead atoms in propellane type structures, all Sn-Sn-Sn bond angles around Sn2 and Sn2# approach angles around 90° (Sn3-Sn2-Sn6 88.12(1)°, Sn1-Sn2-Sn3 92.43(1)°, Sn1-Sn2-Sn6 92.48(1)°) indicating a high p character for bonding orbitals and a high s character for non-bonding electrons ('lone pair'). Angles around Sn1 and Sn1# respectively tend to be widened (Sn3-Sn1-Sn6 91.64(1)°, Sn2-Sn1-Sn3 130.22(1)°, Sn2-Sn1-Sn6 130.24(1)°). In accordance with observations in **2**, tBu$_2$Sn-Sn-SntBu$_2$ angles of Sn3, Sn6 and equivalents # are widened (Sn4-Sn3-Sn7 129.61(1)°, Sn5-Sn6-Sn8 131.39(1)°), whereas Sn^0-Sn-Sn^0 angles are acute (Sn1-Sn3-Sn2 70.14(1)°, Sn1-Sn6-Sn2 88.12(1)°). Although all tBu substituted tin atoms in **3** exhibit nearly ideal tetrahedral C-Sn-C angles (108.1(2) to 111.1(2)°), Sn-Sn-Sn angles of Sn4 and Sn5 are more acute (Sn3-Sn4-Sn5 99.23(1)°, Sn4-Sn5-Sn6 101.06(1)°) than found around Sn7 and Sn8 (Sn3-Sn7-Sn8 108.53(1)°, Sn7-Sn8-Sn6# 108.57(1)°) agreeing with expected overall less acute angles in a regular six membered ring (120°) compared to a regular pentagon (108°).

6.2.4 ***$Sn_{15}tBu_{14}$ (4)***

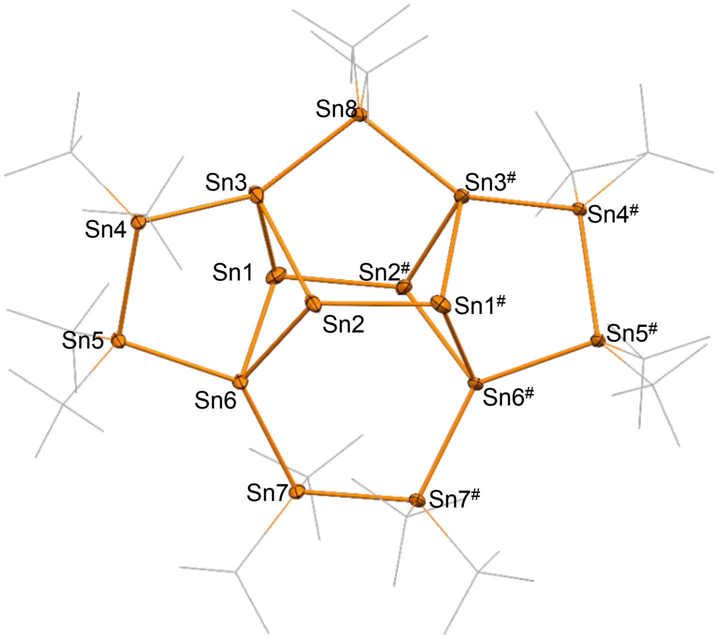

Figure S 23 Molecular structure of $Sn_{15}tBu_{14}$ (**4**). All non-carbons shown as 30% shaded ellipsoids. Hydrogen atoms are omitted for clarity. Selected bond lengths [Å] and angles [°] for **4**: Sn1-Sn2 2.863(1), Sn1···Sn2# 3.279(1), Sn1-Sn3 2.874(1), Sn1-Sn6 2.900(1), Sn2···Sn2# 3.0946(9), Sn2-Sn3 2.788(1), Sn2-Sn6 2.7634(9), Sn3-Sn4 2.8171(9), Sn3-Sn8 2.8327(8), Sn4-Sn5 2.896(4), Sn5-Sn6 2.803(4), Sn6-Sn7 2.8146(8), Sn7-Sn7# 2.8500(8), Sn1-Sn3-Sn2 70.74(3), Sn1-Sn6-Sn2 70.70(2), Sn3-Sn1-Sn2# 89.46(3), Sn3-Sn1-Sn6 90.46(3), Sn3-Sn2-Sn6 95.18(3), Sn3-Sn2-Sn1# 121.11(3), Sn3-Sn4-Sn5 98.68(9), Sn3-Sn8-Sn3# 102.94(4), Sn4-Sn3-Sn8 142.98(3), Sn4-Sn5-Sn6 101.65(8), Sn5-Sn6-Sn7 136.16(6), Sn6-Sn7-Sn7# 108.73(2), Sn1···Sn2-Sn1# 119.76(3), Sn2···Sn1-Sn2# 60.07(3), C-Sn4-C 107.8(5), C-Sn5-C 108.2(13), C-Sn7-C 111.2(4), C-Sn8-C 115.0(7).

Cluster $Sn_{15}tBu_{14}$ (**4**) displays the same structural core motif as **3** (Figure S 23). Yet, one ditin bridge $tBu_2SnSntBu_2$ is replaced by a single di-*tert*-butyl stannylene (tBu_2Sn) bridge leading to a polyhedral arrangement of six five membered rings and two six membered rings sharing edges. Named tBu_2Sn bridge (Sn8) exhibits a slightly widened Sn-Sn-Sn angle (Sn3-Sn8-Sn3# 102.94(4)°) compared to Sn-Sn-Sn angles in the adjacent ditinbridge (Sn3-Sn4-Sn5 98.68(9)°, Sn4-Sn5-Sn6 101.7(1)°). In agreement with expected less acute angles in regular hexagons, Sn-Sn-Sn angles in ditinbridges engaged in a six membered ring arrangement are significantly wider than the corresponding angle observed for Sn8 (Sn6-Sn7-Sn7# 108.73(2)°). Also, Sn-Sn bond distances of Sn8 are slightly elongated (Sn8-Sn3 2.8327(8) Å) when compared to the corresponding bonds in ditin bridges of **4** (Sn7-Sn6 2.8146(8) Å) indicating only insignificantly constrained geometry around Sn8. Unsurprisingly, substitution of the ditin bridge by a single stannylene bridge results in a widened tBu_2Sn-Sn-$SntBu_2$ angle (Sn3-Sn4-Sn8 142.98(8)°) compared to the opposite angle of 136.16(9) for Sn5-Sn6-Sn7. In general, corresponding angles in structural related **3** are less widened (Sn4-Sn3-Sn8# 129.61(1)°, Sn5-Sn6-Sn7 131.39(1)°). While Sn-Sn bonds of Sn^0 atoms in **4** (2.811(7) Å, 2.837(8) Å) are on average shorter compared to bond lengths in **3** (2.8210(9) Å, 2.8655(1) Å), bond distances in $tBu_2SnSntBu_2$ units are slightly elongated (**4**: avg. 2.8809(6) Å; **3**: avg. 2.8411(9) Å). Notably, bond distances of Sn2-Sn3 (2.788(1) Å) and Sn2-Sn6 (2.7634(9) Å), respectively, are significantly shortened compared to the mean Sn^0-Sn^0 bond length in **4**. Similarly, such shortened bonds were also found in **3**. Aside from mentioned values, **4** does not show any structural particularities to **3**. The isolated clusters $Sn_{16}tBu_{16}$ (**3**) and $Sn_{15}tBu_{14}$ (**4**) do not show any structural similarities to clusters of similar size like $Sn_{15}(N(SiMe_2Ph)Dipp)_6$, $Sn_{17}[GaCl(dpp)]_4$ and $Sn_{20}(SitBu_3)_{10}Cl_2$.[10–12] The latter two both exhibit Sn9 subunits interconnected in different ways.

6.2.5 *$tBu_6Sn_3PbSn_3tBu_6$ (6)*

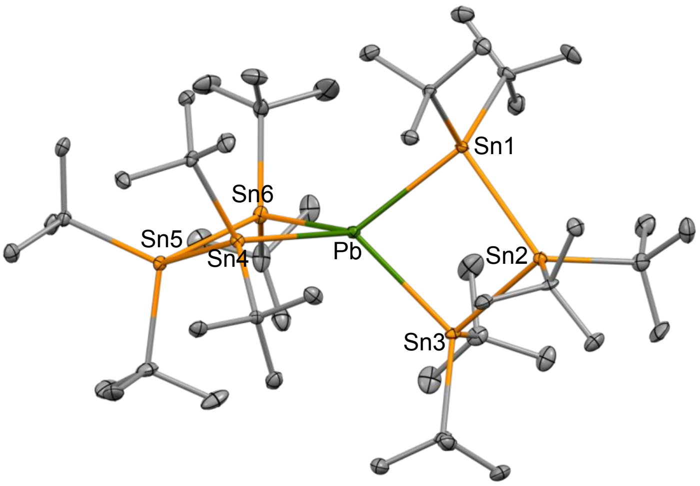

Figure S 24 Molecular structure of tBu$_6$Sn$_3$PbSn$_3t$Bu$_6$ (**6**). All non-carbons shown as 30% shaded ellipsoids. Hydrogen atoms are omitted for clarity. Selected bond lengths [Å] and angles [°] for **6**: Pb-Sn1 2.9745(7), Pb-Sn4 2.9706(8), Sn1-Sn2 2.8554(8), Sn1⋯Sn3 4.065, Pb⋯Sn2 4.099, Pb⋯Sn5 4.162, Sn4-Sn5 2.8527(7), Sn1-C1 2.260(8), Sn1-Pb-Sn3 85.73(2), Sn1-Sn2-Sn3 90.61(2), Pb-Sn1-Sn2 89.33(2), Sn4-Pb-Sn6 84.99(2), Sn4-Sn5-Sn6 89.03(2), Pb-Sn4-Sn5 91.20(2), Pb-Sn1-C1 130.3(2), Pb-Sn4-C25 102.1(2).

6.2.6 *cyclo-Sn_4tBu_8 (5)*

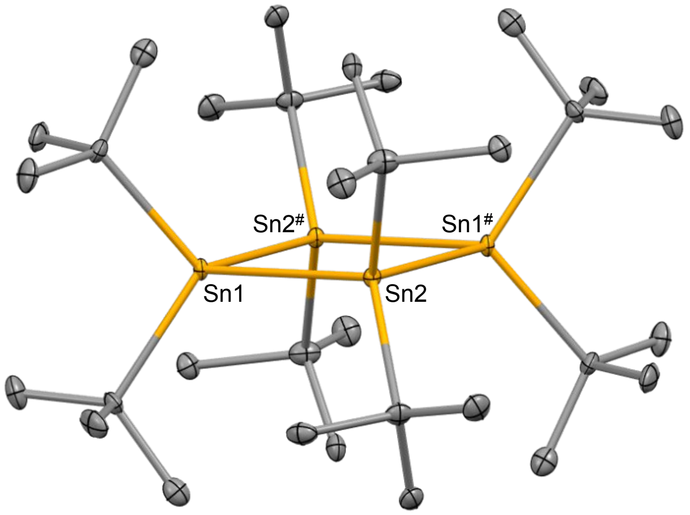

Figure S 25 Molecular structure of Sn$_4t$Bu$_8$ (**5**). All non-carbons shown as 30% shaded ellipsoids. Hydrogen atoms are omitted for clarity. Selected bond lengths [Å] and angles [°] for **5**: Sn1-Sn2 2.8915, Sn1-Sn2-Sn1# 90.13.

7 Quantum Chemical Investigations

7.1 Molecular Orbital (MO) Analysis

Table S 5 Calculated HOMO and LUMO energies [eV] and energy difference ($E_{LUMO-HOMO}$) of Sn_{11}*t*Bu_{12}, Sn_{15}*t*Bu_{14} and Sn_{16}*t*Bu_{16}.

E	Sn_{11}*t*Bu_{12}	Sn_{15}*t*Bu_{14}	Sn_{16}*t*Bu_{16}	*t*$Bu_6Sn_3PbSn_3$*t*Bu_6
**LUMO**	-2.475	-2.327	-2.402	-1.434
**HOMO**	-5.501	-4.981	-5.055	-5.613
**$\Delta E_{HOMO-LUMO}$**	3.026	2.654	2.653	4.179

7.1.1 *$Sn_{11}tBu_{12}$ (2)*

Table S 6 Summary of calculated excitations of **2**.

λ [nm]	**f**	
593	0.0000	473 → 474<br>(HOMO→ LUMO)
593	0.0000	472 → 474
527	0.0373	471 → 474
527	0.0373	470 → 474
448	0.0102	469 → 474
432	0.0000	468 → 474
432	0.0000	467 → 474
432	0.0000	466 → 474
365	0.0168	472 → 475
365	0.0168	473 → 475

LUMO (474)
-2.475 eV

HOMO (473)
-5.501 eV

HOMO-1 (472)
-5.502 eV

HOMO-2 (471)
-5.629 eV

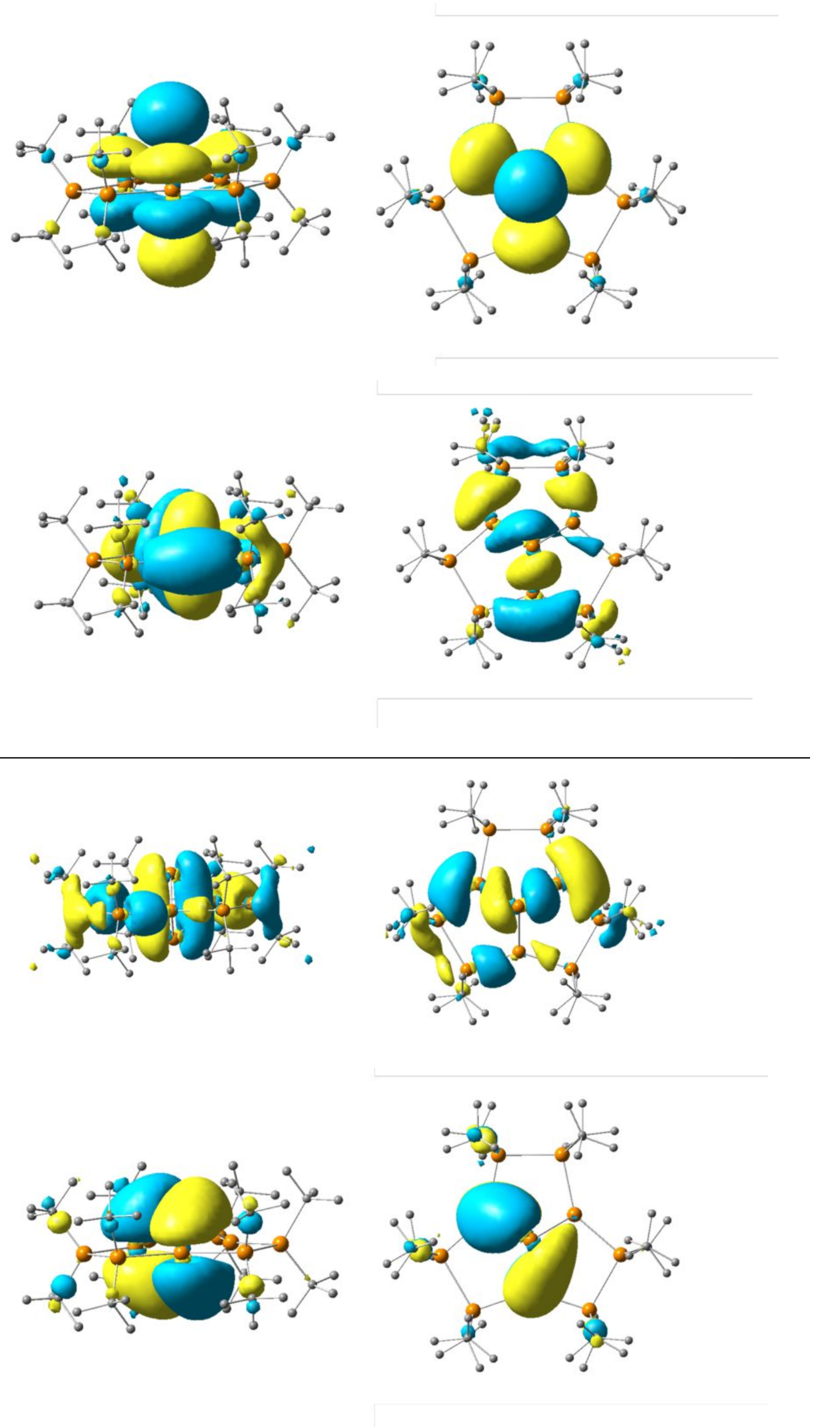

Figure S 26 Visual representation of LUMO, HOMO, HOMO-1 and HOMO-2 of Sn_{11}*t*Bu_{12} (**2**).

HOMO-3 (470)
-5.629 eV

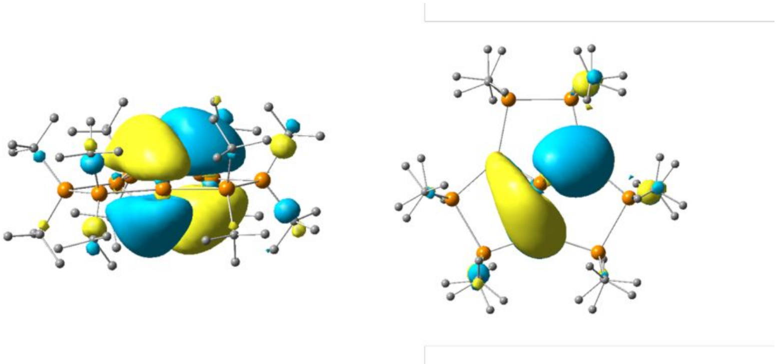

HOMO-4 (469)
-5.772 eV

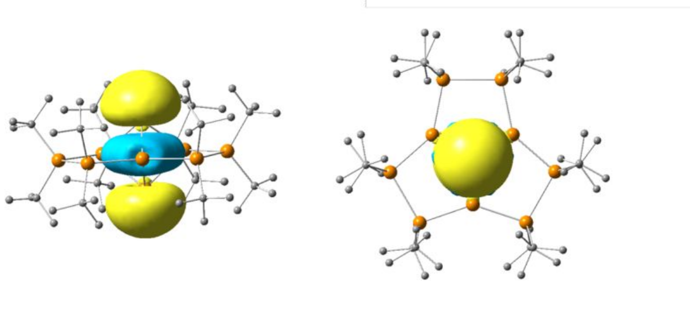

HOMO-17 (456)
-8.291 eV

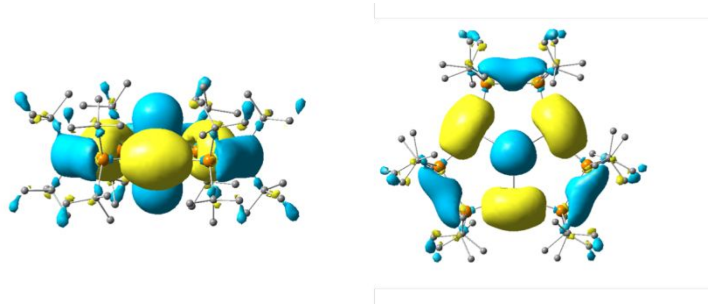

HOMO-45 (428)
-10.055 eV

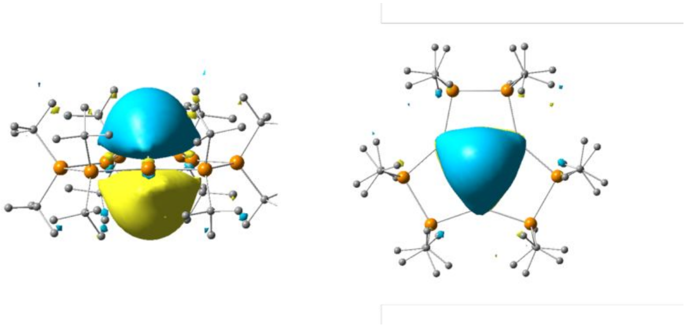

HOMO-120 (353)
-13.331 eV

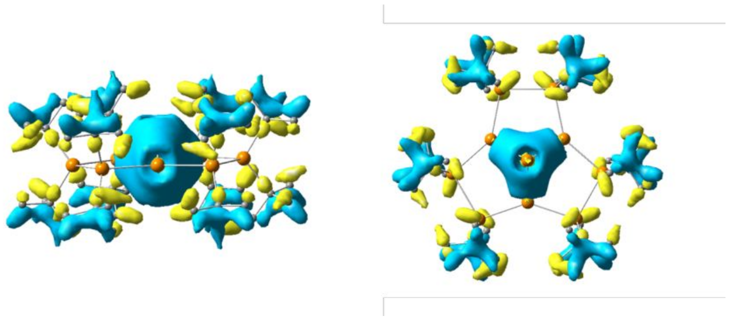

Figure S 27 Visual representation of HOMO-3, HOMO-4, HOMO-17, HOMO-45 and HOMO-120 of Sn_{11}*t*Bu_{12} (**2**).

7.1.2 *$Sn_{16}tBu_{16}$ (3)*

Table S 7 Summary of calculated excitations of **3**.

λ [nm]	**f**	
602	0.0868	664 → 665<br>(HOMO → LUMO)
579	0.0000	663 → 665
559	0.0352	662 → 665
511	0.0000	661 → 665
466	0.0000	660 → 665
457	0.0083	664 → 666
439	0.0000	664 → 667
433	0.0154	658 → 665<br>659 →665<br>663 → 667
417	0.0038	654 → 665<br>656 → 665<br>658 → 665<br>659 → 665<br>663 → 667
415	0.0067	656 → 665<br>658 → 665<br>662 → 666

LUMO (665)
-2.402 eV

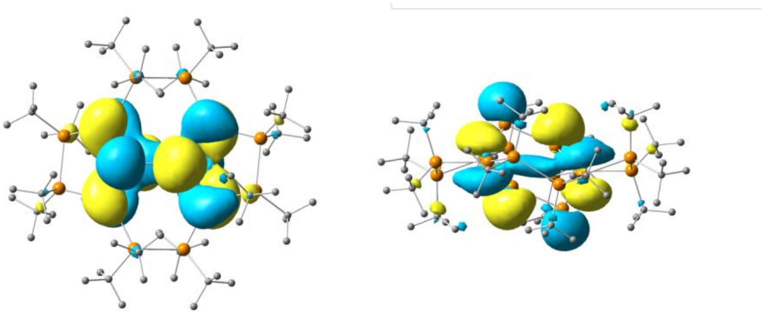

HOMO (664)
-5.055 eV

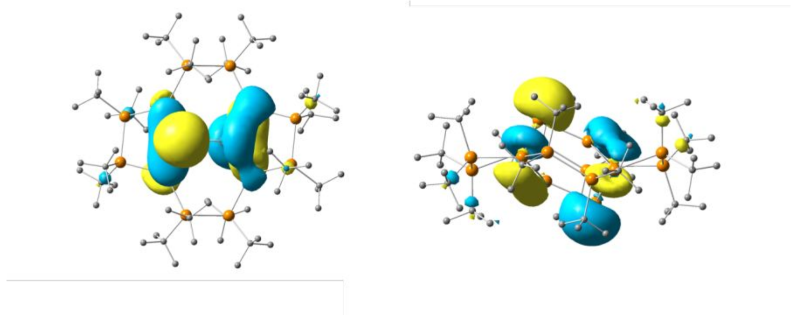

HOMO-1 (663)
-5.321 eV

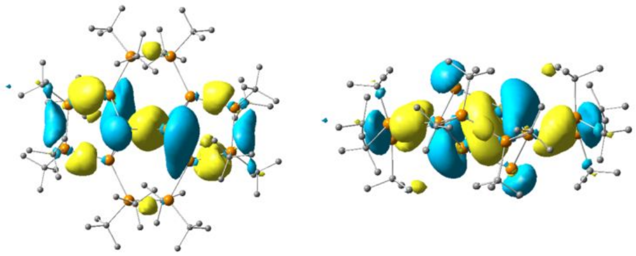

HOMO-2 (662)
-5.335 eV

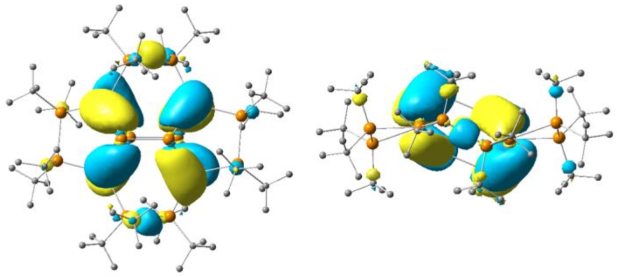

HOMO-3 (661)
-5.641 eV

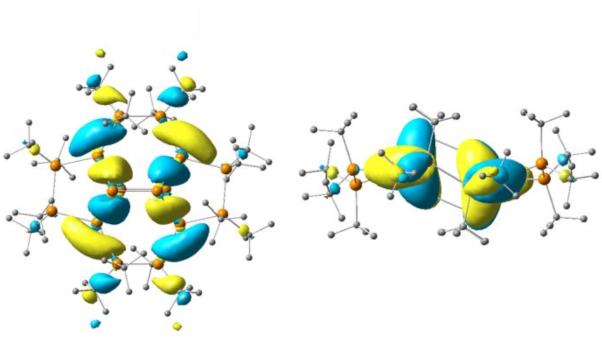

Figure S 28 Visual representation of LUMO and HOMO to HOMO-3 of $Sn_{16}t$$Bu_{16}$ (**3**).

HOMO-4 (660)
-5.830 eV

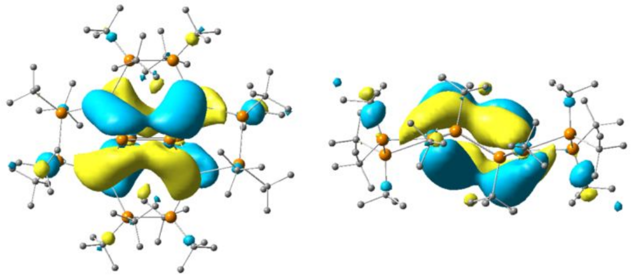

HOMO-10 (654)
-7.531 eV

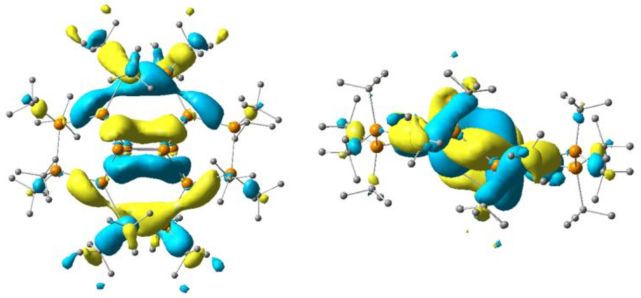

HOMO-163 (501)
-13.556 eV

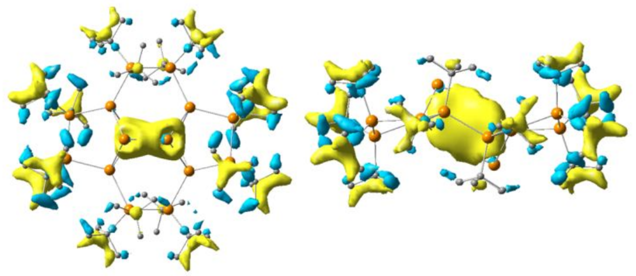

HOMO-167 (497)
-14.439 eV

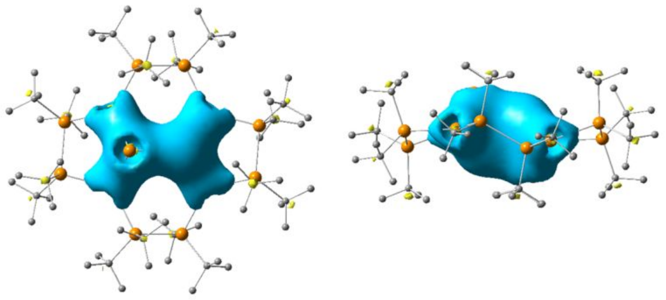

Figure S 29 Visual representation of HOMO-4, HOMO-10, HOMO-163 and HOMO-167 of $Sn_{16}tBu_{16}$ (**3**).

7.1.3 *$Sn_{15}tBu_{14}$ (4)*

Table S 8 Summary of calculated excitations of **4**.

λ [nm]	f	
606	0.0699	606 → 607<br>(HOMO → LUMO)
579	0.0001	605 → 607
527	0.0030	603 → 607
512	0.0372	604 → 607
490	0.0000	606 → 608
453	0.0055	606 → 609
450	0.0048	601 → 607<br>602 → 607<br>605 → 608
441	0.0004	600 → 607<br>603 → 608
433	0.0154	599 → 607<br>601 →> 607<br>602 → 607<br>604 → 608
427	0.0055	605 → 608<br>606 → 611<br>606 → 612

LUMO (607)
-2.327 eV

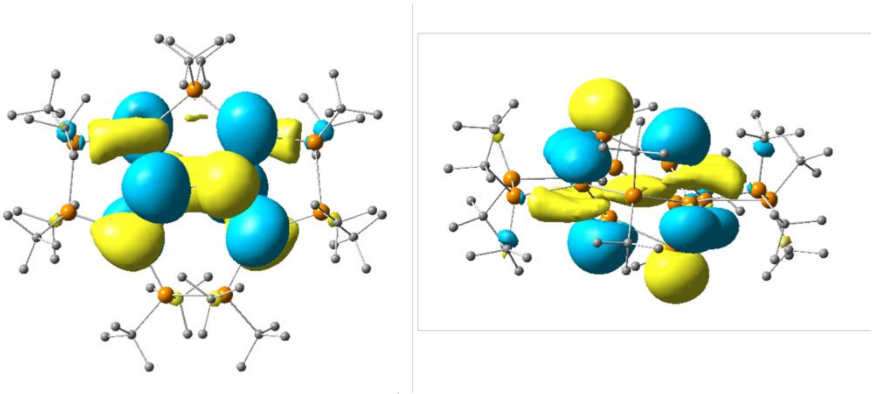

HOMO (606)
-4.981 eV

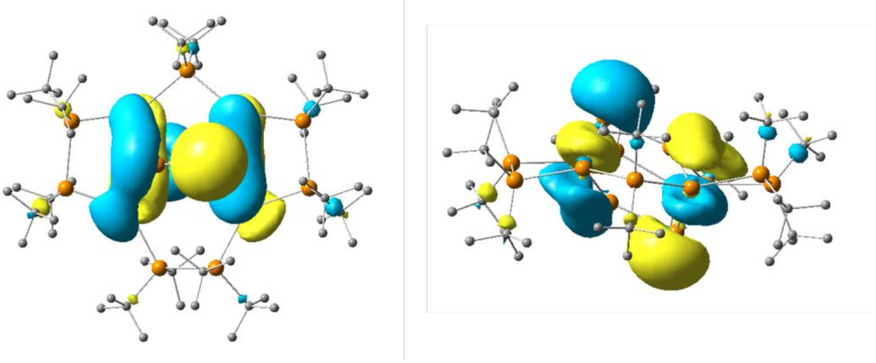

HOMO-1 (605)
-5.264 eV

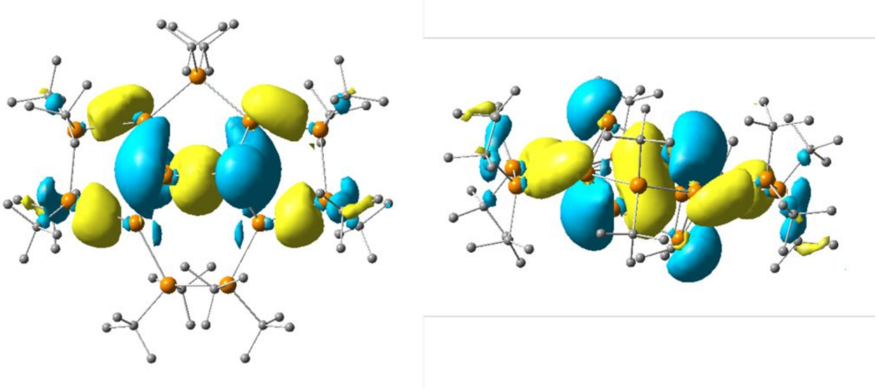

HOMO-2 (604)
-5.455 eV

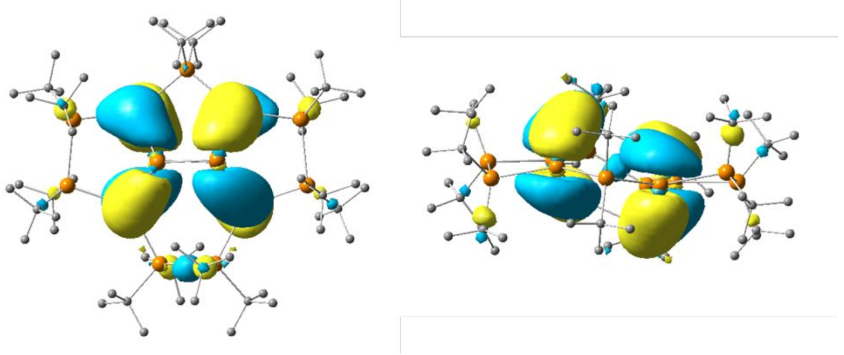

Figure S 30 Visual representation of LUMO and HOMO to HOMO-2 of $Sn_{15}tBu_{14}$ (**4**).

HOMO-3 (603)
-5.510 eV

HOMO-4 (602)
-5.923 eV

HOMO-6 (600)
-5.965 eV

HOMO-10 (596)
-6.460 eV

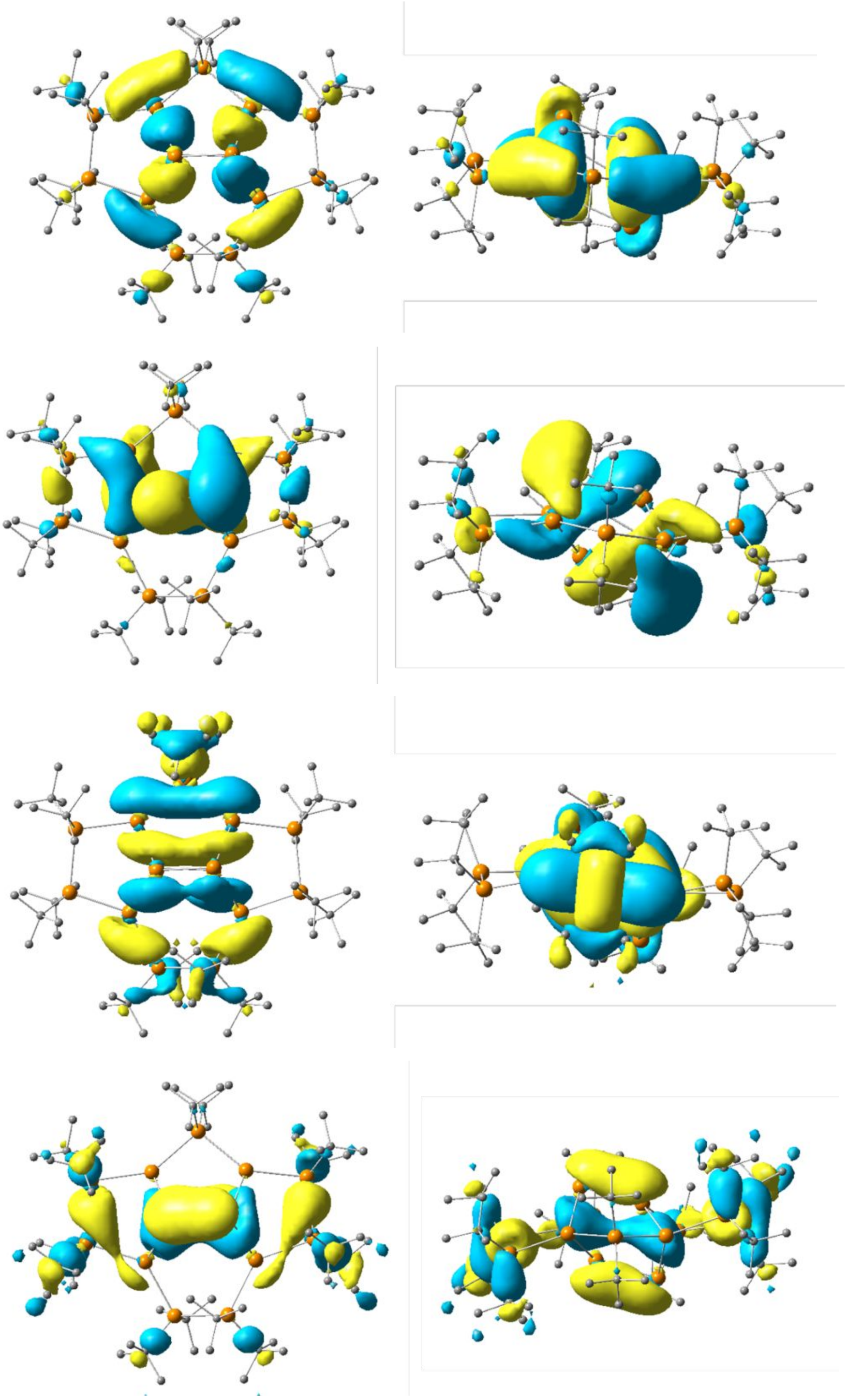

Figure S 31 Visual representation of HOMO-3, HOMO-4, HOMO-6 and HOMO-10 of $Sn_{15}\mathit{t}Bu_{14}$ (**4**).

HOMO-22 (584)
-8.038 eV

HOMO-101 (505)
-11.165 eV

HOMO-103 (503)
-11.393 eV

HOMO-147 (459)
-13.886 eV

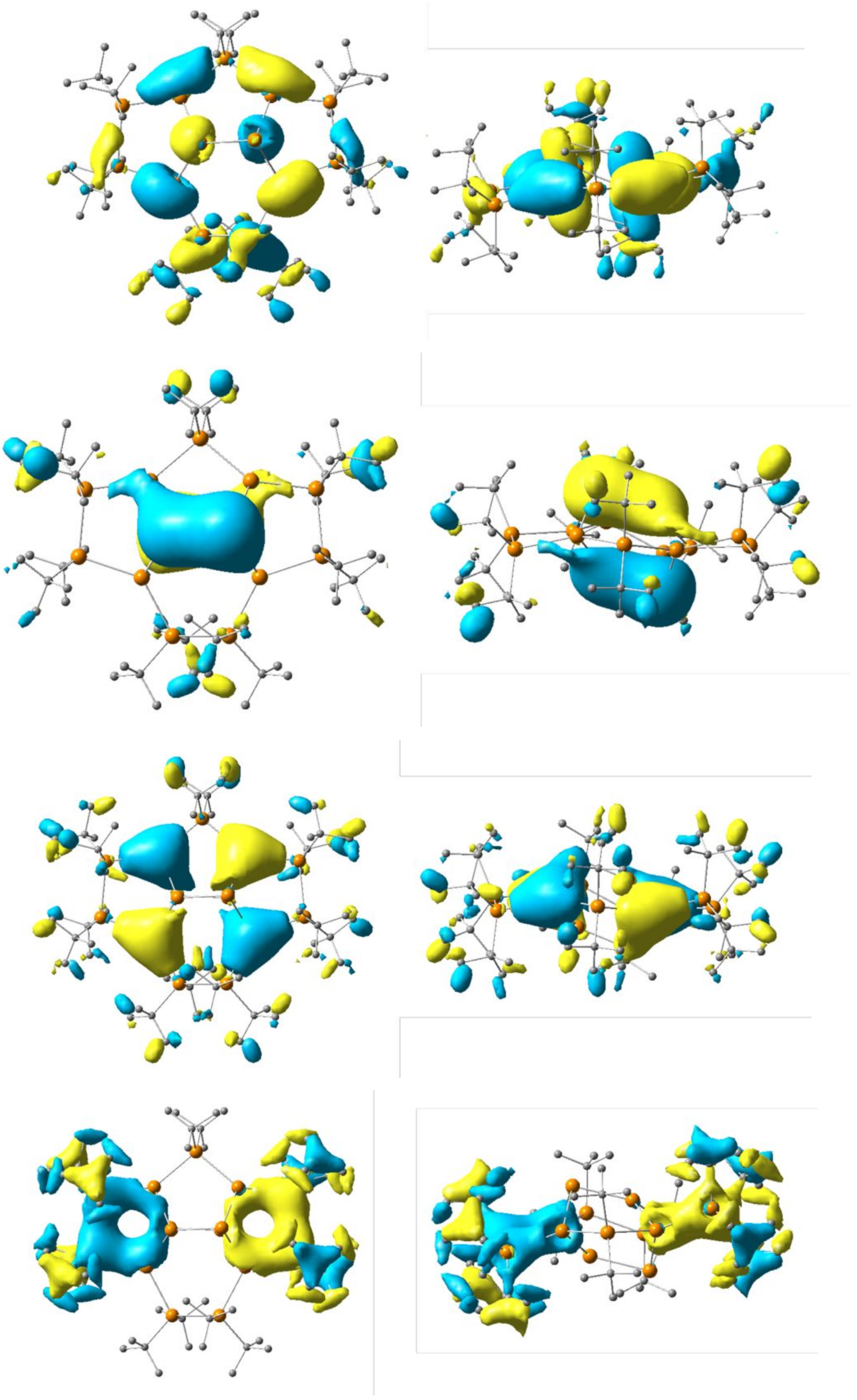

Figure S 32 Visual representation of HOMO-22, HOMO-101, HOMO-103 and HOMO-147 of Sn_{15}*t*Bu_{14} (**4**).

HOMO-148 (458)
-14.427 eV

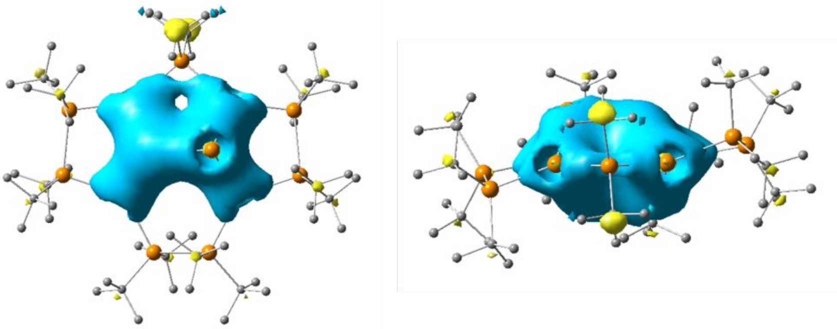

Figure S 33 Visual representation of HOMO-148 of $Sn_{15}tBu_{14}$ (**4**).

7.1.4 *tBu$_6$Sn$_3$PbSn$_3$tBu$_6$ (6)*

Table S 9 Summary of calculated excitations of **6**.

λ [nm]	**f**	
374	0.0081	387 → 390 (HOMO-2 → LUMO)<br>387 → 391 (HOMO-2 → LUMO+1)<br>388 → 391 (HOMO-1 → LUMO+1)<br>389 → 390 (HOMO → LUMO)<br>389 → 391 (HOMO → LUMO+1)
372	0.0039	387 → 390<br>388 → 390<br>388 → 391<br>389 → 390<br>389 → 391
367	0.0059	387 → 390<br>388 → 390<br>388 → 391<br>389 → 391
364	0.0021	387 → 390<br>387 →391<br>388 →390<br>388 → 391<br>389 → 390<br>389 → 391
342	0.0013	385 → 390<br>386 → 391<br>387 → 390<br>387 → 391<br>388 → 390<br>388 → 391
330	0.2304	385 → 390<br>386 → 390<br>387 → 390<br>387 → 391<br>388 → 390<br>388 → 391<br>389 → 392
329	0.0379	386 → 390<br>386 → 391<br>387 → 390<br>388 → 391
325	0.0157	385 → 390<br>385 → 391<br>386 → 391<br>387 → 391<br>388 → 391
316	0.0036	385 → 390<br>385 → 391<br>386 → 390<br>386 → 391<br>388 → 391
311	0.0408	385 → 390<br>386 → 391<br>389 → 392

LUMO+3 (393)
-0.403 eV

LUMO+2 (392)
-0.480 eV

LUMO+1 (391)
-1.398 eV

LUMO (390)
-1.434 eV

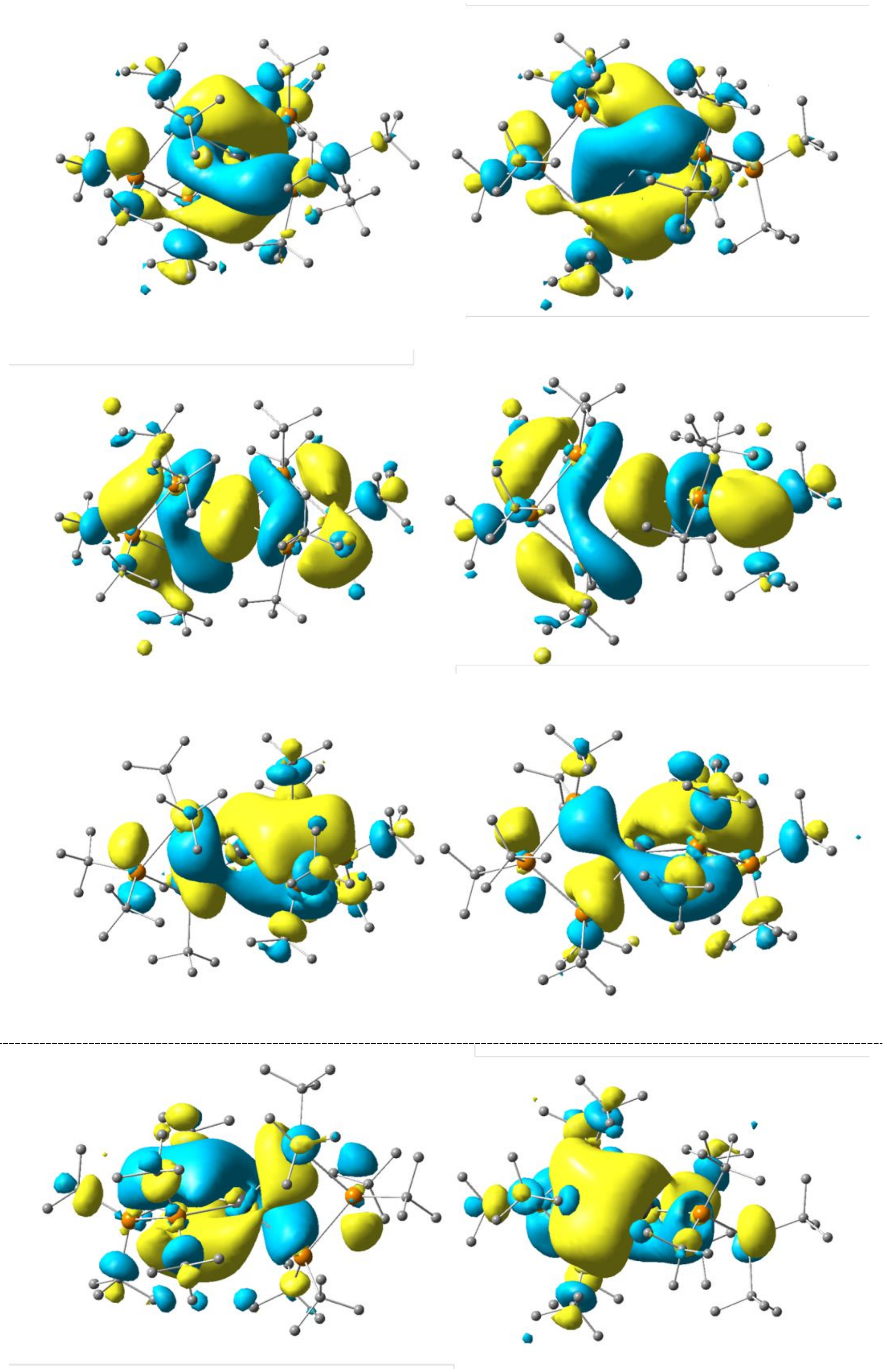

Figure S 34 Visual representation of LUMO to LUMO+3 of tBu$_6$Sn$_3$PbSn$_3$$tBu_6$ (**6**).

HOMO (389)
-5.613 eV

HOMO-1 (388)
-5.796 eV

HOMO-2 (387)
-5.836 eV

HOMO-3 (386)
-6.049 eV

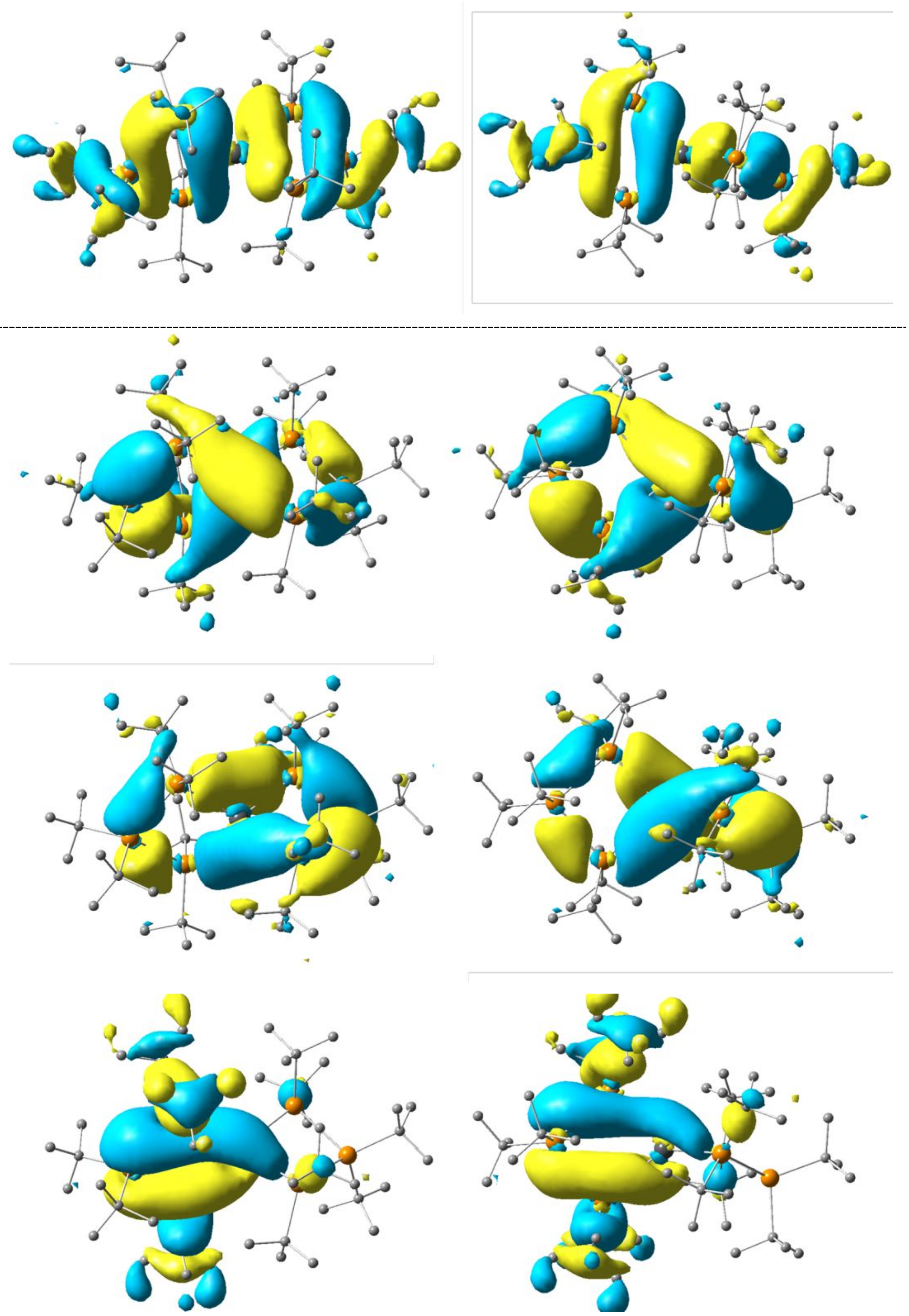

Figure S 35 Visual representation of HOMO to HOMO-3 of *t*$Bu_6Sn_3PbSn_3$*t*Bu_6 (**6**).

HOMO-4 (385)
-6.072 eV

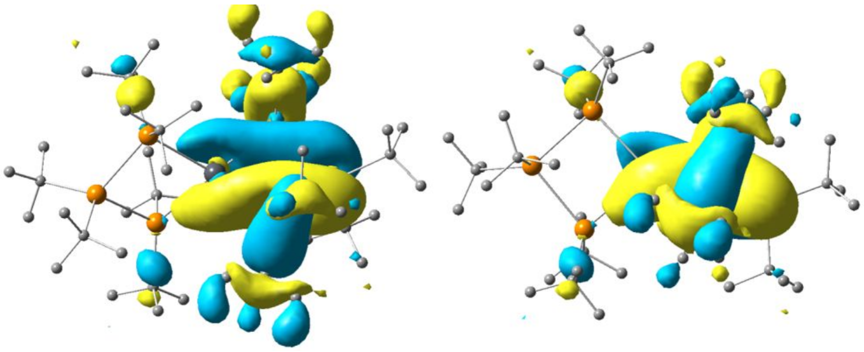

HOMO-5 (384)
-6.502 eV

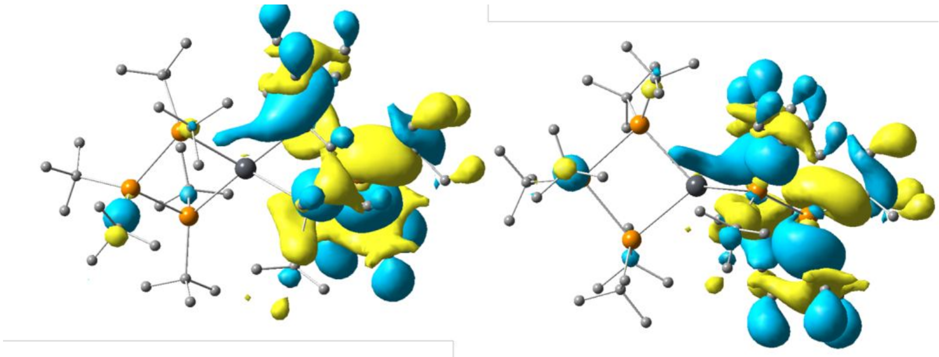

HOMO-6 (383)
-6.515 eV

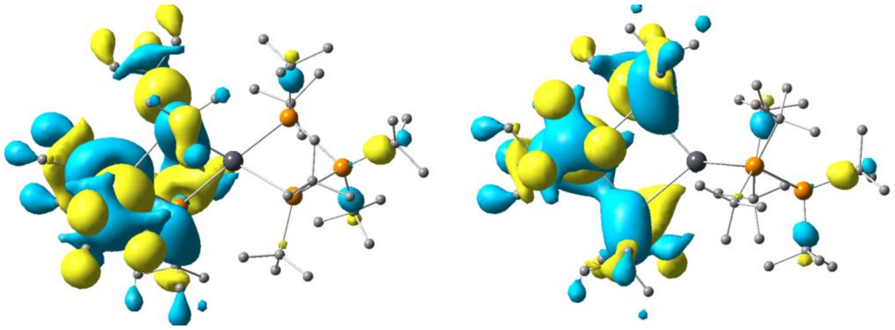

Figure S 36 Visual representation of HOMO-4 to HOMO-6 of tBu$_6$Sn$_3$PbSn$_3$$tBu_6$ (**6**).

7.2 Quantum Theory of Atoms in Molecules (QTAIM) / Bader and Natural Bond Orbital (NBO) Analyses

7.2.1 *$Sn_{11}tBu_{12}$ (2)*

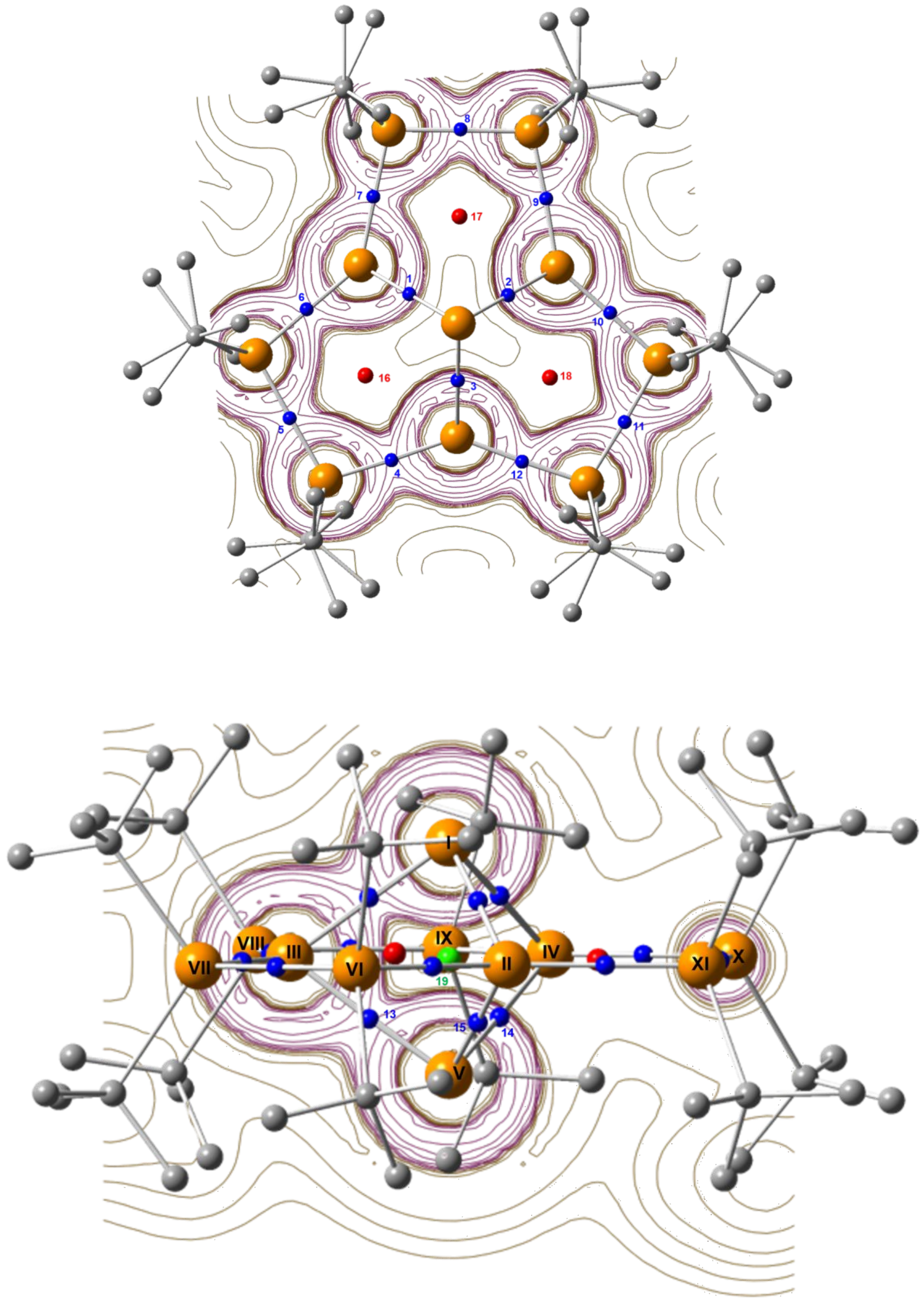

Figure S 37 Contour plot of the Laplacian ($\nabla^2\rho$) in top view (top) and contour plot of the Laplacian ($\nabla^2\rho$) in side view (buttom). Tin atoms are numbered in roman letters, symmetry generated atoms are marked by #. Critical points (CPs) are color coded: Bond critical points (BCPs): Blue; ring critical points (RCPs): Red; cage critical points (CCPs): Green.

Table S 10 Summary of calculated AIM and NBO charges for tin atoms in **2**.

Tin atom #	AIM charge	NBO charge
**I**	0.157	0.071
**II**	-0.155	-0.328
**III**	-0.155	-0.329
**IV**	-0.154	-0.328
**V**	0.158	0.071
**VI**	1.262	1.005
**VII**	1.261	1.005
**VIII**	1.261	1.005
**IX**	1.261	1.005
**X**	1.260	1.005
**XI**	1.261	1.005

Table S 11 Summary of electron density (ρ [a.u.]) and Laplacian of electron density ($\nabla^2\rho$ [a.u.]) for CPs in **2** as determined by QTAIM analysis.

Critical point #		ρ [a.u.]	$\nabla^2\rho$ [a.u.]
**Bond critical points (BCPs)**	**1**	0.0428	0.0207
	**2**	0.0428	0.0206
	**3**	0.0428	0.0207
	**4**	0.0464	0.0135
	**5**	0.0403	0.0058
	**6**	0.0463	0.0134
	**7**	0.0464	0.0135
	**8**	0.0403	0.0058
	**9**	0.0464	0.0135
	**10**	0.0464	0.0135
	**11**	0.0403	0.0058
	**12**	0.0463	0.0134
	**13**	0.0428	0.0206
	**14**	0.0428	0.0206
	**15**	0.0428	0.0207
**Ring critical points (RCPs)**	**16**	0.0065	0.0137
	**17**	0.0065	0.0137
	**18**	0.0065	0.0137
**Cage critical points (CCPs)**	**19**	0.0192	0.0307

7.2.2 *Sn$_{16}$tBu$_{16}$ (3)*

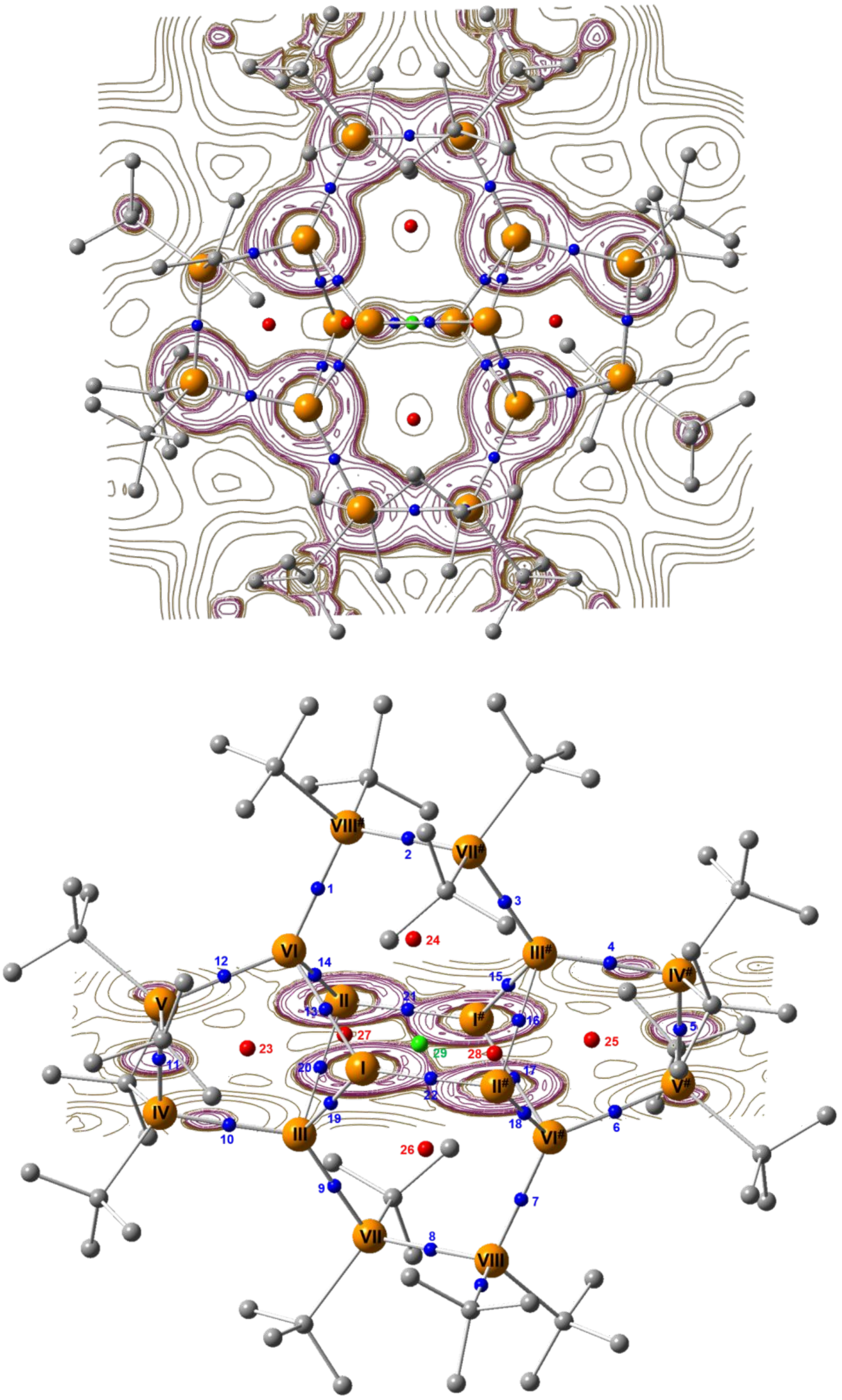

Figure S 38 Contour plot of the Laplacian (∇²ρ) in top view (top) and contour plot of the Laplacian (∇²ρ) in side view (buttom). Tin atoms are numbered in roman letters, symmetry generated atoms are marked by #. Critical points (CPs) are color coded: Bond critical points (BCPs): Blue; ring critical points (RCPs): Red; cage critical points (CCPs): Green.

Table S 12 Summary of calculated AIM and NBO charges for tin atoms in **3**.

Tin atom #	AIM charge	NBO charge
**I** (= I#)	0.018	-0.009
**II** (= II#)	0.325	0.046
**III** (= III#)	-0.095	-0.275
**IV** (= IV#)	1.217	0.981
**V** (= V#)	1.225	0.972
**VI** (= VI#)	-0.116	-0.262
**VII** (= VII#)	1.245	0.986
**VIII** (= VIII#)	1.244	0.983

Table S 13 Summary of electron density (ρ [a.u.]) and Laplacian of electron density ($\nabla^2\rho$ [a.u.]) for CPs in **3** as determined by QTAIM analysis.

Critical point #		ρ [a.u.]	$\nabla^2\rho$ [a.u.]
**Bond critical points (BCPs)**	1	0.0459	0.0125
	2	0.0453	0.0077
	3	0.0461	0.0127
	4	0.0447	0.0128
	5	0.0437	0.0075
	6	0.0453	0.0131
	7	0.0459	0.0125
	8	0.0453	0.0077
	9	0.0461	0.0127
	10	0.0447	0.0128
	11	0.0437	0.0075
	12	0.0453	0.0131
	13	0.0383	0.0178
	14	0.0443	0.0211
	15	0.0381	0.0172
	16	0.0450	0.0212
	17	0.0443	0.0211
	18	0.0383	0.0178
	19	0.0450	0.0212
	20	0.0381	0.0172
	21	0.0360	0.0219
	22	0.0360	0.0219
**Ring critical points (RCPs)**	23	0.0066	0.0141
	24	0.0032	0.0057
	25	0.0066	0.0141
	26	0.0032	0.0057
	27	0.0185	0.0278
	28	0.0185	0.0278
**Cage critical points (CCPs)**	29	0.0281	0.0150

7.2.3 *$Sn_{15}tBu_{14}$ (4)*

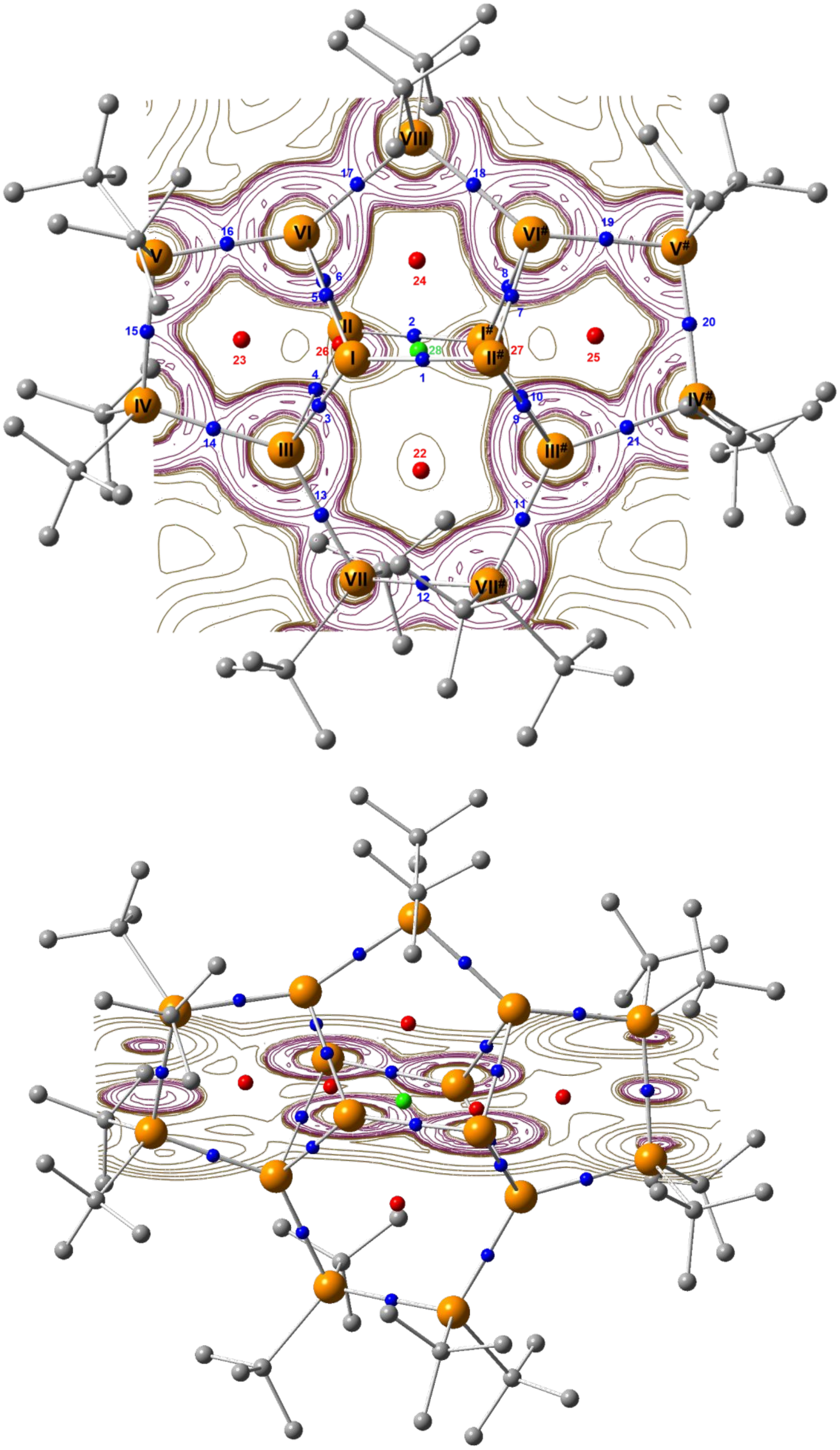

Figure S 39 Contour plot of the Laplacian (∇²ρ) in top view (top) and contour plot of the Laplacian (∇²ρ) in side view (buttom). Tin atoms are numbered in roman letters, symmetry generated atoms are marked by #. Critical points (CPs) are color coded: Bond critical points (BCPs): Blue; ring critical points (RCPs): Red; cage critical points (CCPs): Green.

Table S 14 Summary of calculated AIM and NBO charges for tin atoms in **4**.

Tin atom #	AIM charge	NBO charge
**I** (= I#)	-0.036	0.014
**II** (= II#)	0.293	0.045
**III** (= III#)	-0.103	-0.289
**IV** (= IV#)	1.249	1.006
**V** (= V#)	1.258	1.005
**VI** (= VI#)	-0.149	-0.332
**VII** (= VII#)	1.262	0.995
**VIII**	1.285	1.143

Table S 15 Summary of electron density (ρ [a.u.]) and Laplacian of electron density ($\nabla^2\rho$ [a.u.]) for CPs in **4** as determined by QTAIM analysis.

Critical point #		ρ [a.u.]	$\nabla^2\rho$ [a.u.]
**Bond critical points (BCPs)**	**1**	0.0396	0.0223
	**2**	0.0396	0.0223
	**3**	0.0478	0.0234
	**4**	0.0188	0.0174
	**5**	0.0042	0.0202
	**6**	0.0389	0.0183
	**7**	0.0389	0.0183
	**8**	0.0457	0.0213
	**9**	0.0376	0.0174
	**10**	0.0478	0.0234
	**11**	0.0457	0.0127
	**12**	0.0433	0.0066
	**13**	0.0457	0.0127
	**14**	0.0458	0.0135
	**15**	0.0405	0.0063
	**16**	0.0450	0.0143
	**17**	0.0440	0.0129
	**18**	0.0440	0.0129
	**19**	0.0450	0.0143
	**20**	0.0405	0.0063
	**21**	0.0458	0.0135
**Ring critical points (RCPs)**	**22**	0.0032	0.0057
	**23**	0.0064	0.0138
	**24**	0.0070	0.0141
	**25**	0.0064	0.0138
	**26**	0.0181	0.0273
	**27**	0.0181	0.0273
**Cage critical points (CCPs)**	**28**	0.0230	0.0148

8 References

(1) Puff, H.; Bach, C.; Schuh, W.; Zimmer, R. Bindungsabstände Zwischen Organylsubstituierten Zinnatomen II. Cyclo-Tetrastannane. *J. Organomet. Chem.* **1986**, *312*, 313–322. https://doi.org/10.1017/CBO9781107415324.004.

(2) Lechner, M. L.; Fürpaß, K.; Sykora, J.; Fischer, R. C.; Albering, J.; Uhlig, F. Functionalized Tetrastannacyclobutanes, Part I. *J. Organomet. Chem.* **2009**, *694* (26), 4209–4215. https://doi.org/10.1016/j.jorganchem.2009.09.004.

(3) Rannenberg, M.; Obermeyer, A. Methylzinn- Und Methylindium-Bis(Trimethylsilyl)Amide. *Zeitschrift für Naturforschung B* **1991**, *46b*, 459–467.

(4) Kanj, A.; Meunier, P.; Hanquet, B.; Gautheron, B.; Dubac, J.; Daran, J.-C. D. A.Kanj, P.Meunier, B.Hanquet, B.Gautheron, J.Dubac, J.-C.Daran. *Bull. Soc. Chim. Fr.* **1994**, *131*, 715.

(5) Jolly, B. S.; Lappert, M. F.; Engelhardt, L. M.; White, A. H.; Raston, C. L. Subvalent Group 14 Metal Compounds. Part 16. Synthesis, Crystal Structure and Characterisation of Some β-Functionalised-Alkyltin(II) Complexes, SnR(X) {R = $C_5H_4N[C(SiMe_3)_2]^{2-}$; X = R, Cl or $N(SiMe_3)_2$}. *J. Chem. Soc. Dalton Trans.* **1993**, No. 17, 2653–2663. https://doi.org/10.1039/DT9930002653.

(6) Sita, L. R.; Kinoshita, I. Contribution of Nonbonded Interactions to the Destabilization of a Group 14 Bicyclo[1.1.1]Pentane. *J. Am. Chem. Soc.* **1991**, *113* (13), 5070–5072. https://doi.org/10.1021/ja00013a058.

(7) Wells, A. F. *Structural Inorganic Chemistry*, 5th edition.; Clarendon Press: Oxford, **1984**.

(8) Eichler, B. E.; Power, P. P. Synthesis and Characterization of $[Sn_8(2,6\text{-}Mes_2C_6H_3)_4]$ (Mes=2,4,6-$Me_3C_6H_2$): A Main Group Metal Cluster with a Unique Structure. *Angew. Chem. Int. Ed.* **2001**, *40* (4), 796–797. https://doi.org/10.1002/1521-3773(20010216)40:4<796::AID-ANIE7960>3.0.CO;2-5.

(9) Wiederkehr, J.; Wölper, C.; Schulz, S. Synthesis and Solid State Structure of a Metalloid Tin Cluster [Sn10(Trip8)]. *Chem. Commun.* **2016**, *52* (83), 12282–12285. https://doi.org/10.1039/c6cc06770k.

(10) Brynda, M.; Herber, R.; Hitchcock, P. B.; Lappert, M. F.; Nowik, I.; Power, P. P.; Protchenko, A. V.; Růžička, A.; Steiner, J. Higher-Nuclearity Group 14 Metalloid Clusters: $[Sn_9\{Sn(NRR')\}_6]$. *Angew. Chem. Int. Ed.* **2006**, *45* (26), 4333–4337. https://doi.org/10.1002/anie.200600292.

(11) Prabusankar, G.; Kempter, A.; Gemel, C.; Schröter, M. K.; Fischer, R. A. $[Sn_{17}\{GaCl(Ddp)\}_4]$: A High-Nuclearity Metalloid Tin Cluster Trapped by Electrophilic Gallium Ligands. *Angew. Chem. Int. Ed.* **2008**, *47* (38), 7234–7237. https://doi.org/10.1002/anie.200802470.

(12) Binder, M.; Schrenk, C.; Schnepf, A. Sn20(SitBu3)10Cl2- the Largest Metalloid Group 14 Cluster Shows a Raspberry-like Arrangement of Smaller Units. *Chem. Commun.* **2019**, *55* (81), 12148–12151. https://doi.org/10.1039/c9cc07099k.
